# Clinical Outcomes, Costs, and Cost-effectiveness of Strategies for Adults Experiencing Sheltered Homelessness During the COVID-19 Pandemic

**DOI:** 10.1001/jamanetworkopen.2020.28195

**Published:** 2020-12-22

**Authors:** Travis P. Baggett, Justine A. Scott, Mylinh H. Le, Fatma M. Shebl, Christopher Panella, Elena Losina, Clare Flanagan, Jessie M. Gaeta, Anne Neilan, Emily P. Hyle, Amir Mohareb, Krishna P. Reddy, Mark J. Siedner, Guy Harling, Milton C. Weinstein, Andrea Ciaranello, Pooyan Kazemian, Kenneth A. Freedberg

**Affiliations:** 1 Division of General Internal Medicine, Massachusetts General Hospital, Boston; 2 Harvard Medical School, Boston, Massachusetts; 3 Institute for Research, Quality, and Policy in Homeless Health Care, Boston Health Care for the Homeless Program, Boston, Massachusetts; 4 Medical Practice Evaluation Center, Massachusetts General Hospital, Boston; 5 Department of Biostatistics, Boston University School of Public Health, Boston, Massachusetts; 6 Orthopedic and Arthritis Center for Outcomes Research, Department of Orthopedic Surgery, Brigham and Women’s Hospital, Boston, Massachusetts; 7 Policy and Innovation eValuation in Orthopedic Treatments Center, Department of Orthopedic Surgery, Brigham and Women’s Hospital, Boston, Massachusetts; 8 Section of General Internal Medicine, Boston University School of Medicine, Boston, Massachusetts; 9 Division of Infectious Diseases, Massachusetts General Hospital, Boston; 10 Division of General Academic Pediatrics, Department of Pediatrics, Massachusetts General Hospital, Boston; 11 Harvard University Center for AIDS Research, Boston, Massachusetts; 12 Division of Pulmonary and Critical Care Medicine, Massachusetts General Hospital, Boston; 13 Africa Health Research Institute, KwaZulu-Natal, South Africa; 14 Department of Epidemiology and Harvard Center for Population and Development Studies, Harvard T.H. Chan School of Public Health, Boston, Massachusetts; 15 Africa Health Research Institute, KwaZulu-Natal, South Africa; 16 Institute for Global Health, University College London, London, United Kingdom; 17 MRC/Wits Rural Public Health and Health Transitions Research Unit (Agincourt), University of Witwatersrand, Johannesburg, South Africa; 18 Department of Health Policy and Management, Harvard T. H. Chan School of Public Health, Boston, Massachusetts; 19 Department of Operations, Weatherhead School of Management, Case Western Reserve University, Cleveland, Ohio

## Abstract

**Importance:**

Approximately 356 000 people stay in homeless shelters nightly in the United States. They have high risk of contracting coronavirus disease 2019 (COVID-19).

**Objective:**

To assess the estimated clinical outcomes, costs, and cost-effectiveness associated with strategies for COVID-19 management among adults experiencing sheltered homelessness.

**Design, Setting, and Participants:**

This decision analytic model used a simulated cohort of 2258 adults residing in homeless shelters in Boston, Massachusetts. Cohort characteristics and costs were adapted from Boston Health Care for the Homeless Program. Disease progression, transmission, and outcomes data were taken from published literature and national databases. Surging, growing, and slowing epidemics (effective reproduction numbers [R_e_], 2.6, 1.3, and 0.9, respectively) were examined. Costs were from a health care sector perspective, and the time horizon was 4 months, from April to August 2020.

**Exposures:**

Daily symptom screening with polymerase chain reaction (PCR) testing of individuals with positive symptom screening results, universal PCR testing every 2 weeks, hospital-based COVID-19 care, alternative care sites (ACSs) for mild or moderate COVID-19, and temporary housing were each compared with no intervention.

**Main Outcomes and Measures:**

Cumulative infections and hospital-days, costs to the health care sector (US dollars), and cost-effectiveness, as incremental cost per case of COVID-19 prevented.

**Results:**

The simulated population of 2258 sheltered homeless adults had a mean (SD) age of 42.6 (9.04) years. Compared with no intervention, daily symptom screening with ACSs for pending tests or confirmed COVID-19 and mild or moderate disease was associated with 37% fewer infections (1954 vs 1239) and 46% lower costs ($6.10 million vs $3.27 million) at an R_e_ of 2.6, 75% fewer infections (538 vs 137) and 72% lower costs ($1.46 million vs $0.41 million) at an R_e_ of 1.3, and 51% fewer infections (174 vs 85) and 51% lower costs ($0.54 million vs $0.26 million) at an R_e_ of 0.9. Adding PCR testing every 2 weeks was associated with a further decrease in infections; incremental cost per case prevented was $1000 at an R_e _of 2.6, $27 000 at an R_e_ of 1.3, and $71 000 at an R_e_ of 0.9. Temporary housing with PCR every 2 weeks was most effective but substantially more expensive than other options. Compared with no intervention, temporary housing with PCR every 2 weeks was associated with 81% fewer infections (376) and 542% higher costs ($39.12 million) at an R_e_ of 2.6, 82% fewer infections (95) and 2568% higher costs ($38.97 million) at an R_e_ of 1.3, and 59% fewer infections (71) and 7114% higher costs ($38.94 million) at an R_e_ of 0.9. Results were sensitive to cost and sensitivity of PCR and ACS efficacy in preventing transmission.

**Conclusions and Relevance:**

In this modeling study of simulated adults living in homeless shelters, daily symptom screening and ACSs were associated with fewer severe acute respiratory syndrome coronavirus 2 (SARS-CoV-2) infections and decreased costs compared with no intervention. In a modeled surging epidemic, adding universal PCR testing every 2 weeks was associated with further decrease in SARS-CoV-2 infections at modest incremental cost and should be considered during future surges.

## Key Points

**Question:**

What are the projected clinical outcomes and costs associated with strategies for reducing severe acute respiratory syndrome coronavirus 2 infections among people experiencing sheltered homelessness?

**Findings:**

In this decision analytic model, daily symptom screening with polymerase chain reaction (PCR) testing of individuals who had positive symptom screening paired with nonhospital care site management of people with mild to moderate coronavirus disease 2019 (COVID-19) was associated with a substantial decrease in infections and lowered costs over 4 months compared with no intervention across a wide range of epidemic scenarios. In a surging epidemic, adding periodic universal PCR testing to symptom screening and nonhospital care site management was associated with improved clinical outcomes at modestly increased costs.

**Meaning:**

In this study, daily symptom screening with PCR testing of individuals who had positive symptom screening and use of alternative care sites for COVID-19 management among individuals experiencing sheltered homelessness were associated with substantially reduced new cases and costs compared with other strategies.

## Teaser

This decision analytic model assesses the clinical outcomes, costs, and cost-effectiveness associated with strategies for coronavirus disease 2019 (COVID-19) among US adults experiencing sheltered homelessness.

## Introduction

More than 1.4 million people experience sheltered homelessness annually in the United States, including approximately 356 000 each night.^1,2^ The crowded circumstances of homeless shelters place this population at increased risk of contracting coronavirus disease 2019 (COVID-19). The US Centers for Disease Control and Prevention (CDC) issued comprehensive guidance for preventing and mitigating COVID-19 among people experiencing sheltered homelessness, including recommendations for infection control practices in shelters, symptom screening of shelter guests, and dedicated settings for isolation and management of individuals with symptoms or confirmed illness.^3^ The high burden of COVID-19 among sheltered homeless populations^4,5,6,7^ highlights an urgent need to understand the clinical outcomes and costs of CDC-recommended and other prevention and treatment strategies. After a cluster of COVID-19 cases at a single large shelter in Boston, universal polymerase chain reaction (PCR) testing of 408 shelter residents found that 36% had severe acute respiratory syndrome coronavirus 2 (SARS-CoV-2) infection.^4^ Overall, 88% of these individuals reported no symptoms at the time of testing, raising questions about how to identify COVID-19 disease in this population and the role of nonhospital alternative care sites (ACSs) to isolate those who do not require hospitalization. The objective of this study was to project the clinical outcomes, costs, and cost-effectiveness associated with COVID-19 management approaches for adults experiencing sheltered homelessness.

## Methods

### Analytic Overview

We developed the Clinical and Economic Analysis of COVID-19 interventions (CEACOV) model, a dynamic microsimulation of the natural history of COVID-19 disease and the association of prevention, testing, and treatment interventions with outcomes and costs. We used CEACOV to project the clinical outcomes, costs, and cost-effectiveness of various COVID-19 management strategies for people experiencing sheltered homelessness, including different combinations of symptom screening, PCR testing, ACSs, and relocating all shelter residents to temporary housing. Using data from the early stage of an outbreak among adults experiencing homelessness in Boston, Massachusetts, we modeled a cohort of adults experiencing sheltered homelessness and examined management strategies under various epidemic scenarios, given evolving and heterogenous epidemic dynamics across the United States.^4,8^ We evaluated 3 scenarios over a 4-month time horizon, from April to August 2020, with different effective reproduction numbers (R_e_) representing surging (R_e_, 2.6), growing (R_e_, 1.3), and slowing (R_e_, 0.9) epidemics. Outcomes included number of infections, utilization of hospital and intensive care unit (ICU) beds, costs, and cost per COVID-19 case. The analysis was conducted from a health care sector perspective. This study was approved by the Partners Human Research Committee. This study followed the Consolidated Health Economic Evaluation Reporting Standards (CHEERS) reporting guideline.

### Model Structure

#### Disease States and Progression

CEACOV is a dynamic microsimulation model of COVID-19 based on a susceptible, exposed, infectious, recovered (SEIR) framework, including susceptible, exposed, infectious, recovered, and death states.^9^ Individuals with infection face daily probabilities of disease progression through 6 COVID-19 states: preinfectious latency, asymptomatic, mild or moderate disease, severe disease, critical disease, and recuperation. With mild or moderate disease, individuals have mild symptoms, such as cough or fever, that generally do not require inpatient management in a population with stable housing. With severe disease, symptoms warrant inpatient management. With critical disease, patients require ICU care. Recovered individuals cannot transmit and are assumed to be immune from repeated infection.^10^ eFigure 1 in the Supplement displays how patients moved through the model. We describe model validation in the eAppendix in the Supplement.

#### Transmission

Individuals with COVID-19 transmit to susceptible individuals at health state–stratified rates. We modeled a closed cohort, with transmissions occurring between people experiencing sheltered homelessness. All susceptible people face equal probabilities of contacting individuals with infection and becoming infected (homogenous mixing). The number of projected infections depends on COVID-19 prevalence, proportion of the population susceptible, transmission rates, and interventions that change contact rates or infectivity per contact. Transmission rates are calibrated to achieve the desired R_e_, which captures the mean number of transmissions per case. More details can be found in the eAppendix in the Supplement.

#### Testing and Care Interventions

Symptom screening or PCR tests are offered at intervals defined in each strategy; test sensitivities and specificities depend on COVID-19 health state. Care interventions include hospital care, ACSs, and temporary housing. Because adequate isolation for COVID-19 is not possible within congregate homeless shelters, care of individuals experiencing homelessness who have mild or moderate COVID-19 occurs either in hospitals or ACSs, such as large tents or nonhospital facilities with on-site medical staff.^11,12^ ACSs reduce transmission and hospital use for people with mild or moderate illness. Temporary housing reduces transmission by preemptively moving everyone from shelters to individual living units (eg, hotel or dormitory rooms) for the entire simulation period. Anyone who develops mild or moderate COVID-19 remains in temporary housing, which offers health monitoring and space for isolation but less intensive staffing and infection control than ACSs.

#### Resource Use, Costs, Cost-effectiveness, and Budget Impact

The model tallies resource utilization, including tests and days in hospital, ICU, ACS, or temporary housing, and daily costs, including medical supplies and personnel. We included a budget impact analysis to determine total costs over the 4-month simulation. To understand the tradeoffs between cost and infections prevented and highlight the relative return on investment for each strategy, we present efficiency frontiers, plotting number of infections prevented against total cost for each strategy.^13^ Because we focus on a cohort relevant to an individual city and because overall COVID-19 mortality is low, we report incremental cost per COVID-19 case prevented as an outcome; $1000/case prevented is approximately equivalent to $61 000/quality-adjusted life-year (QALY) gained at current case fatality levels.

### Strategies

We assessed 8 strategies, as follows:

1. No intervention: only basic infection control practices are implemented in shelters.
2. Symptom screening, PCR, and hospital: CDC-recommended symptom screening takes place daily in shelters.^14^ Individuals who screened negative remain in shelters. Individuals who screened positive are sent to the hospital for PCR testing. Individuals with positive PCR results remain in hospital; individuals with negative PCR results return to shelter.
3. Symptom screening, PCR, and ACS: CDC-recommended symptom screening takes place daily in shelters. Individuals who screened negative remain in shelters. Individuals who screened positive are sent to an ACS for people under investigation, where they undergo PCR testing and await results. Individuals with positive PCR results and mild or moderate illness are transferred to ACSs for confirmed COVID-19 cases. Individuals with negative PCR results return to shelter.
4. Universal PCR testing and hospital: universal PCR testing takes place every 2 weeks in shelters. Individuals with symptoms at the time of testing await results at the hospital; individuals without symptoms await results in shelters. Individuals with negative PCR results return to or stay in shelters. Individuals with positive PCR results, regardless of illness severity, remain in or are sent to the hospital.
5. Universal PCR and ACS: universal PCR testing takes place every 2 weeks in shelters. Those with symptoms at the time of testing are sent to an ACS for people under investigation while awaiting results; individuals without symptoms await results in shelters. Individuals with negative PCR results return to or stay in shelters. Individuals with positive PCR results and mild or moderate illness are transferred to ACSs for confirmed COVID-19 cases.
6. Universal PCR and temporary housing: all shelter residents are preemptively moved to temporary housing for the duration of the 4-month period. Universal PCR testing occurs every 2 weeks. Individuals with positive PCR results and mild or moderate illness remain in temporary housing and are transferred to the hospital if they progress to severe or critical disease.
7. Hybrid hospital: this includes the symptom screening, PCR, and hospital strategy and adds shelter-based universal PCR testing every 2 weeks for those without symptoms.
8. Hybrid ACS: this includes the symptom screening, PCR, and ACS strategy and adds shelter-based universal PCR testing every 2 weeks for those without symptoms.

In all 8 strategies, people with severe or critical illness are sent to the hospital. Individuals are eligible for repeated PCR testing 5 days after their most recent negative test (eFigure 2 in the Supplement).

### Input Parameters

#### Cohort Characteristics

The simulated cohort represents 2258 adults living in Boston homeless shelters.^2^ Overall, 1872 (83%) were aged 18 to 59 years, and 386 (17%) were aged 60 years or older (Table 1).^2,4,15,16,17,18,19,20,21,22,23,24,25,26,27,28,29,30,31,32,33,34,35,36,37^ The initial prevalence of active or past COVID-19 is assumed to be 2.2%. To reflect symptoms similar to but not due to COVID-19 (eg, from other respiratory viruses or seasonal rhinitis), susceptible and recovered individuals have a 0.01% daily probability of exhibiting mild or moderate COVID-19–like symptoms.^29,30,31^

**Table 1.** Input Parameters for an Analysis of Management Strategies for People Experiencing Sheltered Homelessness During the COVID-19 Pandemic

Parameter	Value	Source
**Cohort characteristics**		
Cohort size, No.	2258	Henry et al,^2^ 2020
Age distribution, No. (%)^a^		
18-59 y	1872 (82.9)	BHCHP
>60 y	386 (17.1)	
**Natural history**		
Probability of COVID-19 severity, stratified by age^b^		
18-59 y		Derived from MDPH,^15^ 2020; Mizumoto et al,^16^ 2020; Haridy,^17^ 2020; Li et al,^18^ 2020
Asymptomatic infection	0.262	
Mild or moderate illness	0.719	
Severe illness	0.012	
Critical illness	0.007	
>60 y		
Asymptomatic infection	0.180	
Mild or moderate illness	0.788	
Severe illness	0.001	
Critical illness	0.031	
Duration of illness state among hospitalized patients, stratified by COVID-19 severity, mean, d^c^		
Preinfectious latent to asymptomatic state		
Asymptomatic infection	2.6	Derived from WHO-China Joint Mission,^19^ 2020; Li et al,^20^ 2020; He et al,^21^ 2020; Linton et al,^22^ 2020
Mild or moderate illness	2.6	
Severe illness	2.6	
Critical illness	2.6	
Asymptomatic to mild or moderate state		
Asymptomatic infection	NA	WHO-China Joint Mission,^19^ 2020; He et al,^21^ 2020
Mild or moderate illness	2.0	
Severe illness	2.0	
Critical illness	2.0	
Mild or moderate to severe state		
Asymptomatic infection	NA	Wang et al,^23^ 2020
Mild or moderate illness	NA	
Severe illness	6.5	
Critical illness	3.0	
Severe to critical illness state		
Asymptomatic infection	NA	Zhou et al,^24^ 2020
Mild or moderate illness	NA	
Severe illness	10.5	
Critical illness	7.1	
Critical illness to recuperation state		
Asymptomatic infection	NA	Zhou et al,^24^ 2020
Mild or moderate illness	NA	
Severe illness	NA	
Critical illness	11.9	
Duration of illness state among nonhospitalized patients, stratified by COVID-19 severity, mean, d^c^		
Preinfectious latent to asymptomatic state		
Asymptomatic infection	2.6	Derived from WHO-China Joint Mission,^19^ 2020; Li et al,^20^ 2020; He et al,^21^ 2020; Linton et al,^22^ 2020
Mild or moderate illness	2.6	
Severe illness	2.6	
Critical illness	2.6	
Asymptomatic to mild or moderate state		
Asymptomatic infection	NA	WHO-China Joint Mission,^19^ 2020; He et al,^21^ 2020
Mild or moderate illness	2.0	
Severe illness	2.0	
Critical illness	2.0	
Mild or moderate to severe state		
Asymptomatic infection	NA	Wang et al,^23^ 2020
Mild or moderate illness	NA	
Severe illness	6.5	
Critical illness	3.0	
Severe to critical illness state		
Asymptomatic infection	NA	Zhou et al,^24^ 2020
Mild or moderate illness	NA	
Severe illness	NA	
Critical illness	6.5	
Duration of viral shedding, stratified by COVID-19 severity, mean, d^c^		
Asymptomatic infection	9.5	Zhou et al,^24^ 2020; WHO-China Joint Mission,^19^ 2020; Hu et al,^25^ 2020
Mild or moderate illness	12	
Severe illness	19	
Critical illness	24	
Daily probability of mortality in the critical state, stratified by age		
Hospital care		
18-59 y	0.004	Derived from Wang et al,^23^ 2020; Zhou et al,^24^ 2020
>60 y	0.050	
No hospital care		
18-59 y	0.166	Derived from MDPH,^15^ 2020; US Census Bureau,^26^ 2020; Richard et al,^27^ 2020
>60 y	0.203	
Daily probability of onward transmission, stratified by disease state		
Asymptomatic state	0.2394	Derived from Zhou et al,^24^ 2020; WHO-China Joint Mission,^19^ 2020; Hu et al,^25^ 2020; Liu et al,^28^ 2020
Mild or moderate state	0.1948	
Severe state	0.0135	
Critical state	0.0107	
Recuperation state	0.0135	
Persons with other respiratory illnesses exhibiting mild or moderate COVID-19–like symptoms, daily, %	0.01	Rui and Okeyode,^29^ 2019; CDC,^30^ 2020; CDC,^31^ 2020
Duration of mild or moderate COVID-19–like symptoms, mean, d	5	Assumed
Intervention		
Reduction in transmission rates, %^d^		
ACS for people with pending PCR test results	80	Assumed
ACS for people with confirmed COVID-19	100	Assumed
Temporary housing	60	Assumed
Hospitalization	100	Assumed
Intervention cost, 2020 US $		
ACS^a^		
Daily material cost	79	BHCHP
Daily personnel cost	225	
Total daily cost	304	
Temporary housing^a^		
Daily material cost	85	BHCHP
Daily personnel cost	56	
Total daily cost	141	
Hospital (non-ICU) bed^a^		
Daily material cost	NA	Derived from Cox et al,^32^ 2020; Rae et al,^33^ 2020; Fair Health,^34^ 2020
Daily personnel cost	NA	
Total daily cost	1641	
ICU bed		
Daily material cost	NA	Derived from Cox et al,^32^ 2020; Rae et al,^33^ 2020; Fair Health,^34^ 2020
Daily personnel cost	NA	
Total daily cost	2683	
**Testing**		
Symptom screening		
Sensitivity, stratified by disease state, %		
Preinfectious latent	0	Assumed
Asymptomatic state	0	Assumed
Mild or moderate state^e^	62	Derived from Baggett et al,^4^ 2020; assumed
Severe state	100	Assumed
Critical state	100	Assumed
Result return delay, d	0	Assumed
Unit cost, 2020 $	0	Assumed
PCR, nasopharyngeal specimen		
Sensitivity, stratified by disease state, %		
Pre-infectious latent	0	Assumed
Asymptomatic state	70	Assumed
Mild or moderate state	70	Yang et al,^35^ 2020; Wang et al,^36^ 2020
Severe state	100	Assumed
Critical state	100	Assumed
Specificity, %	100	Assumed
Result return delay, d	1	Assumed
Unit cost, 2020 $	51	CMS,^37^ 2020

Abbreviations: ACS, alternative care site; BHCHP, Boston Health Care for the Homeless Program; COVID-19, coronavirus disease 2019; ICU, intensive care unit; NA, not applicable; PCR, polymerase chain reaction; SARS-CoV-2, severe acute respiratory syndrome coronavirus 2.

^a^ Data on cohort characteristics and costs of alternative care sites and temporary housing were derived from unpublished data from the BHCHP.

^b^ Severity probability refers to the likelihood that an individual, once infected with SARS-CoV-2, will eventually progress to the specified severity of COVID-19 disease.

^c^ Durations of illness state and of viral shedding were derived from model inputs of transition probabilities (eTable 1 in the Supplement).

^d^ In ACSs for people with pending PCR test results, there are people without COVID-19 who are susceptible to infection. Transmission in ACSs for people with pending PCR test results is thus not completely reduced. In ACSs for people with confirmed COVID-19, complete reduction in transmission among individuals experiencing sheltered homelessness was assumed, and SARS-CoV-2 transmission to health care workers was not examined. Temporary housing is a less medicalized setting compared with hospitals and ACSs and was assumed to have a lower reduction in SARS-CoV-2 transmission rates.

^e^ The sensitivity of symptom screening for identifying individuals with mild to moderate COVID-19 was derived from an unpublished reanalysis of data from SARS-CoV-2 testing at a single large shelter in Boston, Massachusetts.^4^ Among individuals with COVID-19 individuals presenting with mild to moderate symptoms at time of testing, 15 of 18 (83%) would have been identified using a symptom screening instrument concordant with CDC guidelines.^14^ To account for the underreporting of symptoms among shelter residents due to stigma and/or fear of losing shelter accommodations, we estimated that only 75% of those with mild to moderate COVID-19 would report their symptoms. Thus, we estimated that the symptom screen would identify 62% (0.83 × 0.75) of shelter residents with mild to moderate COVID-19.

#### Progression of COVID-19 and Transmission

Mean duration of each COVID-19 state varies by severity (eTable 1 in the Supplement). The probabilities of developing severe or critical disease or dying increase with age.^23,24^ Transmission rates are highest for individuals in asymptomatic and mild or moderate states; individuals in severe and critical states have fewer infectious contacts because of hospitalization.^19,24,25,28^

#### Testing

We assumed symptom screen sensitivity of 0% for asymptomatic infection, 62% for mild or moderate COVID-19, and 100% for severe or critical COVID-19.^4^ The PCR test is a nasopharyngeal sample with a 1-day result delay, with 70% sensitivity for people with no symptoms or mild or moderate symptoms,^35,36^ 100% sensitivity for severe or critical illness, and 100% specificity.

#### Hospitalization, ACSs, and Temporary Housing

Mortality was decreased with hospitalization among those with critical illness.^23,24^ We assumed hospitalization reduces transmission by 100%, while ACSs reduce transmission by 80% and temporary housing by 60%. Temporary housing was assumed to be less effective at reducing transmission than ACSs because of less stringent infection control measures in temporary housing and potential mixing of individuals with and without infection. Length of stay at hospitals and ACSs depends on severity and duration of illness.^19,20,21,22,23,24,25,38^

#### Resource Use and Costs

The nasopharyngeal PCR test costs $51.^37^ Hospitalization costs $1641 per day; ICU costs $2683 per day (Table 1; eAppendix in the Supplement).^32,33,34^ ACSs cost $304 per day; temporary housing costs $141 per day (data from Boston Health Care for the Homeless Program [BHCHP]).

### Sensitivity Analyses

In 1-way sensitivity analyses, we examined the following: (1) PCR sensitivity, PCR frequency, and symptom screen sensitivity (eTable 2, eTable 3, and eTable 4 in the Supplement); (2) efficacy of ACS and temporary housing in reducing transmission (eTable 5 and eTable 6 in the Supplement); and (3) costs of PCR test, symptom screen, hospital care, ACS, and temporary housing (eTables 7-11 in the Supplement). In 2-way sensitivity analyses, we varied influential parameters simultaneously (eTable 12 and eTable 13 in the Supplement). To compare these findings with other settings, eTable 14 in the Supplement displays outcomes per 1000 adults experiencing homelessness and the number of adults experiencing sheltered homelessness in select US cities.

### Statistical Analysis

Due to the nature of our modeling study, no formal statistical testing was used, and we do not describe formal statistical significance. However, to reduce the effect of randomness and noise as well as to increase the precision in our results, we conducted 1 million individual simulations for each model run. Additionally, to evaluate the association of parameter uncertainty with our results, we conducted extensive univariate and multivariate sensitivity analyses.

## Results

### Base Case

#### Surging Epidemic

**Figure 1.**
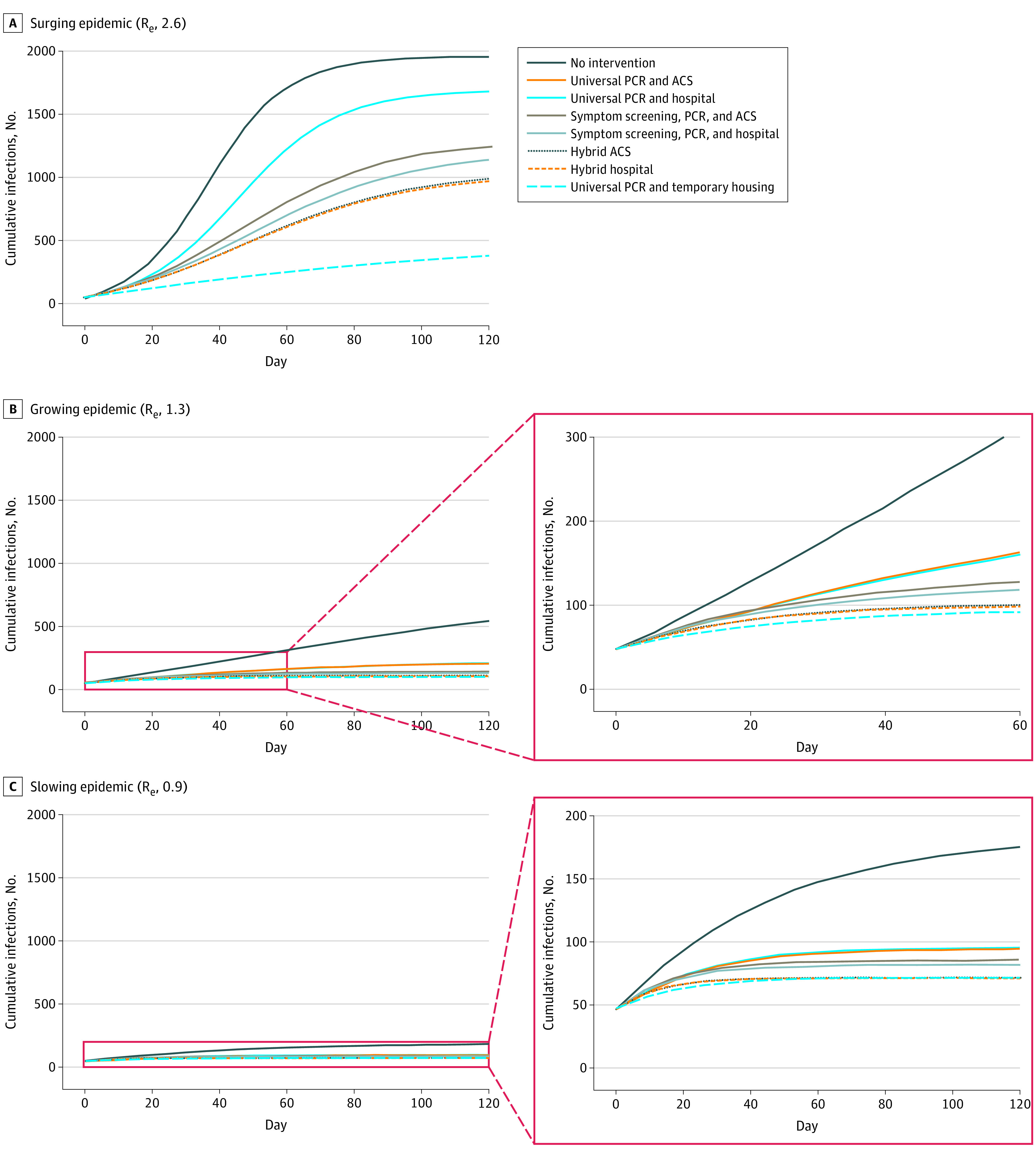
Cumulative Infections by Management Strategy for People Experiencing Sheltered Homelessness in Boston During the Coronavirus Disease 2019 Pandemic Over a 4-Month Period Day 0 on the horizontal axis represents the start of model simulation, with severe acute respiratory syndrome coronavirus 2 infection prevalence of 2.2%. The lines for the universal polymerase chain reaction (PCR) and hospital strategy and universal PCR and alternative care site (ACS) are overlapping lines because they differ only in costs; they are shown separately for clarity. The same is true for the hybrid hospital and hybrid ACS strategies. Strategy definitions appear in the Methods section.

The simulated population of 2258 sheltered homeless adults had a mean (SD) age of 42.6 (9.04) years. With an R_e_ of 2.6, the number of projected COVID-19 cases was highest with no intervention (1954) and lowest with the universal PCR and temporary housing strategy (376, an 81% reduction) (Table 2 and Figure 1).^15,39,40,41,42^ Other than the temporary housing strategy, strategies that relied on daily symptom screening were more effective in preventing infections (cumulative infections, 1133-1239) than those with universal PCR testing every 2 weeks alone (cumulative infections, 1679-1681). Daily symptom screening with ACSs for pending tests or confirmed COVID-19 and mild or moderate disease had 1239 infections, a 37% reduction from no intervention. Hybrid strategies involving daily symptom screening plus universal PCR testing every 2 weeks performed better than either strategy alone (cumulative infections, 967-985).

**Table 2.** Results of an Analysis of Management Strategies for 2258 People Experiencing Sheltered Homelessness During the Coronavirus Disease 2019 Pandemic at 4 Months

Strategy	Cumulative infections, No.	Reduction in cases, %^a^	Peak daily hospital bed use, No.	Total hospital days, No.	Total cost, 2020 $^b^	Cost compared with no intervention, 2020 $^b^	Incremental cost per case prevented, 2020 $^b^^,^^c^
R_e_, 2.6							
Symptom screening, PCR, and ACS	1239	36.6	5	394	3 267 000	−2 831 000	NA
Hybrid ACS	985	49.6	4	305	3 628 000	−2 470 000	1000
Universal PCR and ACS	1681	14.0	9	569	4 143 000	−1 955 000	Dominated
No intervention	1954	NA	64	3567	6 098 000	NA	Dominated
Hybrid hospital	967	50.5	80	6796	12 202 000	6 104 000	Dominated
Symptom screening, PCR, and hospital	1133	42.0	93	7656	12 620 000	6 522 000	Dominated
Universal PCR and hospital	1679	14.1	112	7165	12 914 000	6 816 000	Dominated
Universal PCR and temporary housing	376	80.8	1	121	39 119 000	33 021 000	58 000
R_e_, 1.3							
Symptom screening, PCR, and ACS	137	74.5	1	48	409 000	−1 052 000	NA
Hybrid ACS	103	80.8	1	69	1 325 000	−136 000	27 000
Universal PCR and ACS	207	61.5	1	34	1 426 000	−35 000	Dominated
No intervention	538	NA	9	867	1 461 000	NA	Dominated
Symptom screening, PCR, and hospital	125	76.7	22	966	1 604 000	143 000	Dominated
Hybrid hospital	100	81.4	23	815	2 368 000	907 000	382 000
Universal PCR and hospital	207	61.4	19	977	2 631 000	1 170 000	Dominated
Universal PCR and temporary housing	95	82.3	1	39	38 974 000	37 513 000	6 854 000
R_e_, 0.9							
Symptom screening, PCR, and ACS	85	51.2	1	30	264 000	−276 000	NA
No intervention	174	0.0	5	318	540 000	NA	Dominated
Symptom screening, PCR, and hospital	82	53.2	20	669	1 113 000	573 000	Dominated
Universal PCR and ACS	94	45.7	1	31	1 226 000	686 000	Dominated
Hybrid ACS	71	59.1	1	25	1 240 000	700 000	71 000
Universal PCR and hospital	95	45.5	19	534	1 901 000	1 361 000	Dominated
Hybrid hospital	71	59.4	22	595	2 004 000	1 464 000	Dominated
Universal PCR and temporary housing	71	59.2	1	29	38 954 000	38 414 000	Dominated

Abbreviations: ACS, alternative care site; NA, not applicable; PCR, polymerase chain reaction; R_e_, effective reproduction number.

^a^ Reduction in cases was calculated by dividing the number of cases prevented with the use of an alternative strategy by the number of cumulative cases for the no intervention strategy.

^b^ All costs are rounded to the nearest thousand.

^c^ Incremental costs per case prevented are calculated by dividing the difference in total costs by the difference in cumulative infections compared with the next most expensive strategy. All strategies are listed in order of ascending total costs, per convention of cost-effectiveness analysis. Using 9.50 years of life lost per COVID-19 death from the model and a mean age-stratified utility of 0.85 for the modeled population,^15,39,40,41^ a cost per case prevented of $1000 is equivalent to an incremental cost-effectiveness ratio of $61 000 per quality-adjusted life year gained. A ratio of $27 000 per case prevented is equivalent to $1 728 000 per quality-adjusted life-year gained. Any higher cost per case prevented has an even higher incremental cost-effectiveness ratio. Dominated indicates that a strategy is less clinically effective and more expensive than an alternative strategy or combination of 2 alternative strategies.^42^

**Figure 2.**
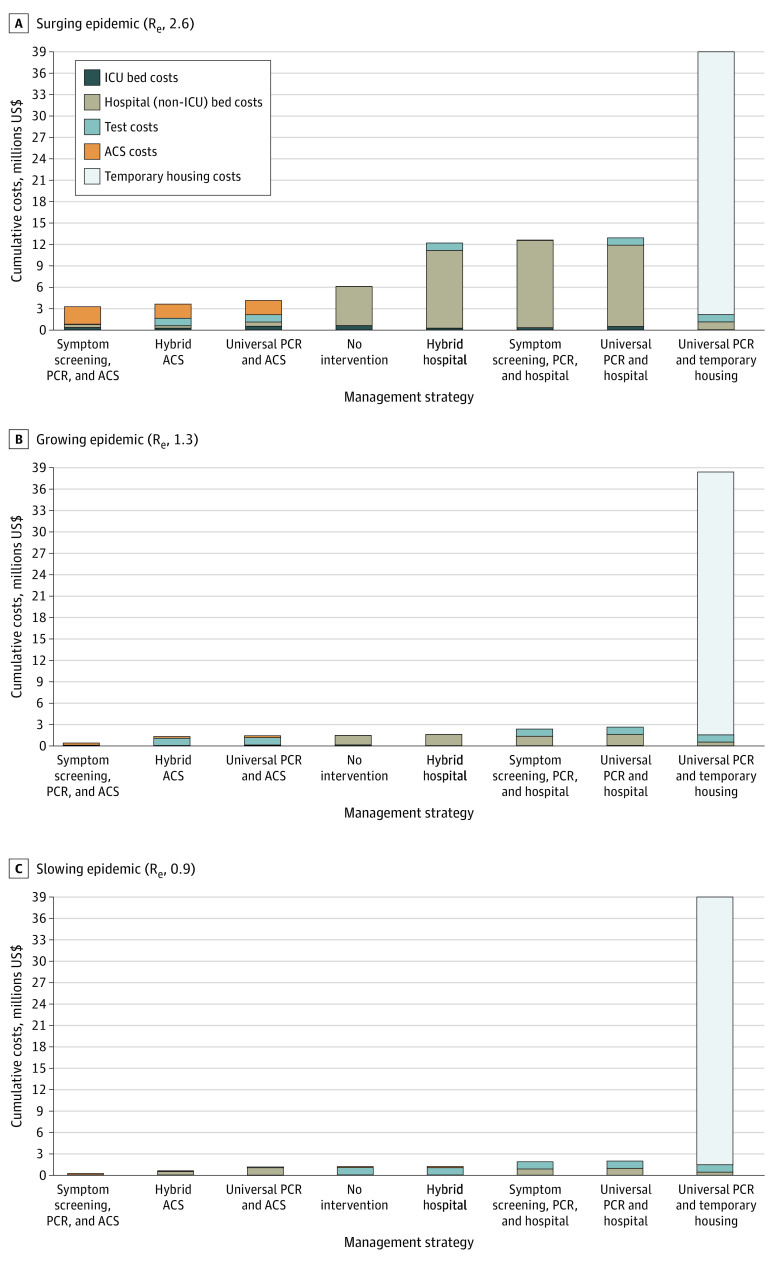
Health Care Sector Costs of Implementing Different Management Strategies for People Experiencing Sheltered Homelessness in Boston During the Coronavirus Disease 2019 Pandemic Over a 4-Month Period Costs are derived from model-generated results and are undiscounted. Strategy definitions appear in the Methods section. ACS indicates alternative care site; ICU, intensive care unit; PCR*,* polymerase chain reaction.

With an R_e_ of 2.6, all ACS-based strategies had lower total costs ($3.27-$4.14 million) than hospital-based strategies ($12.20-$12.91 million) and no intervention ($6.10 million) (Table 2 and Figure 2; eTable 15 in the Supplement). Daily symptom screening with ACSs for pending tests or confirmed COVID-19 and mild or moderate disease had 46% lower costs ($3.27 million). The universal PCR and temporary housing strategy was most expensive ($39.12 million), 542% greater than no intervention.

**Figure 3.**
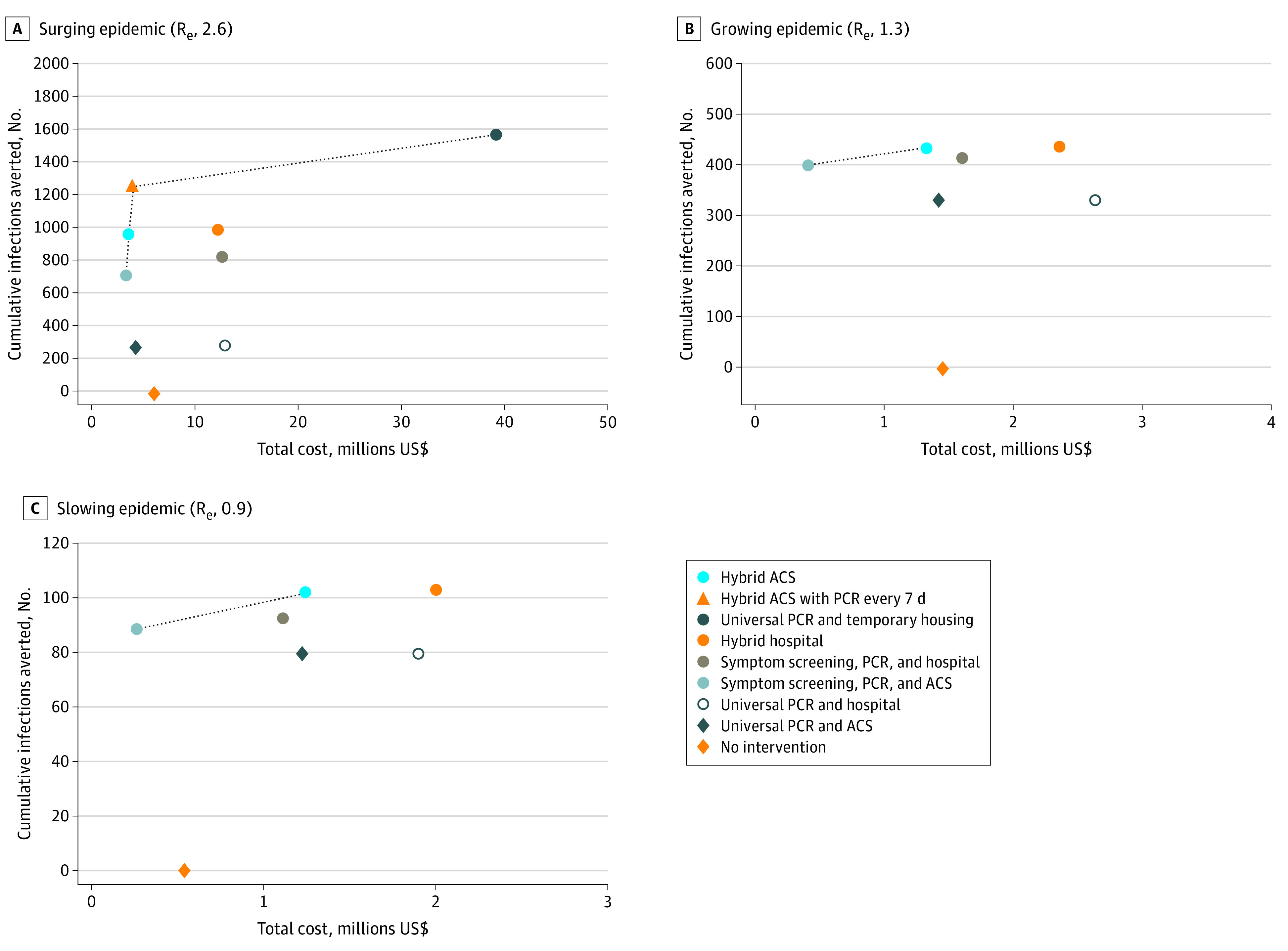
Infections Averted and Costs of Management Strategies for People Experiencing Sheltered Homelessness in Boston During the Coronavirus Disease 2019 Pandemic Over a 4-Month Period The dashed line represents the efficient frontier; strategies below this line are dominated ie, less clinically effective and more costly or with a higher incremental cost per case prevented than an alternative strategy or combination of strategies. Costs are from model-generated results and are undiscounted. Results for the universal polymerase chain reaction (PCR) and temporary housing strategy are not shown for R_e_ of 1.3 and 0.9. In addition to all base case strategies, Panel A also shows the hybrid alternative care site (ACS) strategy with PCR testing every 7 days. Strategy definitions appear in the Methods sections.

Compared with the symptom screening, PCR, and ACS strategy, the hybrid ACS strategy had 20% fewer cases (985 vs 1239) at $1000/case prevented (Table 2 and Figure 3A). The universal PCR and temporary housing strategy, the most clinically effective strategy, had an incremental cost of $58 000/case prevented compared with the hybrid ACS strategy. All other strategies were dominated, or less effective and more costly than another strategy or combination of strategies (Table 2 and Figure 3A; eTable 15 in the Supplement).

#### Growing Epidemic

With an R_e_ of 1.3, projected cases ranged from 538 (no intervention) to 95 (universal PCR with temporary housing, an 82% reduction) (Table 2 and Figure 1). All strategies had at least 60% fewer infections than no intervention. Strategies with ACS had fewer infections, fewer hospital days, and lower costs than no intervention, whereas hospital strategies had higher costs than no intervention (Table 2 and Figure 2; eTable 15 in the Supplement). The symptom screening, PCR, and ACS strategy had 75% fewer infections (358) than no intervention and the lowest cost ($0.41 million vs $1.46 million for no intervention, a 72% reduction). Compared with the symptom screening, PCR, and ACS strategy, the hybrid ACS strategy yielded an additional 6% decrease in infections at $27 000/case prevented. The universal PCR and temporary housing strategy had a cost of $38.97 million (a 2568% increase compared with no intervention) or $6 854 000/case prevented (Table 2 and Figure 3).

#### Slowing Epidemic

With an R_e_ of 0.9, cumulative infections were lower than in the other scenarios, ranging from 174 (no intervention) to 71 (universal PCR and temporary housing, a 59% reduction) (Table 2 and Figure 1). All strategies had at least 46% fewer infections than no intervention. The symptom screening, PCR, and ACS strategy had 51% fewer infections and 51% lower costs than no intervention (infections, 85 vs 174; cost, $0.26 million vs $0.54 million); it was the only strategy that cost less than no intervention (Table 2 and Figure 2; eTable 15 in the Supplement). Compared with the symptom screening, PCR, and ACS strategy, the hybrid ACS strategy yielded an additional 8% decrease in infections at $71 000/case prevented (Table 2 and Figure 3). Temporary housing with PCR every 2 weeks was associated with 7114% higher costs ($38.94 million) than no intervention.

### Sensitivity Analyses

#### One-Way Sensitivity Analysis

Across the 3 epidemic scenarios, changes in PCR sensitivity, PCR cost, PCR frequency, and ACS efficacy were associated with the greatest changes to incremental cost per case prevented. If PCR sensitivity increased from 70% to 90% with an R_e_ of 2.6, the number of infections with the hybrid ACS strategy decreased from 985 to 668; incremental cost per case prevented was $100 compared with the symptom screening, PCR, and ACS strategy (eTable 2 in the Supplement). If PCR cost decreased from $51 to $25 with an R_e_ of 2.6, the hybrid ACS strategy became cost-saving compared with the symptom screening, PCR, and ACS strategy (eTable 7 in the Supplement). Results for higher PCR costs are also shown in eTable 7 in the Supplement. If ACS efficacy in preventing transmissions decreased, total cases increased in all ACS-based strategies, and the hybrid ACS strategy became relatively less effective compared with symptom screening, PCR, and ACS (eTable 5 in the Supplement).

With an R_e_ of 2.6, the hybrid ACS strategy with universal PCR testing every 7 rather than every 14 days was associated with 29% fewer infections (incremental cost of $1000/case prevented compared with testing every 14 days) (Figure 3A; eTable 16 in the Supplement). Testing every 3 days had fewer infections, at $2000/case prevented. In other R_e_ scenarios, the hybrid ACS strategy did not result in a cost per case prevented below $20 000 compared with the symptom screening, PCR, and ACS strategy, regardless of universal testing frequency.

ACS-based management approaches remained less expensive than hospital care unless daily ACS costs began to approach hospital costs. Although the universal PCR with temporary housing strategy had the lowest number of cases in all scenarios, with an R_e_ of 2.6, daily costs of temporary housing needed to be $20 per day or less to have an incremental cost per case prevented of $1000 or less compared with the hybrid ACS strategy (eTable 11 in the Supplement). In the lower R_e_ scenarios, the universal PCR and temporary housing strategy had higher costs per case prevented.

#### Two-Way Sensitivity Analysis

In 2-way sensitivity analysis there were several combinations in which the hybrid ACS strategy was cost-saving or had an incremental cost per case prevented of $1000 to $3000 compared with the symptom screening, PCR, and ACS strategy. These results were associated with the sensitivity of PCR increasing and PCR cost decreasing (eTable 12 in the Supplement).

## Discussion

We developed a microsimulation model to examine the association of COVID-19 testing and isolation strategies with infections and health care costs among adults experiencing sheltered homelessness. Across all epidemic scenarios, daily symptom screening with PCR testing of individuals who had positive screening results and ACS-based COVID-19 management was the most efficient strategy and was cost-saving relative to no intervention.

In all cases, strategies using ACSs for isolation of symptomatic individuals with pending tests and for those with confirmed mild or moderate COVID-19, were associated with substantially decreased costs compared with analogous strategies relying on hospital-based care while achieving similar clinical outcomes. ACSs are especially useful for managing COVID-19 in sheltered homeless populations because people with mild to moderate illness cannot be effectively isolated in shelters. With high levels of SARS-CoV-2 infection among people experiencing homelessness in Boston and other cities,^4,5,6,7,43^ ACSs could avert many hospitalizations, preserving beds for individuals with severe illness and reducing costs. Boston created several such ACSs, ranging from 16-bed tents to a 500-bed field unit in a downtown convention center.^44^ In cities with smaller numbers of adults experiencing sheltered homelessness (eTable 14 in the Supplement), using existing facilities (eg, hotels or motels) as ACSs would avoid the fixed costs of new ACSs and allow for rapid implementation of care sites for people with mild to moderate COVID-19.

In a surging epidemic, adding universal PCR testing every 14 days to daily symptom screening had clinical benefits at an incremental cost of $1000 per case prevented. We selected a 2-week testing interval because this was deemed by BHCHP clinical staff to be realistic and in line with practice during the study time period; however, reducing the universal testing interval to every 7 days yielded additional benefits at $1000 per case prevented. In sensitivity analyses, this hybrid approach of daily symptom screening with additional periodic universal PCR testing was less expensive than daily symptom screening alone when PCR sensitivity increased and PCR cost decreased. In a growing or slowing epidemic, testing beyond daily symptom screening prevented a small number of new cases at high incremental costs. If PCR turnaround time were longer than the 1-day period we modeled, all strategies would have more cases and higher costs.

Temporary housing with universal PCR testing every 2 weeks was the most effective strategy for reducing COVID-19 in all scenarios but was also the most expensive, except in sensitivity analyses in which temporary housing costs were reduced below plausible ranges. However, this analysis does not account for other potential benefits of temporary housing on physical or mental health.^45^ Ultimately, broader policies around supportive housing measures for people experiencing homelessness should account for more than COVID-19 mitigation, recognizing that the COVID-19 pandemic is among many health risks of homelessness.^46^

This study complements the findings of a dynamic transition model of structural interventions for COVID-19 among people experiencing homelessness in England.^47^ In that analysis, single-room accommodations for people with COVID-19 symptoms and people without symptoms but at high risk of COVID-19 complications were projected to reduce infections, hospitalizations, and deaths by 36% to 64%. Our analysis adds to this by examining additional structural interventions (eg, ACSs and temporary housing) in a US context, combined with various COVID-19 diagnostic approaches (eg, symptom screening, universal PCR testing, and hybrid strategies) and by adding cost-effectiveness to inform policy and practice.

### Limitations

This analysis has limitations. The findings are specific to individual adults; we excluded adults experiencing homelessness as part of a family, because family shelters more likely provide private living quarters.^48^ We also excluded individuals experiencing unsheltered homelessness because disease transmission dynamics and infection control considerations are distinct for this subpopulation.^49^ We assumed homogeneous mixing of adults experiencing sheltered homelessness; in reality this population is spread over numerous shelters. This homogenous mixing assumption may affect the number of infections projected by our model, but we expect this to be small. In the base case, we did not assume increased comorbidities among adults experiencing homelessness compared with the general population.^50^ The analysis is based on the possibility that ACSs and PCR tests can be made available relatively quickly to this population. This may be difficult in some settings because those responsible for making ACSs and PCR tests available may not be those responsible for hospital costs, and record-keeping may be challenging. Finally, we focused this analysis on Boston, which has a 29.7% higher cost of living than the US mean.^51^ Costs of temporary housing may be considerably lower in other cities. However, in sensitivity analyses, results were robust to even large changes in testing, hospital, and housing costs.

## Conclusions

In this study, daily symptom screening and use of ACSs for those with pending test results or mild to moderate COVID-19 was associated with reduced infections and lower costs compared with no intervention. In a surging epidemic, adding universal PCR testing every 2 weeks was associated with further reduction in infections at a reasonable cost. Routine symptom screening, implementation of ACSs, and selective use of universal PCR testing should be implemented for sheltered homeless populations in the United States.

## Notes

Supplement. **eAppendix. **Supplemental Methods **eTable 1. **Additional Input Parameters for an Analysis of Management Strategies for People Experiencing Sheltered Homelessness During the COVID-19 Pandemic **eTable 2. **One-Way Sensitivity Analysis on PCR Sensitivity for People With Mild or Moderate Illness **eTable 3. **One-Way Sensitivity Analysis on Universal PCR Testing Frequency **eTable 4. **One-Way Sensitivity Analysis on Symptom Screen Sensitivity for People With Mild or Moderate Illness **eTable 5. **One-Way Sensitivity Analysis on Efficacy of ACS for Confirmed COVID-19 in Reducing SARS-CoV-2 Transmission **eTable 6. **One-Way Sensitivity Analysis on Efficacy of Temporary Housing in Reducing SARS-CoV-2 Transmission **eTable 7. **One-Way Sensitivity Analysis on Cost of a PCR Test **eTable 8. **One-Way Sensitivity Analysis on Cost of Symptom Screen **eTable 9. **One-Way Sensitivity Analysis on Daily Costs of Hospital Beds **eTable 10. **One-Way Sensitivity Analysis on Daily Cost of an ACS **eTable 11. **One-Way Sensitivity Analysis on Daily Cost of Temporary Housing **eTable 12. **Two-Way Sensitivity Analysis on PCR Sensitivity and PCR Cost for the Symptom Screening, PCR, and ACS and Hybrid ACS Strategies **eTable 13. **Two-Way Sensitivity Analysis on PCR Testing Frequency and PCR Cost for the Symptom Screening, PCR, and ACS and Hybrid ACS Strategies **eTable 14. **Results of an Analysis of Management Strategies for People Experiencing Sheltered Homelessness During the COVID-19 Pandemic for a Cohort of 1000 Adults Experiencing Sheltered Homelessness **eTable 15. **Total Infections and Component Costs of Different Management Strategies for Adults Experiencing Sheltered Homelessness in Boston During the COVID-19 Pandemic at 4 Months **eTable 16. **One-Way Sensitivity Analysis on PCR Testing Frequency for the Hybrid ACS Strategy **eFigure 1. **Illustration of Health States and Illness Paths in the CEACOV Model **eFigure 2. **Flow Diagrams of Management Strategies for People Experiencing Sheltered Homelessness in Boston During the COVID-19 Pandemic
