# Supplementary material for: Clinical Outcomes, Costs, and Cost-effectiveness of Strategies for Adults Experiencing Sheltered Homelessness During the COVID-19 Pandemic
Source: JAMA Netw Open. 2020 Dec 22;3(12):e2028195. doi: 10.1001/jamanetworkopen.2020.28195 (PMC7756240; doi:10.1001/jamanetworkopen.2020.28195)
Supplement: Supplement. — eAppendix. Supplemental Methods eTable 1. Additional Input Parameters for an Analysis of Management Strategies for People Experiencing Sheltered Homelessness During the COVID-19 Pandemic eTable 2. One-Way Sensitivity Analysis on PCR Sensitivity for People With Mild or Moderate Illness eTable 3. One-Way Sensitivity Analysis on Universal PCR Testing Frequency eTable 4. One-Way Sensitivity Analysis on Symptom Screen Sensitivity for People With Mild or Moderate Illness eTable 5. One-Way Sensitivity Analysis on Efficacy of ACS for Confirmed COVID-19 in Reducing SARS-CoV-2 Transmission eTable 6. One-Way Sensitivity Analysis on Efficacy of Temporary Housing in Reducing SARS-CoV-2 Transmission eTable 7. One-Way Sensitivity Analysis on Cost of a PCR Test eTable 8. One-Way Sensitivity Analysis on Cost of Symptom Screen eTable 9. One-Way Sensitivity Analysis on Daily Costs of Hospital Beds eTable 10. One-Way Sensitivity Analysis on Daily Cost of an ACS eTable 11. One-Way Sensitivity Analysis on Daily Cost of Temporary Housing eTable 12. Two-Way Sensitivity Analysis on PCR Sensitivity and PCR Cost for the Symptom Screening, PCR, and ACS and Hybrid ACS Strategies eTable 13. Two-Way Sensitivity Analysis on PCR Testing Frequency and PCR Cost for the Symptom Screening, PCR, and ACS and Hybrid ACS Strategies eTable 14. Results of an Analysis of Management Strategies for People Experiencing Sheltered Homelessness During the COVID-19 Pandemic for a Cohort of 1000 Adults Experiencing Sheltered Homelessness eTable 15. Total Infections and Component Costs of Different Management Strategies for Adults Experiencing Sheltered Homelessness in Boston During the COVID-19 Pandemic at 4 Months eTable 16. One-Way Sensitivity Analysis on PCR Testing Frequency for the Hybrid ACS Strategy eFigure 1. Illustration of Health States and Illness Paths in the CEACOV Model eFigure 2. Flow Diagrams of Management Strategies for People Experiencing Sheltered Homelessness in Boston During the COVID-19 Pandemic [file jamanetwopen-e2028195-s001.pdf]

Supplemental Online Content

Baggett TP, Scott JA, Le MH, et al. Clinical outcomes, costs, and cost-effectiveness of strategies for adults experiencing sheltered homelessness during the COVID-19 pandemic. *JAMA Netw Open*. 2020;3(12):e2028195.
doi:10.1001/jamanetworkopen.2020.28195

eAppendix. Supplemental Methods
eTable 1. Additional Input Parameters for an Analysis of Management Strategies for People Experiencing Sheltered Homelessness During the COVID-19 Pandemic
eTable 2. One-Way Sensitivity Analysis on PCR Sensitivity for People With Mild or Moderate Illness
eTable 3. One-Way Sensitivity Analysis on Universal PCR Testing Frequency
eTable 4. One-Way Sensitivity Analysis on Symptom Screen Sensitivity for People With Mild or Moderate Illness
eTable 5. One-Way Sensitivity Analysis on Efficacy of ACS for Confirmed COVID-19 in Reducing SARS-CoV-2 Transmission
eTable 6. One-Way Sensitivity Analysis on Efficacy of Temporary Housing in Reducing SARS-CoV-2 Transmission
eTable 7. One-Way Sensitivity Analysis on Cost of a PCR Test
eTable 8. One-Way Sensitivity Analysis on Cost of Symptom Screen
eTable 9. One-Way Sensitivity Analysis on Daily Costs of Hospital Beds
eTable 10. One-Way Sensitivity Analysis on Daily Cost of an ACS
eTable 11. One-Way Sensitivity Analysis on Daily Cost of Temporary Housing
eTable 12. Two-Way Sensitivity Analysis on PCR Sensitivity and PCR Cost for the Symptom Screening, PCR, and ACS and Hybrid ACS Strategies
eTable 13. Two-Way Sensitivity Analysis on PCR Testing Frequency and PCR Cost for the Symptom Screening, PCR, and ACS and Hybrid ACS Strategies
eTable 14. Results of an Analysis of Management Strategies for People Experiencing Sheltered Homelessness During the COVID-19 Pandemic for a Cohort of 1000 Adults Experiencing Sheltered Homelessness
eTable 15. Total Infections and Component Costs of Different Management Strategies for Adults Experiencing Sheltered Homelessness in Boston During the COVID-19 Pandemic at 4 Months
eTable 16. One-Way Sensitivity Analysis on PCR Testing Frequency for the Hybrid ACS Strategy
eFigure 1. Illustration of Health States and Illness Paths in the CEACOV Model
eFigure 2. Flow Diagrams of Management Strategies for People Experiencing Sheltered Homelessness in Boston During the COVID-19 Pandemic

© 2020 Baggett TP et al. *JAMA Network Open.*

This supplemental material has been provided by the authors to give readers additional information about their work.

© 2020 Baggett TP et al. *JAMA Network Open.*

eAppendix. Supplemental Methods

INTRODUCTION

In this supplemental appendix, we provide additional model inputs, results from one-way and multi-way sensitivity analyses, and scaled results for a cohort of 1,000 adults experiencing sheltered homelessness. In addition, we provide details on the Clinical and Economic Analysis of COVID-19 interventions (CEACOV) model and management strategies for people experiencing sheltered homelessness.

SUPPLEMENTAL METHODS

Health States

CEACOV simulates individuals transitioning between the states of susceptibility to severe acute respiratory syndrome coronavirus 2 (SARS-CoV-2), infection with SARS-CoV-2 and coronavirus disease 2019 (COVID-19) illness, recovery, or death. Susceptible individuals face a daily probability of acquiring infection. After acquiring SARS-CoV-2 infection, individuals may progress through the following health states (eFigure1):

- Pre-infectious latency
- Asymptomatic infection
- Mild/moderate illness: symptomatic
- Severe illness: dyspnea and/or hypoxemia ideally managed in a hospital with standard supplemental oxygen but not requiring intensive care unit (ICU)
- Critical illness: ideally managed in an ICU with high-flow supplemental oxygen, non-invasive positive pressure ventilation, or invasive mechanical ventilation
- Recuperation: for those recuperating from critical illness and improving while remaining in the hospital or other health care facility
- Recovered

Individuals in the asymptomatic infection, mild/moderate illness, and severe illness states can transition directly to the recovered state. Individuals in the critical illness state can eventually die, or transition to the recuperation state and then to the recovered state. The recovered state is an absorbing state, and recovered individuals are assumed to have immunity to SARS-CoV-2 over the model time horizon.

Natural History Paths

After being infected with SARS-CoV-2, a susceptible individual first transitions to the pre-infectious latency stage. Then, the individual has an age-dependent probability of progressing along one of four "paths," culminating in either asymptomatic infection, mild/moderate illness, severe illness, or critical illness. Before reaching a more advanced illness state, individuals first transition through intermediate states (e.g., those destined for severe illness must first pass through the asymptomatic infection state and the mild/moderate illness state, eFigure1).

Transmission

The basic reproduction number (R_0) is defined as the daily rate at which an infected individual contacts susceptible individuals and infects them in a fully susceptible cohort, multiplied by the duration of infectivity:

$R_0 = K * b * D$, where

- K: number of contacts per day an infected individual has with susceptible people in a fully susceptible cohort
- b: the probability of transmission per contact between an infectious individual and a susceptible person
- D: the mean duration of infectivity

In CEACOV, infected individuals in mild/moderate, severe, critical, and recuperation states can transmit SARS-CoV-2 to susceptible individuals. The effective transmission rate of infected individuals in one of these states is calculated as follows:

*Effective Transmission Rate (R_{eff}) = Nominal Transmission Rate (R_{nom}) * Transmission Multiplier.*

Nominal Transmission Rate = R_0/D.

© 2020 Baggett TP et al. *JAMA Network Open.*

The Nominal Transmission Rate is defined as R_0 in a fully susceptible cohort divided by the average duration of infectivity (D). Equivalently, it can be defined as a function of the average number of contacts per day an infected individual has with susceptible people in a fully susceptible cohort (K) multiplied by the probability of transmission per contact between an infectious individual and a susceptible person (b), therefore the contact rates and probability of transmission per contact are implicitly included in the nominal transmission rate. R_0 estimates the expected number of secondary cases produced by an infected individual in a fully susceptible cohort. Once the epidemic is underway and a subset of the population is infected, the effective reproduction number (R_e) measures the number of secondary cases produced by an infected individual in a progressed epidemic. This Nominal Transmission Rate captures the ratio (rather than the magnitude) of daily infectivity across different infection states (e.g., asymptomatic to mild/moderate, or mild/moderate to severe). Infected individuals do not transmit while they are in the latent state or in the recovered state. Patients in other infected states can transmit SARS-CoV-2 to susceptible individuals. The effective magnitude of the transmission rate changes over time as social interventions alter the number of contacts (K) and infectivity (b), and subsequently, the effective reproduction number (R_e); thus, the magnitude is adjusted using the transmission multiplier.Transmission multipliers are setting-specific, time-dependent adjusting factors, roughly accounting for population density and interventions that can alter the number of contacts (e.g., social distancing) and the infectivity per contact (e.g., masking) in the setting being modeled. In this analysis, the transmission multipliers were calibrated to represent a surging, a growing, and a slowing epidemic. Given the time-dependent uptake of such policies, time-varying transmission multipliers may be used to alter the effective transmission rate (R_{eff}) over time. R_{eff} determines the effective transmission rate of infected individuals on each day of simulation and is equivalent to the effective reproduction number (R_e) divided by the duration of infectivity. Therefore, the effective transmission rate (R_{eff}) in CEACOV and the effective reproduction number (R_e) are directly related.

In this analysis, we assumed that all susceptible persons have an equal probability of contacting infected individuals and acquiring the virus (i.e., homogenous mixing). As the epidemic grows, the number of susceptible persons declines. Thus, not all of the daily contacts of infected individuals will be with susceptible persons. The daily infection rate for a susceptible person is equal to the sum of transmission rates from all infected persons across all infection states divided by the cohort size. This leads to an expected daily number of infections equal to the number of susceptible people multiplied by the infection rate on that day.

It is worth noting that the number of secondary cases each infected individual produces is defined by the effective reproduction number (R_e). While the assumption on mixing (homogenous vs. heterogenous) affects 'who' an infected individual transmits to, the average number of new infections caused by a given infected individual is determined by the effective reproduction number.

Under the homogenous mixing assumption, an infected individual in Shelter A will uniformly infect susceptibles in other shelters; similarly, an infected individual in Shelter B will uniformly infect susceptibles in other shelters, including Shelter A. However, under the heterogeneous mixing assumption, an infected individual in Shelter A would most likely infect susceptibles in the same shelter, and an infected individual in Shelter B would similarly infect susceptibles in Shelter B. We assume that all shelters are roughly similar and that the assumption of homogenous mixing for this population is adequate.

Calibration and Validation of CEACOV

We initiated the model with a cohort of 1 million simulated persons who are meant to represent the 6.9 million population of Massachusetts (MA) in 2020. Each person's age category was drawn at model start based on MA age distribution data.[1] Prevalence of COVID-19 at model start was set to 0.14% to represent the approximate prevalence of COVID-19 in mid-March in MA.[2] We tracked the number of people in each health state over a 45-day horizon.

We calibrated the transmission multiplier (see above) to the COVID-19 epidemic in MA from mid-March to mid-April (first 30 days) and used the data from the remaining 15 days to validate the model. We assumed the reported number of COVID-19-attributable deaths would be close to the actual number of deaths. Hence, the number of reported COVID-19-attributable deaths was our main calibration target. We removed 59% of deaths to account for the deaths occurring in long term care facilities (LTCF) and not in the community. To ensure good model fit, we checked the mean absolute percentage error (MAPE) and the median absolute percentage error (MEDAPE) for

© 2020 Baggett TP et al. *JAMA Network Open.*

modeled and observed number of deaths over the validation horizon. The MAPE was 3.3%, while the MEDAPE was 3.2%.

Resource Costs

We did not include the cost of existing shelters, because this is not borne by the health care sector, or the cost of symptom screening, assuming this would be conducted by current shelter staff. Hospital costs were derived from IBM MarketScan Commercial Claims and Encounters database employer plans.[3,4] All other costs were in 2020 USD and unadjusted .

To estimate daily costs for hospital beds and intensive care unit (ICU) beds, we first used the average Medicare-allowed inpatient coverage of five diagnoses related to pneumonia and ventilator use as well as the average Medicare-allowed professional cost to obtain the total hospitalization costs. Subsequently, we divided the total costs by the average number of days that a patient with pneumonia spent in the hospital or the ICU to obtain the average daily costs for hospital beds and ICU beds, respectively.

More specifically, we used the average Medicare-allowed coverage across 3 diagnosis-related groups (DRG): DRG 193 "simple pneumonia and pleurisy with major complications", DRG 194 "pneumonia with complications or comorbidity," and DRG 195 "pneumonia without complications" to estimate the average Medicare-allowed inpatient coverage of pneumonia patients. In addition, we used DRG 207 "respiratory system diagnosis with ventilator support required for 96 hours or more", and DRG 208 "respiratory system diagnosis with ventilator support required for less than 96 hours" to estimate the average daily ICU cost. An average Medicare-allowed professional cost of $395 was added to the average allowed Medicare inpatient daily cost of hospitalization and ICU to obtain final total daily costs of hospitalization and ICU.[3–5]

CDC Symptom Screen Recommendations

The CDC recommends daily symptom screenings for COVID-19 at homeless shelters.[6] The following list of symptoms suggests the possibility of mild to moderate illness: fever, worsening cough, shortness of breath or difficulty breathing, fatigue, muscle or body aches, headache, new loss of taste or smell, sore throat, congestion or runny nose, nausea or vomiting, and diarrhea. More severe symptoms include trouble breathing, persistent pain or pressure in the chest, new confusion, inability to wake or stay awake, and bluish lips or face.[6]

© 2020 Baggett TP et al. *JAMA Network Open.*

APPENDIX REFERENCES

1. Census profile: Massachusetts. Census Reporter. Accessed July 29, 2020. http://censusreporter.org/profiles/04000US25-massachusetts/
2. Archive of COVID-19 cases in Massachusetts. Massachusetts Department of Public Health. Accessed July 29, 2020. https://www.mass.gov/info-details/archive-of-covid-19-cases-in-massachusetts
3. Rae M, Claxton G, Kurani N, McDermott D, Cox C. Potential costs of COVID-19 treatment for people with employer coverage. Peterson-Kaiser Health System Tracker. Published 2020. Accessed July 29, 2020. https://www.healthsystemtracker.org/brief/potential-costs-of-coronavirus-treatment-for-people-with-employer-coverage/
4. Cox C, Rudowitz R, Neuman T, Cubanski J, Rae M. How health costs might change with COVID-19. Health System Tracker. Published April 15, 2020. Accessed July 29, 2020. https://www.healthsystemtracker.org/brief/how-health-costs-might-change-with-covid-19/
5. *COVID-19: The Projected Economic Impact of the COVID-19 Pandemic on the US Healthcare System*. FAIR Health, Inc.; 2020. Accessed July 29, 2020. https://s3.amazonaws.com/media2.fairhealth.org/brief/asset/COVID-19%20-%20The%20Projected%20Economic%20Impact%20of%20the%20COVID-19%20Pandemic%20on%20the%20US%20Healthcare%20System.pdf
6. CDC. Screening clients for COVID-19 at homeless shelters or encampments. Centers for Disease Control and Prevention. Published May 20, 2020. Accessed July 29, 2020. https://www.cdc.gov/coronavirus/2019-ncov/community/homeless-shelters/screening-clients-respiratory-infection-symptoms.html
7. He X, Lau EH, Wu P, et al. Temporal dynamics in viral shedding and transmissibility of COVID-19. *medRxiv*. Published online January 1, 2020:2020.03.15.20036707. doi:10.1101/2020.03.15.20036707
8. Linton NM, Kobayashi T, Yang Y, et al. Incubation period and other epidemiological characteristics of 2019 novel coronavirus infections with right truncation: a statistical analysis of publicly available case data. *J Clin Med*. 2020;9(2):538. doi:10.3390/jcm9020538
9. *Report of the WHO-China Joint Mission on Coronavirus Disease 2019 (COVID-19)*. WHO-China Joint Mission on Coronavirus Disease 2019 (COVID-19); 2020. Accessed July 29, 2020. https://www.who.int/docs/default-source/coronaviruse/who-china-joint-mission-on-covid-19-final-report.pdf
10. Li R, Pei S, Chen B, et al. Substantial undocumented infection facilitates the rapid dissemination of novel coronavirus (SARS-CoV2). *Science*. Published online March 16, 2020:eabb3221. doi:10.1126/science.abb3221
11. Wang D, Hu B, Hu C, et al. Clinical characteristics of 138 hospitalized patients with 2019 novel coronavirus–infected pneumonia in Wuhan, China. *JAMA*. 2020;323(11):1061-1069. doi:10.1001/jama.2020.1585
12. Zhou F, Yu T, Du R, Fan G, Liu Y, Liu Z. Clinical course and risk factors for mortality of adult inpatients with COVID-19 in Wuhan, China: a retrospective cohort study. *The Lancet*. 2020;395(10229):1054-1062.
13. Hu Z, Song C, Xu C, et al. Clinical characteristics of 24 asymptomatic infections with COVID-19 screened among close contacts in Nanjing, China. *medRxiv*. Published online January 1, 2020:2020.02.20.20025619. doi:10.1101/2020.02.20.20025619
14. Sanders GD, Neumann PJ, Basu A, et al. Recommendations for conduct, methodological practices, and reporting of cost-effectiveness analyses: second panel on cost-effectiveness in health and medicine. *JAMA*. 2016;316(10):1093-1103. doi:10.1001/jama.2016.12195
15. Henry M, Watt R, Mahathey A, Ouellette J, Sitler A. *The 2019 Annual Homeless Assessment Report (AHAR) to Congress, Part 1: Point-in-Time Estimates of Homelessness*. The U.S. Department of Housing and Urban Development; 2020.

eTable 1. Additional Input Parameters for an Analysis of Management Strategies for People Experiencing Sheltered Homelessness During the COVID-19 Pandemic

**Parameter**	**Value**				**Source**
**Natural history**					
Initial illness distribution, %					Model calibration*
Susceptible	97.83				
Infected					
Pre-infectious latent	1.07				
Asymptomatic	0.56				
Mild/Moderate	0.35				
Severe	0.02				
Critical	0.00				
Recuperating	0.00				
Recovered	0.17				
Daily probability of transition between disease states, stratified by COVID-19 severity, among hospitalized	Asymptomatic infection	Mild/moderate illness	Severe illness	Critical illness	
Pre-infectious latency to asymptomatic state	0.323	0.323	0.323	0.323	Derived from [7–10]
Asymptomatic to mild/moderate state	NA	0.394	0.394	0.394	[7,9]
Mild/moderate to severe state	NA	NA	0.143	0.284	[11]
Severe to critical illness state	NA	NA	NA	0.105	[12]
Critical illness to recuperation state	NA	NA	NA	0.049	[12]
Daily probability of transition between disease states, stratified by COVID-19 severity, among non-hospitalized	Asymptomatic infection	Mild/moderate illness	Severe illness	Critical illness	
Pre-infectious latent to asymptomatic state	0.323	0.323	0.323	0.323	Derived from [7–10]
Asymptomatic to mild/moderate state	NA	0.394	0.394	0.394	[7,9]
Mild/moderate to severe state	NA	NA	0.143	0.284	[11]
Severe to critical illness state	NA	NA	NA	0.143	[12]

© 2020 Baggett TP et al. *JAMA Network Open.*

eTable 1 cont. Additional Input Parameters for an Analysis of Management Strategies for People Experiencing Sheltered Homelessness During the COVID-19 Pandemic

Parameter	Value				Source
**Natural history**					
Daily probability of transition to recovery, stratified by COVID-19 severity, among hospitalized	Asymptomatic infection	Mild/moderate illness	Severe illness	Critical illness	
Asymptomatic state to recovery	0.099	NA	NA	NA	Derived from [13]
Mild/moderate state to recovery	NA	0.095	NA	NA	Derived from [9]
Severe state to recovery	NA	NA	0.091	0.026	[12]
Recuperation to recovery	NA	NA	NA	0.161	[12]
Daily probability of transition to recovery, stratified by COVID-19 severity, among non-hospitalized	Asymptomatic infection	Mild/moderate illness	Severe illness	Critical illness	
Asymptomatic state to recovery	0.099	NA	NA	NA	Derived from [13]
Mild/moderate state to recovery	NA	0.095	NA	NA	Derived from [9]
Severe state to recovery	NA	NA	0.063	0.000	[12]
Recuperation to recovery	NA	NA	NA	0.000	

Abbreviations: Asymp., asymptomatic; COVID-19, coronavirus disease 2019; y, year.

*To obtain the initial illness distribution, we seeded the model with 0.01% of the starting cohort infected with the SARS-CoV-2 virus. We simulated the model for 10 days until the prevalence of SARS-CoV-2 in the cohort reached 2.2%. We used the illness distribution on Day 10 from this initialization run to inform our initial illness distribution in all model runs.

© 2020 Baggett TP et al. *JAMA Network Open.*

eTable 2. One-Way Sensitivity Analysis on PCR Sensitivity for People With Mild or Moderate Illness

Effective reproduction number (R_e)	PCR sensitivity for people with mild/mod. illness, %	Strategy	Cumulative infections, n	Total cost *, 2020 USD	Incr. cost per case prevented *, 2020 USD
2.6	70				
	base case	*SxScreen/PCR/ACS*	1,239	3,267,000	NA
		Hybrid/ACS	985	3,628,000	1,000
		UniversalPCR/ACS	1,681	4,143,000	Dominated
		NoIntervention	1,954	6,098,000	Dominated
		Hybrid/Hospital	967	12,202,000	Dominated
		SxScreen/PCR/Hospital	1,133	12,620,000	Dominated
		UniversalPCR/Hospital	1,679	12,914,000	Dominated
		UniversalPCR/TempHousing	376	39,119,000	58,000
	80				
		SxScreen/PCR/ACS	1,154	3,128,000	NA
		Hybrid/ACS	825	3,324,000	1,000
		UniversalPCR/ACS	1,616	4,214,000	Dominated
		NoIntervention	1,948	6,504,000	Dominated
		Hybrid/Hospital	809	10,854,000	Dominated
		SxScreen/PCR/Hospital	1,045	12,156,000	Dominated
		UniversalPCR/Hospital	1,613	13,451,000	Dominated
		UniversalPCR/TempHousing	380	39,756,000	82,000
	90				
		SxScreen/PCR/ACS	1,067	2,940,000	NA
		Hybrid/ACS	668	2,966,000	100
		UniversalPCR/ACS	1,546	4,222,000	Dominated
		NoIntervention	1,935	6,835,000	Dominated
		Hybrid/Hospital	658	9,377,000	Dominated
		SxScreen/PCR/Hospital	956	11,384,000	Dominated
		UniversalPCR/Hospital	1,548	13,824,000	Dominated
		UniversalPCR/TempHousing	365	39,766,000	122,000

© 2020 Baggett TP et al. *JAMA Network Open.*

eTable 2 cont. One-Way Sensitivity Analysis on PCR Sensitivity for People With Mild or Moderate Illness

Effective reproduction number (R_e)	PCR sensitivity for people with mild/mod. illness, %	Strategy	Cumulative infections, n	Total cost *, 2020 USD	Incr. cost per case prevented *, 2020 USD
2.6	100				
		Hybrid/ACS	512	2,556,000	NA
		SxScreen/PCR/ACS	994	2,777,000	Dominated
		UniversalPCR/ACS	1,473	4,188,000	Dominated
		NoIntervention	1,929	7,211,000	Dominated
		Hybrid/Hospital	514	7,834,000	Dominated
		SxScreen/PCR/Hospital	887	10,824,000	Dominated
		UniversalPCR/Hospital	1,478	13,897,000	Dominated
		UniversalPCR/TempHousing	375	39,760,000	Dominated
1.3	70				
	base case	*SxScreen/PCR/ACS*	137	409,000	NA
		Hybrid/ACS	103	1,325,000	27,000
		UniversalPCR/ACS	207	1,426,000	Dominated
		NoIntervention	538	1,461,000	Dominated
		SxScreen/PCR/Hospital	125	1,604,000	Dominated
		Hybrid/Hospital	100	2,368,000	382,000
		UniversalPCR/Hospital	207	2,631,000	Dominated
		UniversalPCR/TempHousing	95	38,974,000	6,854,000
	80				
		SxScreen/PCR/ACS	127	392,000	NA
		Hybrid/ACS	92	1,331,000	27,000
		UniversalPCR/ACS	177	1,414,000	Dominated
		NoIntervention	532	1,555,000	Dominated
		SxScreen/PCR/Hospital	118	1,588,000	Dominated
		Hybrid/Hospital	91	2,327,000	1,033,000
		UniversalPCR/Hospital	175	2,572,000	Dominated
		UniversalPCR/TempHousing	93	39,614,000	Dominated

© 2020 Baggett TP et al. *JAMA Network Open.*

eTable 2 cont. One-Way Sensitivity Analysis on PCR Sensitivity for People With Mild or Moderate Illness

Effective reproduction number (R_e)	PCR sensitivity for people with mild/mod. Illness, %	Strategy	Cumulative infections, n	Total cost *, 2020 USD	Incr. cost per case prevented *, 2020 USD
1.3	90				
		SxScreen/PCR/ACS	121	383,000	NA
		Hybrid/ACS	85	1,319,000	27,000
		UniversalPCR/ACS	148	1,384,000	Dominated
		SxScreen/PCR/Hospital	111	1,546,000	Dominated
		NoIntervention	518	1,599,000	Dominated
		Hybrid/Hospital	84	2,292,000	816,000
		UniversalPCR/Hospital	147	2,486,000	Dominated
		UniversalPCR/TempHousing	93	39,619,000	Dominated
	100				
		SxScreen/PCR/ACS	114	377,000	NA
		Hybrid/ACS	79	1,307,000	26,000
		UniversalPCR/ACS	134	1,373,000	Dominated
		SxScreen/PCR/Hospital	109	1,545,000	Dominated
		NoIntervention	498	1,645,000	Dominated
		Hybrid/Hospital	79	2,258,000	Dominated
		UniversalPCR/Hospital	131	2,444,000	Dominated
		UniversalPCR/TempHousing	93	39,613,000	Dominated
0.9	70				
	base case	*SxScreen/PCR/ACS*	85	264,000	NA
		NoIntervention	174	540,000	Dominated
		SxScreen/PCR/Hospital	82	1,113,000	Dominated
		UniversalPCR/ACS	94	1,226,000	Dominated
		Hybrid/ACS	71	1,240,000	71,000
		UniversalPCR/Hospital	95	1,901,000	Dominated
		Hybrid/Hospital	71	2,004,000	Dominated
		UniversalPCR/TempHousing	71	38,954,000	Dominated

© 2020 Baggett TP et al. *JAMA Network Open.*

eTable 2 cont. One-Way Sensitivity Analysis on PCR Sensitivity for People With Mild or Moderate Illness

Effective reproduction number (R_e)	PCR sensitivity for people with mild/mod. illness, %	Strategy	Cumulative infections, n	Total cost *, 2020 USD	Incr. cost per case prevented *, 2020 USD
0.9	80				
		SxScreen/PCR/ACS	82	259,000	NA
		NoIntervention	171	569,000	Dominated
		SxScreen/PCR/Hospital	78	1,109,000	Dominated
		UniversalPCR/ACS	87	1,252,000	Dominated
		Hybrid/ACS	68	1,257,000	74,000
		UniversalPCR/Hospital	87	1,944,000	Dominated
		Hybrid/Hospital	67	2,001,000	677,000
		UniversalPCR/TempHousing	72	39,601,000	Dominated
	90				
		SxScreen/PCR/ACS	80	263,000	NA
		NoIntervention	169	612,000	Dominated
		SxScreen/PCR/Hospital	76	1,105,000	Dominated
		Hybrid/ACS	65	1,252,000	67,000
		UniversalPCR/ACS	83	1,253,000	Dominated
		UniversalPCR/Hospital	82	1,973,000	Dominated
		Hybrid/Hospital	64	2,024,000	1,036,000
		UniversalPCR/TempHousing	72	39,604,000	Dominated
	100				
		SxScreen/PCR/ACS	78	269,000	NA
		NoIntervention	164	628,000	Dominated
		SxScreen/PCR/Hospital	74	1,123,000	Dominated
		UniversalPCR/ACS	76	1,249,000	Dominated
		Hybrid/ACS	63	1,254,000	65,000
		UniversalPCR/Hospital	77	1,973,000	Dominated
		Hybrid/Hospital	62	2,035,000	3,641,000
		UniversalPCR/TempHousing	71	39,596,000	Dominated

Abbreviations: ACS, alternate care sites; COVID-19, coronavirus disease 2019; Dominated, less clinically effective and more costly than an alternative strategy, or a combination of two alternative strategies;[14] Incr., incremental; mild/mod., mild/moderate; *PCR,* polymerase chain reaction; *SxScreen,* symptom screen; *TempHousing,* temporary housing; *UniversalPCR*, universal polymerase chain reaction test for everyone; USD, United States dollars.

© 2020 Baggett TP et al. *JAMA Network Open.*

Strategies are listed in order of ascending costs, per convention of cost-effectiveness analysis.

* Costs were rounded to the nearest thousands.

© 2020 Baggett TP et al. *JAMA Network Open.*

eTable 3. One-Way Sensitivity Analysis on Universal PCR Testing Frequency

Effective reproduction number (R_e)	Universal PCR testing frequency	Strategy	Cumulative infections, n	Total cost *, 2020 USD	Incr. cost per case prevented *, 2020 USD
2.6	Every 30 days				
		UniversalPCR/ACS	1,880	3,131,000	NA
		SxScreen/PCR/ACS	1,244	3,295,000	300
		Hybrid/ACS	1,179	3,594,000	5,000
		NoIntervention	1,954	6,114,000	Dominated
		UniversalPCR/Hospital	1,876	8,912,000	Dominated
		SxScreen/PCR/Hospital	1,138	12,738,000	Dominated
		Hybrid/Hospital	1,165	13,224,000	Dominated
		UniversalPCR/TempHousing	374	39,302,000	44,000
	Every 14 days				
	base case	*SxScreen/PCR/ACS*	1,239	3,267,000	NA
		Hybrid/ACS	985	3,628,000	1,000
		UniversalPCR/ACS	1,681	4,143,000	Dominated
		NoIntervention	1,954	6,098,000	Dominated
		Hybrid/Hospital	967	12,202,000	Dominated
		SxScreen/PCR/Hospital	1,133	12,620,000	Dominated
		UniversalPCR/Hospital	1,679	12,914,000	Dominated
		UniversalPCR/TempHousing	376	39,119,000	58,000
	Every 7 days				
		SxScreen/PCR/ACS	1,244	3,295,000	NA
		Hybrid/ACS	700	3,818,000	Dominated
		UniversalPCR/ACS	1,278	4,862,000	Dominated
		NoIntervention	1,954	6,114,000	Dominated
		Hybrid/Hospital	679	10,340,000	Dominated
		SxScreen/PCR/Hospital	1,138	12,738,000	Dominated
		UniversalPCR/Hospital	1,278	14,554,000	Dominated
		UniversalPCR/TempHousing	375	40,555,000	43,000

© 2020 Baggett TP et al. *JAMA Network Open.*

eTable 3 cont. One-Way Sensitivity Analysis on Universal PCR Testing Frequency

Effective reproduction number (R_e)	Universal PCR testing frequency	Strategy	Cumulative infections, n	Total cost *, 2020 USD	Incr. cost per case prevented *, 2020 USD
2.6	Every 3 days				
		SxScreen/PCR/ACS	1,244	3,295,000	NA
		Hybrid/ACS	334	4,609,000	1,000
		UniversalPCR/ACS	444	4,872,000	Dominated
		NoIntervention	1,954	6,114,000	Dominated
		Hybrid/Hospital	317	8,152,000	Dominated
		UniversalPCR/Hospital	437	9,455,000	Dominated
		SxScreen/PCR/Hospital	1,138	12,738,000	Dominated
		UniversalPCR/TempHousing	376	42,288,000	Dominated
1.3	Every 30 days				
		SxScreen/PCR/ACS	137	409,000	NA
		Hybrid/ACS	115	896,000	22,000
		UniversalPCR/ACS	338	1,008,000	Dominated
		NoIntervention	544	1,485,000	Dominated
		SxScreen/PCR/Hospital	125	1,608,000	Dominated
		Hybrid/Hospital	112	1,966,000	312,000
		UniversalPCR/Hospital	345	2,136,000	Dominated
		UniversalPCR/TempHousing	95	39,145,000	2,191,000
	Every 14 days				
	base case	*SxScreen/PCR/ACS*	137	409,000	NA
		Hybrid/ACS	103	1,325,000	27,000
		UniversalPCR/ACS	207	1,426,000	Dominated
		NoIntervention	538	1,461,000	Dominated
		SxScreen/PCR/Hospital	125	1,604,000	Dominated
		Hybrid/Hospital	100	2,368,000	382,000
		UniversalPCR/Hospital	207	2,631,000	Dominated
		UniversalPCR/TempHousing	95	38,974,000	6,854,000

© 2020 Baggett TP et al. *JAMA Network Open.*

eTable 3 cont. One-Way Sensitivity Analysis on Universal PCR Testing Frequency

**Effective reproduction number (R_e)**	**Universal PCR testing frequency**	**Strategy**	**Cumulative infections, n**	**Total cost *, 2020 USD**	**Incr. cost per case prevented *, 2020 USD**
1.3	Every 7 days				
		SxScreen/PCR/ACS	137	409,000	NA
		NoIntervention	544	1,485,000	Dominated
		SxScreen/PCR/Hospital	125	1,608,000	Dominated
		Hybrid/ACS	91	2,123,000	37,000
		UniversalPCR/ACS	124	2,162,000	Dominated
		Hybrid/Hospital	90	3,122,000	739,000
		UniversalPCR/Hospital	120	3,217,000	Dominated
		UniversalPCR/TempHousing	91	40,423,000	Dominated
	Every 3 days				
		SxScreen/PCR/ACS	137	409,000	NA
		NoIntervention	544	1,485,000	Dominated
		SxScreen/PCR/Hospital	125	1,608,000	Dominated
		UniversalPCR/ACS	83	3,815,000	Dominated
		Hybrid/ACS	79	3,817,000	59,000
		UniversalPCR/Hospital	82	4,757,000	Dominated
		Hybrid/Hospital	78	4,780,000	1,119,000
		UniversalPCR/TempHousing	95	42,141,000	Dominated
0.9	Every 30 days				
		SxScreen/PCR/ACS	85	264,000	NA
		NoIntervention	175	543,000	Dominated
		UniversalPCR/ACS	118	768,000	Dominated
		Hybrid/ACS	74	785,000	46,000
		SxScreen/PCR/Hospital	82	1,115,000	Dominated
		UniversalPCR/Hospital	121	1,349,000	Dominated
		Hybrid/Hospital	75	1,555,000	Dominated
		UniversalPCR/TempHousing	72	39,131,000	21,790,000

© 2020 Baggett TP et al. *JAMA Network Open.*

eTable 3 cont. One-Way Sensitivity Analysis on Universal PCR Testing Frequency

Effective reproduction number (R_e)	Universal PCR testing frequency	Strategy	Cumulative infections, n	Total cost *, 2020 USD	Incr. cost per case prevented *, 2020 USD
0.9	Every 14 days				
	base case	*SxScreen/PCR/ACS*	85	264,000	NA
		NoIntervention	174	540,000	Dominated
		SxScreen/PCR/Hospital	82	1,113,000	Dominated
		UniversalPCR/ACS	94	1,226,000	Dominated
		Hybrid/ACS	71	1,240,000	71,000
		UniversalPCR/Hospital	95	1,901,000	Dominated
		Hybrid/Hospital	71	2,004,000	Dominated
		UniversalPCR/TempHousing	71	38,954,000	Dominated
	Every 7 days				
		SxScreen/PCR/ACS	85	264,000	NA
		NoIntervention	175	543,000	Dominated
		SxScreen/PCR/Hospital	82	1,115,000	Dominated
		UniversalPCR/ACS	78	2,049,000	Dominated
		Hybrid/ACS	68	2,057,000	103,000
		UniversalPCR/Hospital	76	2,776,000	Dominated
		Hybrid/Hospital	67	2,810,000	Dominated
		UniversalPCR/TempHousing	71	40,407,000	Dominated
	Every 3 days				
		SxScreen/PCR/ACS	85	264,000	NA
		NoIntervention	175	543,000	Dominated
		SxScreen/PCR/Hospital	82	1,115,000	Dominated
		Hybrid/ACS	64	3,763,000	164,000
		UniversalPCR/ACS	65	3,764,000	Dominated
		UniversalPCR/Hospital	65	4,527,000	Dominated
		Hybrid/Hospital	64	4,553,000	Dominated
		UniversalPCR/TempHousing	71	42,126,000	Dominated

Abbreviations: ACS, alternate care sites; COVID-19, coronavirus disease 2019; Dominated, less clinically effective and more costly than an alternative strategy, or a combination of two alternative strategies;[14] Incr., incremental; *PCR,* polymerase chain reaction; *SxScreen,* symptom screen; *TempHousing,* temporary housing; *UniversalPCR*, universal polymerase chain reaction test for everyone; USD, United States dollars.

© 2020 Baggett TP et al. *JAMA Network Open.*

Strategies are listed in order of ascending costs, per convention of cost-effectiveness analysis.

* Costs were rounded to the nearest thousands.

© 2020 Baggett TP et al. *JAMA Network Open.*

eTable 4. One-Way Sensitivity Analysis on Symptom Screen Sensitivity for People With Mild or Moderate Illness

Effective reproduction number (R_e)	Sx screen sensitivity for people with mild/mod. illness, %	Strategy	Cumulative infections, n	Total cost *, 2020 USD	Incr. cost per case prevented *, 2020 USD
2.6	50				
		SxScreen/PCR/ACS	1,306	3,376,000	NA
		Hybrid/ACS	1,037	3,784,000	2,000
		UniversalPCR/ACS	1,681	4,143,000	Dominated
		NoIntervention	1,954	6,098,000	Dominated
		Hybrid/Hospital	1,015	12,598,000	Dominated
		UniversalPCR/Hospital	1,679	12,914,000	Dominated
		SxScreen/PCR/Hospital	1,214	13,313,000	Dominated
		UniversalPCR/TempHousing	376	39,119,000	53,000
	62				
	base case	*SxScreen/PCR/ACS*	1,239	3,267,000	NA
		Hybrid/ACS	985	3,628,000	1,000
		UniversalPCR/ACS	1,681	4,143,000	Dominated
		NoIntervention	1,954	6,098,000	Dominated
		Hybrid/Hospital	967	12,202,000	Dominated
		SxScreen/PCR/Hospital	1,133	12,620,000	Dominated
		UniversalPCR/Hospital	1,679	12,914,000	Dominated
		UniversalPCR/TempHousing	376	39,119,000	58,000
	70				
		SxScreen/PCR/ACS	1,210	3,240,000	NA
		Hybrid/ACS	965	3,634,000	2,000
		UniversalPCR/ACS	1,681	4,143,000	Dominated
		NoIntervention	1,954	6,098,000	Dominated
		Hybrid/Hospital	933	11,919,000	Dominated
		SxScreen/PCR/Hospital	1,103	12,556,000	Dominated
		UniversalPCR/Hospital	1,679	12,914,000	Dominated
		UniversalPCR/TempHousing	376	39,119,000	60,000

© 2020 Baggett TP et al. *JAMA Network Open.*

eTable 4 cont. One-Way Sensitivity Analysis on Symptom Screen Sensitivity for People With Mild or Moderate Illness

Effective reproduction number (R_e)	Sx screen sensitivity for people with mild/mod. illness, %	Strategy	Cumulative infections, n	Total cost *, 2020 USD	Incr. cost per case prevented *, 2020 USD
2.6	80				
		SxScreen/PCR/ACS	1,181	3,167,000	NA
		Hybrid/ACS	940	3,593,000	2,000
		UniversalPCR/ACS	1,681	4,143,000	Dominated
		NoIntervention	1,954	6,098,000	Dominated
		Hybrid/Hospital	909	11,785,000	Dominated
		SxScreen/PCR/Hospital	1,062	12,257,000	Dominated
		UniversalPCR/Hospital	1,680	12,986,000	Dominated
		UniversalPCR/TempHousing	376	39,119,000	63,000
1.3	50				
		SxScreen/PCR/ACS	147	426,000	NA
		Hybrid/ACS	105	1,349,000	22,000
		UniversalPCR/ACS	207	1,426,000	Dominated
		NoIntervention	538	1,461,000	Dominated
		SxScreen/PCR/Hospital	134	1,659,000	Dominated
		Hybrid/Hospital	102	2,384,000	345,000
		UniversalPCR/Hospital	207	2,631,000	Dominated
		UniversalPCR/TempHousing	95	38,974,000	5,254,000
	62				
	base case	*SxScreen/PCR/ACS*	137	409,000	NA
		Hybrid/ACS	103	1,325,000	27,000
		UniversalPCR/ACS	207	1,426,000	Dominated
		NoIntervention	538	1,461,000	Dominated
		SxScreen/PCR/Hospital	125	1,604,000	Dominated
		Hybrid/Hospital	100	2,368,000	348,000
		UniversalPCR/Hospital	207	2,631,000	Dominated
		UniversalPCR/TempHousing	95	38,974,000	6,854,000

© 2020 Baggett TP et al. *JAMA Network Open.*

eTable 4 cont. One-Way Sensitivity Analysis on Symptom Screen Sensitivity for People With Mild or Moderate Illness

Effective reproduction number (R_e)	Sx screen sensitivity for people with mild/mod. illness, %	Strategy	Cumulative infections, n	Total cost *, 2020 USD	Incr. cost per case prevented *, 2020 USD
1.3	70				
		SxScreen/PCR/ACS	134	403,000	NA
		Hybrid/ACS	103	1,346,000	30,000
		UniversalPCR/ACS	207	1,426,000	Dominated
		NoIntervention	538	1,461,000	Dominated
		SxScreen/PCR/Hospital	120	1,589,000	Dominated
		Hybrid/Hospital	99	2,365,000	255,000
		UniversalPCR/Hospital	207	2,631,000	Dominated
		UniversalPCR/TempHousing	95	38,974,000	9,735,000
	80				
		SxScreen/PCR/ACS	130	399,000	NA
		Hybrid/ACS	101	1,343,000	33,000
		UniversalPCR/ACS	207	1,426,000	Dominated
		NoIntervention	538	1,461,000	Dominated
		SxScreen/PCR/Hospital	120	1,598,000	Dominated
		Hybrid/Hospital	97	2,359,000	254,000
		UniversalPCR/Hospital	207	2,631,000	Dominated
		UniversalPCR/TempHousing	95	39,974,000	16,390,000
0.9	50				
		SxScreen/PCR/ACS	86	265,000	NA
		NoIntervention	174	540,000	Dominated
		SxScreen/PCR/Hospital	83	1,090,000	Dominated
		UniversalPCR/ACS	94	1,226,000	Dominated
		Hybrid/ACS	73	1,258,000	76,000
		UniversalPCR/Hospital	95	1,901,000	Dominated
		Hybrid/Hospital	72	2,007,000	749,000
		UniversalPCR/TempHousing	71	38,954,000	58,807,000

© 2020 Baggett TP et al. *JAMA Network Open.*

eTable 4 cont. One-Way Sensitivity Analysis on Symptom Screen Sensitivity for People With Mild or Moderate Illness

Effective reproduction number (R_e)	Sx screen sensitivity for people with mild/mod. illness, %	Strategy	Cumulative infections, n	Total cost *, 2020 USD	Incr. cost per case prevented *, 2020 USD
0.9	62				
	base case	*SxScreen/PCR/ACS*	85	264,000	NA
		NoIntervention	174	540,000	Dominated
		SxScreen/PCR/Hospital	82	1,113,000	Dominated
		UniversalPCR/ACS	94	1,226,000	Dominated
		Hybrid/ACS	71	1,240,000	70,000
		UniversalPCR/Hospital	95	1,901,000	Dominated
		Hybrid/Hospital	71	2,004,000	Dominated
		UniversalPCR/TempHousing	71	38,954,000	Dominated
	70				
		SxScreen/PCR/ACS	82	262,000	NA
		NoIntervention	174	540,000	Dominated
		SxScreen/PCR/Hospital	80	1,101,000	Dominated
		UniversalPCR/ACS	94	1,226,000	Dominated
		Hybrid/ACS	71	1,256,000	90,000
		UniversalPCR/Hospital	95	1,901,000	Dominated
		Hybrid/Hospital	71	2,020,000	Dominated
		UniversalPCR/TempHousing	71	38,954,000	Dominated
	80				
		SxScreen/PCR/ACS	82	259,000	NA
		NoIntervention	174	540,000	Dominated
		SxScreen/PCR/Hospital	80	1,115,000	Dominated
		UniversalPCR/ACS	94	1,226,000	Dominated
		Hybrid/ACS	70	1,259,000	83,000
		UniversalPCR/Hospital	95	1,901,000	Dominated
		Hybrid/Hospital	70	2,014,000	Dominated
		UniversalPCR/TempHousing	71	38,954,000	Dominated

Abbreviations: ACS, alternate care sites; COVID-19, coronavirus disease 2019; Dominated, less clinically effective and more costly than an alternative strategy, or a combination of two alternative strategies;[14] Incr., incremental; mild/mod., mild/moderate; *PCR,* polymerase chain reaction; Sx, symptom; *SxScreen,* symptom screen; *TempHousing,* temporary housing; *UniversalPCR*, universal polymerase chain reaction test for everyone; USD, United States dollars.

© 2020 Baggett TP et al. *JAMA Network Open.*

Strategies are listed in order of ascending costs, per convention of cost-effectiveness analysis.

* Costs were rounded to the nearest thousands.

© 2020 Baggett TP et al. *JAMA Network Open.*

eTable 5. One-Way Sensitivity Analysis on Efficacy of ACS for Confirmed COVID-19 in Reducing SARS-CoV-2 Transmission

Effective reproduction number (R_e)	Efficacy of ACS-C in reducing SARS-CoV-2 transmission, %	Strategy	Cumulative infections, n	Total cost *, 2020 USD	Incr. cost per case prevented *, 2020 USD
2.6	70				
		SxScreen/PCR/ACS	1,573	4,240,000	NA
		UniversalPCR/ACS	1,831	4,494,000	Dominated
		Hybrid/ACS	1,529	5,252,000	Dominated
		NoIntervention	1,954	6,114,000	Dominated
		Hybrid/Hospital	971	12,321,000	13,000
		SxScreen/PCR/Hospital	1,138	12,738,000	Dominated
		UniversalPCR/Hospital	1,680	12,986,000	Dominated
		UniversalPCR/TempHousing	379	39,772,000	46,000
	80				
		SxScreen/PCR/ACS	1,477	3,961,000	NA
		UniversalPCR/ACS	1,786	4,391,000	Dominated
		Hybrid/ACS	1,387	4,813,000	9,000
		NoIntervention	1,954	6,114,000	Dominated
		Hybrid/Hospital	971	12,321,000	18,000
		SxScreen/PCR/Hospital	1,138	12,738,000	Dominated
		UniversalPCR/Hospital	1,680	12,986,000	Dominated
		UniversalPCR/TempHousing	379	39,772,000	46,000
	90				
		SxScreen/PCR/ACS	1,363	3,633,000	NA
		UniversalPCR/ACS	1,740	4,282,000	Dominated
		Hybrid/ACS	1,210	4,309,000	4,000
		NoIntervention	1,954	6,114,000	Dominated
		Hybrid/Hospital	971	12,321,000	34,000
		SxScreen/PCR/Hospital	1,138	12,738,000	Dominated
		UniversalPCR/Hospital	1,680	12,986,000	Dominated
		UniversalPCR/TempHousing	379	39,772,000	46,000

© 2020 Baggett TP et al. *JAMA Network Open.*

eTable 5 cont. One-Way Sensitivity Analysis on Efficacy of ACS for Confirmed COVID-19 in Reducing SARS-CoV-2 Transmission

**Effective reproduction number (R_e)**	**Efficacy of ACS-C in reducing SARS-CoV-2 transmission, %**	**Strategy**	**Cumulative infections, n**	**Total cost *, 2020 USD**	**Incr. cost per case prevented *, 2020 USD**
2.6	100				
	base case	*SxScreen/PCR/ACS*	1,239	3,267,000	NA
		Hybrid/ACS	985	3,628,000	1,000
		UniversalPCR/ACS	1,681	4,143,000	Dominated
		NoIntervention	1,954	6,098,000	Dominated
		Hybrid/Hospital	967	12,202,000	Dominated
		SxScreen/PCR/Hospital	1,133	12,620,000	Dominated
		UniversalPCR/Hospital	1,679	12,914,000	Dominated
		UniversalPCR/TempHousing	376	39,119,000	58,000
1.3	70				
		SxScreen/PCR/ACS	310	825,000	NA
		NoIntervention	544	1,485,000	Dominated
		SxScreen/PCR/Hospital	125	1,608,000	4,000
		UniversalPCR/ACS	488	1,866,000	Dominated
		Hybrid/ACS	333	1,922,000	Dominated
		Hybrid/Hospital	100	2,383,000	31,000
		UniversalPCR/Hospital	208	2,657,000	Dominated
		UniversalPCR/TempHousing	95	39,626,000	6,964,000
	80				
		SxScreen/PCR/ACS	234	641,000	NA
		NoIntervention	544	1,485,000	Dominated
		SxScreen/PCR/Hospital	125	1,608,000	9,000
		Hybrid/ACS	223	1,652,000	Dominated
		UniversalPCR/ACS	375	1,696,000	Dominated
		Hybrid/Hospital	100	2,383,000	31,000
		UniversalPCR/Hospital	208	2,657,000	Dominated
		UniversalPCR/TempHousing	95	39,626,000	6,964,000

© 2020 Baggett TP et al. *JAMA Network Open.*

eTable 5 cont. One-Way Sensitivity Analysis on Efficacy of ACS for Confirmed COVID-19 in Reducing SARS-CoV-2 Transmission

**Effective reproduction number (R_e)**	**Efficacy of ACS-C in reducing SARS-CoV-2 transmission, %**	**Strategy**	**Cumulative infections, n**	**Total cost *, 2020 USD**	**Incr. cost per case prevented *, 2020 USD**
1.3	90				
		SxScreen/PCR/ACS	174	511,000	NA
		Hybrid/ACS	145	1,469,000	Dominated
		NoIntervention	544	1,485,000	Dominated
		UniversalPCR/ACS	276	1,549,000	Dominated
		SxScreen/PCR/Hospital	125	1,608,000	22,000
		Hybrid/Hospital	100	2,383,000	31,000
		UniversalPCR/Hospital	208	2,657,000	Dominated
		UniversalPCR/TempHousing	95	39,626,000	6,964,000
	100				
	base case	*SxScreen/PCR/ACS*	137	409,000	NA
		Hybrid/ACS	103	1,325,000	27,000
		UniversalPCR/ACS	207	1,426,000	Dominated
		NoIntervention	538	1,461,000	Dominated
		SxScreen/PCR/Hospital	125	1,604,000	Dominated
		Hybrid/Hospital	100	2,368,000	382,000
		UniversalPCR/Hospital	207	2,631,000	Dominated
		UniversalPCR/TempHousing	95	38,974,000	6,854,000
0.9	70				
		SxScreen/PCR/ACS	154	452,000	NA
		NoIntervention	175	543,000	Dominated
		SxScreen/PCR/Hospital	82	1,115,000	9,000
		UniversalPCR/ACS	200	1,429,000	Dominated
		Hybrid/ACS	176	1,535,000	Dominated
		UniversalPCR/Hospital	95	1,918,000	Dominated
		Hybrid/Hospital	71	2,018,000	84,000
		UniversalPCR/TempHousing	71	39,607,000	Dominated

© 2020 Baggett TP et al. *JAMA Network Open.*

eTable 5 cont. One-Way Sensitivity Analysis on Efficacy of ACS for Confirmed COVID-19 in Reducing SARS-CoV-2 Transmission

Effective reproduction number (R_e)	Efficacy of ACS-C in reducing SARS-CoV-2 transmission, %	Strategy	Cumulative infections, n	Total cost *, 2020 USD	Incr. cost per case prevented *, 2020 USD
0.9	80				
		SxScreen/PCR/ACS	124	370,000	NA
		NoIntervention	175	543,000	Dominated
		SxScreen/PCR/Hospital	82	1,115,000	18,000
		UniversalPCR/ACS	153	1,349,000	Dominated
		Hybrid/ACS	126	1,406,000	Dominated
		UniversalPCR/Hospital	95	1,918,000	Dominated
		Hybrid/Hospital	71	2,018,000	84,000
		UniversalPCR/TempHousing	71	39,607,000	Dominated
	90				
		SxScreen/PCR/ACS	101	307,000	NA
		NoIntervention	175	543,000	Dominated
		SxScreen/PCR/Hospital	82	1,115,000	42,000
		UniversalPCR/ACS	119	1,289,000	Dominated
		Hybrid/ACS	94	1,321,000	Dominated
		UniversalPCR/Hospital	95	1,918,000	Dominated
		Hybrid/Hospital	71	2,018,000	84,000
		UniversalPCR/TempHousing	71	39,607,000	Dominated
	100				
	base case	*SxScreen/PCR/ACS*	85	264,000	NA
		NoIntervention	174	540,000	Dominated
		SxScreen/PCR/Hospital	82	1,113,000	Dominated
		UniversalPCR/ACS	94	1,226,000	Dominated
		Hybrid/ACS	71	1,240,000	71,000
		UniversalPCR/Hospital	95	1,901,000	Dominated
		Hybrid/Hospital	71	2,004,000	Dominated
		UniversalPCR/TempHousing	71	38,954,000	Dominated

© 2020 Baggett TP et al. *JAMA Network Open.*

Abbreviations: ACS, alternate care sites; COVID-19, coronavirus disease 2019; Dominated, less clinically effective and more costly than an alternative strategy, or a combination of two alternative strategies;[14] Incr., incremental; *PCR,* polymerase chain reaction; *SxScreen,* symptom screen; *TempHousing,* temporary housing; *UniversalPCR*, universal polymerase chain reaction test for everyone; USD, United States dollars.

Strategies are listed in order of ascending costs, per convention of cost-effectiveness analysis.

* Costs were rounded to the nearest thousands.

© 2020 Baggett TP et al. *JAMA Network Open.*

eTable 6. One-Way Sensitivity Analysis on Efficacy of Temporary Housing in Reducing SARS-CoV-2 Transmission

Effective reproduction number (R_e)	Efficacy of TempHousing in reducing SARS-CoV-2 transmission, %	Strategy	Cumulative infections, n	Total cost *, 2020 USD	Incr. cost per case prevented *, 2020 USD
2.6	50				
		SxScreen/PCR/ACS	1,244	3,295,000	NA
		Hybrid/ACS	989	3,669,000	1,000
		UniversalPCR/ACS	1,682	4,176,000	Dominated
		NoIntervention	1,954	6,114,000	Dominated
		Hybrid/Hospital	971	12,321,000	Dominated
		SxScreen/PCR/Hospital	1,138	12,738,000	Dominated
		UniversalPCR/Hospital	1,680	12,986,000	Dominated
		UniversalPCR/TempHousing	798	39,975,000	190,000
	60				
	base case	*SxScreen/PCR/ACS*	1,239	3,267,000	NA
		Hybrid/ACS	985	3,628,000	1,000
		UniversalPCR/ACS	1,681	4,143,000	Dominated
		NoIntervention	1,954	6,098,000	Dominated
		Hybrid/Hospital	967	12,202,000	Dominated
		SxScreen/PCR/Hospital	1,133	12,620,000	Dominated
		UniversalPCR/Hospital	1,679	12,914,000	Dominated
		UniversalPCR/TempHousing	376	39,119,000	58,000
	70				
		SxScreen/PCR/ACS	1,244	3,295,000	NA
		Hybrid/ACS	989	3,669,000	1,000
		UniversalPCR/ACS	1,682	4,176,000	Dominated
		NoIntervention	1,954	6,114,000	Dominated
		Hybrid/Hospital	971	12,321,000	Dominated
		SxScreen/PCR/Hospital	1,138	12,738,000	Dominated
		UniversalPCR/Hospital	1,680	12,986,000	Dominated
		UniversalPCR/TempHousing	180	39,672,000	45,000

© 2020 Baggett TP et al. *JAMA Network Open.*

eTable 6 cont. One-Way Sensitivity Analysis on Efficacy of Temporary Housing in Reducing SARS-CoV-2 Transmission

Effective reproduction number (R_e)	Efficacy of TempHousing in reducing SARS-CoV-2 transmission, %	Strategy	Cumulative infections, n	Total cost *, 2020 USD	Incr. cost per case prevented *, 2020 USD
2.6	80				
		SxScreen/PCR/ACS	1,244	3,295,000	NA
		Hybrid/ACS	989	3,669,000	1,000
		UniversalPCR/ACS	1,682	4,176,000	Dominated
		NoIntervention	1,954	6,114,000	Dominated
		Hybrid/Hospital	971	12,321,000	Dominated
		SxScreen/PCR/Hospital	1,138	12,738,000	Dominated
		UniversalPCR/Hospital	1,680	12,986,000	Dominated
		UniversalPCR/TempHousing	104	39,616,000	41,000
1.3	50				
		SxScreen/PCR/ACS	137	409,000	NA
		Hybrid/ACS	103	1,341,000	27,000
		UniversalPCR/ACS	208	1,444,000	Dominated
		NoIntervention	544	1,485,000	Dominated
		SxScreen/PCR/Hospital	125	1,608,000	Dominated
		Hybrid/Hospital	100	2,383,000	379,000
		UniversalPCR/Hospital	208	2,657,000	Dominated
		UniversalPCR/TempHousing	121	39,630,000	Dominated
	60				
	base case	*SxScreen/PCR/ACS*	137	409,000	NA
		Hybrid/ACS	103	1,325,000	27,000
		UniversalPCR/ACS	207	1,426,000	Dominated
		NoIntervention	538	1,461,000	Dominated
		SxScreen/PCR/Hospital	125	1,604,000	Dominated
		Hybrid/Hospital	100	2,368,000	382,000
		UniversalPCR/Hospital	207	2,631,000	Dominated
		UniversalPCR/TempHousing	95	38,974,000	6,854,000

© 2020 Baggett TP et al. *JAMA Network Open.*

eTable 6 cont. One-Way Sensitivity Analysis on Efficacy of Temporary Housing in Reducing SARS-CoV-2 Transmission

Effective reproduction number (R_e)	Efficacy of TempHousing in reducing SARS-CoV-2 transmission, %	Strategy	Cumulative infections, n	Total cost *, 2020 USD	Incr. cost per case prevented *, 2020 USD
1.3	70				
		SxScreen/PCR/ACS	137	409,000	NA
		Hybrid/ACS	103	1,341,000	27,000
		UniversalPCR/ACS	208	1,444,000	Dominated
		NoIntervention	544	1,485,000	Dominated
		SxScreen/PCR/Hospital	125	1,608,000	Dominated
		Hybrid/Hospital	100	2,383,000	379,000
		UniversalPCR/Hospital	208	2,657,000	Dominated
		UniversalPCR/TempHousing	76	39,601,000	1,529,000
	80				
		SxScreen/PCR/ACS	137	409,000	NA
		Hybrid/ACS	103	1,341,000	27,000
		UniversalPCR/ACS	208	1,444,000	Dominated
		NoIntervention	544	1,485,000	Dominated
		SxScreen/PCR/Hospital	125	1,608,000	Dominated
		Hybrid/Hospital	100	2,383,000	379,000
		UniversalPCR/Hospital	208	2,657,000	Dominated
		UniversalPCR/TempHousing	65	39,605,000	1,053,000
0.9	50				
		SxScreen/PCR/ACS	85	264,000	NA
		NoIntervention	175	543,000	Dominated
		SxScreen/PCR/Hospital	82	1,115,000	Dominated
		UniversalPCR/ACS	95	1,242,000	Dominated
		Hybrid/ACS	71	1,256,000	72,000
		UniversalPCR/Hospital	95	1,918,000	Dominated
		Hybrid/Hospital	71	2,018,000	Dominated
		UniversalPCR/TempHousing	80	39,605,000	Dominated

© 2020 Baggett TP et al. *JAMA Network Open.*

eTable 6 cont. One-Way Sensitivity Analysis on Efficacy of Temporary Housing in Reducing SARS-CoV-2 Transmission

**Effective reproduction number (R_e)**	**Efficacy of TempHousing in reducing SARS-CoV-2 transmission, %**	**Strategy**	**Cumulative infections, n**	**Total cost *, 2020 USD**	**Incr. cost per case prevented *, 2020 USD**
0.9	60				
	base case	*SxScreen/PCR/ACS*	85	264,000	NA
		NoIntervention	174	540,000	Dominated
		SxScreen/PCR/Hospital	82	1,113,000	Dominated
		UniversalPCR/ACS	94	1,226,000	Dominated
		Hybrid/ACS	71	1,240,000	71,000
		UniversalPCR/Hospital	95	1,901,000	Dominated
		Hybrid/Hospital	71	2,004,000	Dominated
		UniversalPCR/TempHousing	71	38,954,000	Dominated
	70				
		SxScreen/PCR/ACS	85	264,000	NA
		NoIntervention	175	543,000	Dominated
		SxScreen/PCR/Hospital	82	1,115,000	Dominated
		UniversalPCR/ACS	95	1,242,000	Dominated
		Hybrid/ACS	71	1,256,000	72,000
		UniversalPCR/Hospital	95	1,918,000	Dominated
		Hybrid/Hospital	71	2,018,000	Dominated
		UniversalPCR/TempHousing	63	39,600,000	4,668,000
	80				
		SxScreen/PCR/ACS	85	264,000	NA
		NoIntervention	175	543,000	Dominated
		SxScreen/PCR/Hospital	82	1,115,000	Dominated
		UniversalPCR/ACS	95	1,242,000	Dominated
		Hybrid/ACS	71	1,256,000	72,000
		UniversalPCR/Hospital	95	1,918,000	Dominated
		Hybrid/Hospital	71	2,018,000	Dominated
		UniversalPCR/TempHousing	57	39,596,000	2,697,000

© 2020 Baggett TP et al. *JAMA Network Open.*

Abbreviations: ACS, alternate care sites; COVID-19, coronavirus disease 2019; Dominated, less clinically effective and more costly than an alternative strategy, or a combination of two alternative strategies;[14] Incr., incremental; *PCR,* polymerase chain reaction; *SxScreen,* symptom screen; *TempHousing,* temporary housing; *UniversalPCR*, universal polymerase chain reaction test for everyone; USD, United States dollars.

Strategies are listed in order of ascending costs, per convention of cost-effectiveness analysis.

* Costs were rounded to the nearest thousands.

© 2020 Baggett TP et al. *JAMA Network Open.*

eTable 7. One-Way Sensitivity Analysis on Cost of a PCR Test

**Effective reproduction number (R_e)**	**PCR test cost, 2020 USD**	**Strategy**	**Cumulative infections, n**	**Total cost *, 2020 USD**	**Incr. cost per case prevented *, 2020 USD**
2.6	5				
		Hybrid/ACS	989	2,711,000	NA
		UniversalPCR/ACS	1,681	3,245,000	Dominated
		SxScreen/PCR/ACS	1,239	3,246,000	Dominated
		NoIntervention	1,954	6,097,000	Dominated
		Hybrid/Hospital	967	11,364,000	Dominated
		UniversalPCR/Hospital	1,679	12,056,000	Dominated
		SxScreen/PCR/Hospital	1,133	12,693,000	Dominated
		UniversalPCR/TempHousing	379	38,843,000	59,000
	10				
		Hybrid/ACS	985	2,815,000	NA
		SxScreen/PCR/ACS	1,239	3,251,000	Dominated
		UniversalPCR/ACS	1,681	3,346,000	Dominated
		NoIntervention	1,954	6,099,000	Dominated
		Hybrid/Hospital	967	11,468,000	Dominated
		UniversalPCR/Hospital	1,679	12,157,000	Dominated
		SxScreen/PCR/Hospital	1,133	12,698,000	Dominated
		UniversalPCR/TempHousing	379	38,944,000	59,000
	25				
		Hybrid/ACS	985	3,127,000	NA
		SxScreen/PCR/ACS	1,239	3,267,000	Dominated
		UniversalPCR/ACS	1,681	3,650,000	Dominated
		NoIntervention	1,954	6,105,000	Dominated
		Hybrid/Hospital	967	11,780,000	Dominated
		UniversalPCR/Hospital	1,679	12,460,000	Dominated
		SxScreen/PCR/Hospital	1,133	12,713,000	Dominated
		UniversalPCR/TempHousing	379	39,247,000	59,000

© 2020 Baggett TP et al. *JAMA Network Open.*

eTable 7 cont. One-Way Sensitivity Analysis on Cost of a PCR Test

Effective reproduction number (R_e)	PCR test cost, 2020 USD	Strategy	Cumulative infections, n	Total cost *, 2020 USD	Incr. cost per case prevented *, 2020 USD
2.6	51				
	base case	*SxScreen/PCR/ACS*	1,239	3,267,000	NA
		Hybrid/ACS	985	3,628,000	1,000
		UniversalPCR/ACS	1,681	4,143,000	Dominated
		NoIntervention	1,954	6,098,000	Dominated
		Hybrid/Hospital	967	12,202,000	Dominated
		SxScreen/PCR/Hospital	1,133	12,620,000	Dominated
		UniversalPCR/Hospital	1,679	12,914,000	Dominated
		UniversalPCR/TempHousing	376	39,119,000	58,000
	75				
		SxScreen/PCR/ACS	1,239	3,320,000	NA
		Hybrid/ACS	985	4,168,000	3,000
		UniversalPCR/ACS	1,681	4,662,000	Dominated
		NoIntervention	1,954	6,123,000	Dominated
		SxScreen/PCR/Hospital	1,133	12,761,000	Dominated
		Hybrid/Hospital	967	12,821,000	Dominated
		UniversalPCR/Hospital	1,679	13,472,000	Dominated
		UniversalPCR/TempHousing	379	40,257,000	43,000
	100				
		SxScreen/PCR/ACS	1,239	3,373,000	NA
		Hybrid/ACS	985	4,708,000	5,000
		UniversalPCR/ACS	1,681	5,181,000	Dominated
		NoIntervention	1,954	6,148,000	Dominated
		Hybrid/Hospital	967	13,440,000	Dominated
		SxScreen/PCR/Hospital	1,133	12,902,000	Dominated
		UniversalPCR/Hospital	1,679	14,030,000	Dominated
		UniversalPCR/TempHousing	379	40,762,000	43,000

© 2020 Baggett TP et al. *JAMA Network Open.*

eTable 7 cont. One-Way Sensitivity Analysis on Cost of a PCR Test

Effective reproduction number (R_e)	PCR test cost, 2020 USD	Strategy	Cumulative infections, n	Total cost *, 2020 USD	Incr. cost per case prevented *, 2020 USD
1.3	5				
		SxScreen/PCR/ACS	137	402,000	NA
		Hybrid/ACS	103	409,000	200
		UniversalPCR/ACS	207	515,000	Dominated
		Hybrid/Hospital	100	1,451,000	379,000
		NoIntervention	538	1,480,000	Dominated
		SxScreen/PCR/Hospital	125	1,601,000	Dominated
		UniversalPCR/Hospital	207	1,728,000	Dominated
		UniversalPCR/TempHousing	95	38,696,000	6,965,000
	10				
		SxScreen/PCR/ACS	137	403,000	NA
		Hybrid/ACS	103	510,000	3,000
		UniversalPCR/ACS	207	616,000	Dominated
		NoIntervention	538	1,481,000	Dominated
		Hybrid/Hospital	100	1,553,000	379,000
		SxScreen/PCR/Hospital	125	1,602,000	Dominated
		UniversalPCR/Hospital	207	1,829,000	Dominated
		UniversalPCR/TempHousing	95	38,797,000	6,964,000
	25				
		SxScreen/PCR/ACS	137	405,000	NA
		Hybrid/ACS	103	814,000	12,000
		UniversalPCR/ACS	207	919,000	Dominated
		NoIntervention	538	1,482,000	Dominated
		SxScreen/PCR/Hospital	125	1,604,000	Dominated
		Hybrid/Hospital	100	1,856,000	379,000
		UniversalPCR/Hospital	207	2,132,000	Dominated
		UniversalPCR/TempHousing	95	39,101,000	6,965,000

© 2020 Baggett TP et al. *JAMA Network Open.*

eTable 7 cont. One-Way Sensitivity Analysis on Cost of a PCR Test

Effective reproduction number (R_e)	PCR test cost, 2020 USD	Strategy	Cumulative infections, n	Total cost *, 2020 USD	Incr. cost per case prevented *, 2020 USD
1.3	51				
	base case	*SxScreen/PCR/ACS*	137	409,000	NA
		Hybrid/ACS	103	1,325,000	27,000
		UniversalPCR/ACS	207	1,426,000	Dominated
		NoIntervention	538	1,461,000	Dominated
		SxScreen/PCR/Hospital	125	1,604,000	Dominated
		Hybrid/Hospital	100	2,368,000	382,000
		UniversalPCR/Hospital	207	2,631,000	Dominated
		UniversalPCR/TempHousing	95	38,974,000	6,854,000
	75				
		SxScreen/PCR/ACS	137	413,000	NA
		NoIntervention	538	1,487,000	Dominated
		SxScreen/PCR/Hospital	125	1,611,000	Dominated
		Hybrid/ACS	103	1,827,000	41,000
		UniversalPCR/ACS	207	1,928,000	Dominated
		Hybrid/Hospital	100	2,869,000	379,000
		UniversalPCR/Hospital	207	3,142,000	Dominated
		UniversalPCR/TempHousing	95	40,111,000	6,964,000
	100				
		SxScreen/PCR/ACS	137	417,000	NA
		NoIntervention	538	1,513,000	Dominated
		SxScreen/PCR/Hospital	125	1,618,000	Dominated
		Hybrid/ACS	103	2,329,000	56,000
		UniversalPCR/ACS	207	2,430,000	Dominated
		Hybrid/Hospital	100	3,370,000	347,000
		UniversalPCR/Hospital	207	3,653,000	Dominated
		UniversalPCR/TempHousing	95	40,616,000	7,449,000

© 2020 Baggett TP et al. *JAMA Network Open.*

eTable 7 cont. One-Way Sensitivity Analysis on Cost of a PCR Test

Effective reproduction number (R_e)	PCR test cost, 2020 USD	Strategy	Cumulative infections, n	Total cost *, 2020 USD	Incr. cost per case prevented *, 2020 USD
0.9	5				
		SxScreen/PCR/ACS	85	259,000	NA
		UniversalPCR/ACS	95	314,000	Dominated
		Hybrid/ACS	71	325,000	5,000
		NoIntervention	175	542,000	Dominated
		UniversalPCR/Hospital	95	989,000	Dominated
		Hybrid/Hospital	71	1,087,000	Dominated
		SxScreen/PCR/Hospital	82	1,110,000	Dominated
		UniversalPCR/TempHousing	71	38,677,000	Dominated
	10				
		SxScreen/PCR/ACS	85	260,000	NA
		UniversalPCR/ACS	95	415,000	Dominated
		Hybrid/ACS	71	426,000	12,000
		NoIntervention	175	542,000	Dominated
		UniversalPCR/Hospital	95	1,090,000	Dominated
		SxScreen/PCR/Hospital	82	1,111,000	Dominated
		Hybrid/Hospital	71	1,189,000	Dominated
		UniversalPCR/TempHousing	71	38,778,000	Dominated
	25				
		SxScreen/PCR/ACS	85	262,000	NA
		NoIntervention	175	542,000	Dominated
		UniversalPCR/ACS	95	717,000	Dominated
		Hybrid/ACS	71	730,000	34,000
		SxScreen/PCR/Hospital	82	1,113,000	Dominated
		UniversalPCR/Hospital	95	1,393,000	Dominated
		Hybrid/Hospital	71	1,492,000	Dominated
		UniversalPCR/TempHousing	71	39,081,000	Dominated

© 2020 Baggett TP et al. *JAMA Network Open.*

eTable 7 cont. One-Way Sensitivity Analysis on Cost of a PCR Test

Effective reproduction number (R_e)	PCR test cost, 2020 USD	Strategy	Cumulative infections, n	Total cost *, 2020 USD	Incr. cost per case prevented *, 2020 USD
0.9	51				
	base case	*SxScreen/PCR/ACS*	85	264,000	NA
		NoIntervention	174	540,000	Dominated
		SxScreen/PCR/Hospital	82	1,113,000	Dominated
		UniversalPCR/ACS	94	1,226,000	Dominated
		Hybrid/ACS	71	1,240,000	71,000
		UniversalPCR/Hospital	95	1,901,000	Dominated
		Hybrid/Hospital	71	2,004,000	Dominated
		UniversalPCR/TempHousing	71	38,954,000	Dominated
	75				
		SxScreen/PCR/ACS	85	267,000	NA
		NoIntervention	175	544,000	Dominated
		SxScreen/PCR/Hospital	82	1,118,000	Dominated
		UniversalPCR/ACS	95	1,727,000	Dominated
		Hybrid/ACS	71	1,742,000	107,000
		UniversalPCR/Hospital	95	2,402,000	Dominated
		Hybrid/Hospital	71	2,504,000	Dominated
		UniversalPCR/TempHousing	71	40,092,000	Dominated
	100				
		SxScreen/PCR/ACS	85	270,000	NA
		NoIntervention	174	548,000	Dominated
		SxScreen/PCR/Hospital	82	1,123,000	Dominated
		UniversalPCR/ACS	94	2,228,000	Dominated
		Hybrid/ACS	71	2,244,000	141,000
		UniversalPCR/Hospital	95	2,903,000	Dominated
		Hybrid/Hospital	71	3,004,000	Dominated
		UniversalPCR/TempHousing	71	40,597,000	Dominated

Abbreviations: ACS, alternate care sites; COVID-19, coronavirus disease 2019; Dominated, less clinically effective and more costly than an alternative strategy, or a combination of two alternative strategies;[14] Incr., incremental; *PCR,* polymerase chain reaction; *SxScreen,* symptom screen; *TempHousing,* temporary housing; *UniversalPCR*, universal polymerase chain reaction test for everyone; USD, United States dollars.

© 2020 Baggett TP et al. *JAMA Network Open.*

Strategies are listed in order of ascending costs, per convention of cost-effectiveness analysis.

* Costs were rounded to the nearest thousands.

© 2020 Baggett TP et al. *JAMA Network Open.*

eTable 8. One-Way Sensitivity Analysis on Cost of Symptom Screen

Effective reproduction number (R_e)	Cost of symptom screen, 2020 USD	Strategy	Cumulative infections, n	Total cost *, 2020 USD	Incr. cost per case prevented *, 2020 USD
2.6	0				
	base case	*SxScreen/PCR/ACS*	1,239	3,267,000	NA
		Hybrid/ACS	985	3,628,000	1,000
		UniversalPCR/ACS	1,681	4,143,000	Dominated
		NoIntervention	1,954	6,098,000	Dominated
		Hybrid/Hospital	967	12,202,000	Dominated
		SxScreen/PCR/Hospital	1,133	12,620,000	Dominated
		UniversalPCR/Hospital	1,679	12,914,000	Dominated
		UniversalPCR/TempHousing	376	39,119,000	58,000
	1				
		SxScreen/PCR/ACS	1,244	3,562,000	NA
		Hybrid/ACS	989	3,937,000	1,000
		UniversalPCR/ACS	1,682	4,176,000	Dominated
		NoIntervention	1,954	6,114,000	Dominated
		Hybrid/Hospital	971	12,590,000	Dominated
		UniversalPCR/Hospital	1,680	12,986,000	Dominated
		SxScreen/PCR/Hospital	1,138	13,006,000	Dominated
		UniversalPCR/TempHousing	379	39,772,000	59,000
	5				
		UniversalPCR/ACS	1,682	4,176,000	NA
		SxScreen/PCR/ACS	1,244	4,629,000	1,000
		Hybrid/ACS	989	5,011,000	2,000
		NoIntervention	1,954	6,114,000	Dominated
		UniversalPCR/Hospital	1,680	12,986,000	Dominated
		Hybrid/Hospital	971	13,664,000	Dominated
		SxScreen/PCR/Hospital	1,138	14,077,000	Dominated
		UniversalPCR/TempHousing	379	39,772,000	57,000

© 2020 Baggett TP et al. *JAMA Network Open.*

eTable 8 cont. One-Way Sensitivity Analysis on Cost of Symptom Screen

Effective reproduction number (R_e)	Cost of symptom screen, 2020 USD	Strategy	Cumulative infections, n	Total cost *, 2020 USD	Incr. cost per case prevented *, 2020 USD
1.3	0				
	base case	*SxScreen/PCR/ACS*	137	409,000	NA
		Hybrid/ACS	103	1,325,000	27,000
		UniversalPCR/ACS	207	1,426,000	Dominated
		NoIntervention	538	1,461,000	Dominated
		SxScreen/PCR/Hospital	125	1,604,000	Dominated
		Hybrid/Hospital	100	2,368,000	382,000
		UniversalPCR/Hospital	207	2,631,000	Dominated
		UniversalPCR/TempHousing	95	38,974,000	6,854,000
	1				
		SxScreen/PCR/ACS	137	684,000	NA
		UniversalPCR/ACS	208	1,444,000	Dominated
		NoIntervention	544	1,485,000	Dominated
		Hybrid/ACS	103	1,616,000	27,000
		SxScreen/PCR/Hospital	125	1,882,000	Dominated
		UniversalPCR/Hospital	208	2,657,000	Dominated
		Hybrid/Hospital	100	2,658,000	379,000
		UniversalPCR/TempHousing	95	39,626,000	6,913,000
	5				
		UniversalPCR/ACS	208	1,444,000	NA
		NoIntervention	544	1,485,000	Dominated
		SxScreen/PCR/ACS	137	1,781,000	5,000
		UniversalPCR/Hospital	208	2,657,000	Dominated
		Hybrid/ACS	103	2,714,000	27,000
		SxScreen/PCR/Hospital	125	2,980,000	Dominated
		Hybrid/Hospital	100	3,756,000	379,000
		UniversalPCR/TempHousing	95	39,626,000	6,707,000

© 2020 Baggett TP et al. *JAMA Network Open.*

eTable 8 cont. One-Way Sensitivity Analysis on Cost of Symptom Screen

Effective reproduction number (R_e)	Cost of symptom screen, 2020 USD	Strategy	Cumulative infections, n	Total cost *, 2020 USD	Incr. cost per case prevented *, 2020 USD
0.9	0				
	base case	*SxScreen/PCR/ACS*	85	264,000	NA
		NoIntervention	174	540,000	Dominated
		SxScreen/PCR/Hospital	82	1,113,000	Dominated
		UniversalPCR/ACS	94	1,226,000	Dominated
		Hybrid/ACS	71	1,240,000	71,000
		UniversalPCR/Hospital	95	1,901,000	Dominated
		Hybrid/Hospital	71	2,004,000	Dominated
		UniversalPCR/TempHousing	71	38,954,000	Dominated
	1				
		SxScreen/PCR/ACS	85	539,000	NA
		NoIntervention	175	543,000	Dominated
		UniversalPCR/ACS	95	1,242,000	Dominated
		SxScreen/PCR/Hospital	82	1,390,000	Dominated
		Hybrid/ACS	71	1,531,000	72,000
		UniversalPCR/Hospital	95	1,918,000	Dominated
		Hybrid/Hospital	71	2,293,000	Dominated
		UniversalPCR/TempHousing	71	39,607,000	Dominated
	5				
		NoIntervention	175	543,000	NA
		UniversalPCR/ACS	95	1,242,000	9,000
		SxScreen/PCR/ACS	85	1,638,000	42,000
		UniversalPCR/Hospital	95	1,918,000	Dominated
		SxScreen/PCR/Hospital	82	2,489,000	Dominated
		Hybrid/ACS	71	2,630,000	72,000
		Hybrid/Hospital	71	3,393,000	Dominated
		UniversalPCR/TempHousing	71	39,607,000	Dominated

© 2020 Baggett TP et al. *JAMA Network Open.*

Abbreviations: ACS, alternate care sites; COVID-19, coronavirus disease 2019; Dominated, less clinically effective and more costly than an alternative strategy, or a combination of two alternative strategies;[14] Incr., incremental; *PCR,* polymerase chain reaction; *SxScreen,* symptom screen; *TempHousing,* temporary housing; *UniversalPCR*, universal polymerase chain reaction test for everyone; USD, United States dollars.

Strategies are listed in order of ascending costs, per convention of cost-effectiveness analysis.

* Costs were rounded to the nearest thousands.

© 2020 Baggett TP et al. *JAMA Network Open.*

eTable 9. One-Way Sensitivity Analysis on Daily Costs of Hospital Beds

Effective reproduction number (R_e)	Daily costs of hospital beds	Strategy	Cumulative infections, n	Total cost *, 2020 USD	Incr. cost per case prevented *, 2020 USD
2.6	0.5X base case				
		SxScreen/PCR/ACS	1,239	2,903,000	NA
		NoIntervention	1,954	3,065,000	Dominated
		Hybrid/ACS	985	3,365,000	2,000
		UniversalPCR/ACS	1,681	3,608,000	Dominated
		SxScreen/PCR/Hospital	1,133	6,390,000	Dominated
		Hybrid/Hospital	967	6,688,000	Dominated
		UniversalPCR/Hospital	1,680	7,006,000	Dominated
		UniversalPCR/TempHousing	376	39,650,000	59,000
	Base case				
		SxScreen/PCR/ACS	1,239	3,267,000	NA
		Hybrid/ACS	985	3,628,000	1,000
		UniversalPCR/ACS	1,681	4,143,000	Dominated
		NoIntervention	1,954	6,098,000	Dominated
		Hybrid/Hospital	967	12,202,000	Dominated
		SxScreen/PCR/Hospital	1,133	12,620,000	Dominated
		UniversalPCR/Hospital	1,679	12,914,000	Dominated
		UniversalPCR/TempHousing	376	39,119,000	58,000
	1.5X base case				
		SxScreen/PCR/ACS	1,239	3,687,000	NA
		Hybrid/ACS	985	3,972,000	1,000
		UniversalPCR/ACS	1,681	4,744,000	Dominated
		NoIntervention	1,954	9,164,000	Dominated
		Hybrid/Hospital	967	17,955,000	Dominated
		UniversalPCR/Hospital	1,680	18,967,000	Dominated
		SxScreen/PCR/Hospital	1,138	19,086,000	Dominated
		UniversalPCR/TempHousing	376	39,894,000	59,000

© 2020 Baggett TP et al. *JAMA Network Open.*

eTable 9 cont. One-Way Sensitivity Analysis on Daily Costs of Hospital Beds

Effective reproduction number (R_e)	Daily costs of hospital beds	Strategy	Cumulative infections, n	Total cost *, 2020 USD	Incr. cost per case prevented *, 2020 USD
1.3	0.5X base case				
		SxScreen/PCR/ACS	137	361,000	NA
		NoIntervention	544	744,000	Dominated
		SxScreen/PCR/Hospital	125	807,000	Dominated
		Hybrid/ACS	103	1,307,000	28,000
		UniversalPCR/ACS	208	1,375,000	Dominated
		Hybrid/Hospital	100	1,708,000	146,000
		UniversalPCR/Hospital	208	1,843,000	Dominated
		UniversalPCR/TempHousing	95	39,586,000	7,083,000
	Base case				
		SxScreen/PCR/ACS	137	409,000	NA
		Hybrid/ACS	103	1,325,000	27,000
		UniversalPCR/ACS	207	1,426,000	Dominated
		No Intervention	538	1,461,000	Dominated
		SxScreen/Hospital	125	1,604,000	Dominated
		Hybrid/Hospital	100	2,368,000	382,000
		UniversalPCR/Hospital	207	2,631,000	Dominated
		UniversalPCR/TempHousing	95	38,974,000	6,854,000
	1.5X base case				
		SxScreen/PCR/ACS	137	458,000	NA
		Hybrid/ACS	103	1,375,000	27,000
		UniversalPCR/ACS	208	1,512,000	Dominated
		NoIntervention	544	2,225,000	Dominated
		SxScreen/PCR/Hospital	125	2,408,000	Dominated
		Hybrid/Hospital	100	3,058,000	611,000
		UniversalPCR/Hospital	208	3,471,000	Dominated
		UniversalPCR/TempHousing	95	39,666,000	6,845,000

© 2020 Baggett TP et al. *JAMA Network Open.*

eTable 9 cont. One-Way Sensitivity Analysis on Daily Costs of Hospital Beds

Effective reproduction number (R_e)	Daily costs of hospital beds	Strategy	Cumulative infections, n	Total cost *, 2020 USD	Incr. cost per case prevented *, 2020 USD
0.9	0.5X base case				
		SxScreen/PCR/ACS	85	234,000	NA
		NoIntervention	174	272,000	Dominated
		SxScreen/PCR/Hospital	82	560,000	Dominated
		UniversalPCR/ACS	94	1,211,000	Dominated
		Hybrid/ACS	71	1,230,000	72,000
		UniversalPCR/Hospital	95	1,473,000	Dominated
		Hybrid/Hospital	71	1,525,000	Dominated
		UniversalPCR/TempHousing	71	39,578,000	178,311,000
	Base case				
		SxScreen/PCR/ACS	85	264,000	NA
		NoIntervention	174	540,000	Dominated
		SxScreen/PCR/Hospital	82	1,113,000	Dominated
		UniversalPCR/ACS	94	1,226,000	Dominated
		Hybrid/ACS	71	1,240,000	71,000
		UniversalPCR/Hospital	95	1,901,000	Dominated
		Hybrid/Hospital	71	2,004,000	Dominated
		UniversalPCR/TempHousing	71	38,954,000	Dominated
	1.5X base case				
		SxScreen/PCR/ACS	85	295,000	NA
		NoIntervention	174	814,000	Dominated
		UniversalPCR/ACS	82	1,273,000	Dominated
		Hybrid/ACS	94	1,282,000	71,000
		SxScreen/PCR/Hospital	71	1,671,000	Dominated
		UniversalPCR/Hospital	95	2,362,000	Dominated
		Hybrid/Hospital	71	2,512,000	Dominated
		UniversalPCR/TempHousing	71	39,636,000	178,339,000

Abbreviations: ACS, alternate care sites; COVID-19, coronavirus disease 2019; Dominated, less clinically effective and more costly than an alternative strategy, or a combination of two alternative strategies;[14] Incr., incremental; *PCR,* polymerase chain reaction; *SxScreen,* symptom screen; *TempHousing,* temporary housing; *UniversalPCR*, universal polymerase chain reaction test for everyone; USD, United States dollars.

© 2020 Baggett TP et al. *JAMA Network Open.*

Strategies are listed in order of ascending costs, per convention of cost-effectiveness analysis.

* Costs were rounded to the nearest thousands.

© 2020 Baggett TP et al. *JAMA Network Open.*

eTable 10. One-Way Sensitivity Analysis on Daily Cost of an ACS

**Effective reproduction number (R_e)**	**Daily cost of an ACS, 2020 USD**	**Strategy**	**Cumulative infections, n**	**Total cost *, 2020 USD**	**Incr. cost per case prevented *, 2020 USD**
2.6	150				
		SxScreen/PCR/ACS	1,244	2,050,000	NA
		Hybrid/ACS	989	2,656,000	2,000
		UniversalPCR/ACS	1,682	3,159,000	Dominated
		NoIntervention	1,954	6,114,000	Dominated
		Hybrid/Hospital	971	12,321,000	Dominated
		SxScreen/PCR/Hospital	1,138	12,738,000	Dominated
		UniversalPCR/Hospital	1,680	12,986,000	Dominated
		UniversalPCR/TempHousing	379	39,772,000	61,000
	300				
	base case	*SxScreen/PCR/ACS*	1,244	3,262,000	NA
		Hybrid/ACS	989	3,642,000	1,000
		UniversalPCR/ACS	1,682	4,150,000	Dominated
		NoIntervention	1,954	6,114,000	Dominated
		Hybrid/Hospital	971	12,321,000	Dominated
		SxScreen/PCR/Hospital	1,138	12,738,000	Dominated
		UniversalPCR/Hospital	1,680	12,986,000	Dominated
		UniversalPCR/TempHousing	376	39,119,000	58,000
	450				
		SxScreen/PCR/ACS	1,244	4,475,000	NA
		Hybrid/ACS	989	4,629,000	1,000
		UniversalPCR/ACS	1,682	5,141,000	Dominated
		NoIntervention	1,954	6,114,000	Dominated
		Hybrid/Hospital	971	12,321,000	Dominated
		SxScreen/PCR/Hospital	1,138	12,738,000	Dominated
		UniversalPCR/Hospital	1,680	12,986,000	Dominated
		UniversalPCR/TempHousing	379	39,772,000	58,000

© 2020 Baggett TP et al. *JAMA Network Open.*

eTable 10 cont. One-Way Sensitivity Analysis on Daily Cost of an ACS

Effective reproduction number (R_e)	Daily cost of an ACS, 2020 USD	Strategy	Cumulative infections, n	Total cost *, 2020 USD	Incr. cost per case prevented *, 2020 USD
1.3	150				
		SxScreen/PCR/ACS	137	255,000	NA
		Hybrid/ACS	103	1,219,000	28,000
		UniversalPCR/ACS	208	1,304,000	Dominated
		NoIntervention	544	1,485,000	Dominated
		SxScreen/PCR/Hospital	125	1,608,000	Dominated
		Hybrid/Hospital	100	2,383,000	423,000
		UniversalPCR/Hospital	208	2,657,000	Dominated
		UniversalPCR/TempHousing	95	39,626,000	6,964,000
	300				
	base case	*SxScreen/PCR/ACS*	137	405,000	NA
		Hybrid/ACS	103	1,338,000	27,000
		UniversalPCR/ACS	208	1,440,000	Dominated
		NoIntervention	544	1,485,000	Dominated
		SxScreen/PCR/Hospital	125	1,608,000	Dominated
		Hybrid/Hospital	100	2,383,000	380,000
		UniversalPCR/Hospital	208	2,657,000	Dominated
		UniversalPCR/TempHousing	95	38,974,000	6,854,000
	450				
		SxScreen/PCR/ACS	137	555,000	NA
		Hybrid/ACS	103	1,456,000	26,000
		NoIntervention	544	1,485,000	Dominated
		UniversalPCR/ACS	208	1,577,000	Dominated
		SxScreen/PCR/Hospital	125	1,608,000	Dominated
		Hybrid/Hospital	100	2,383,000	337,000
		UniversalPCR/Hospital	208	2,657,000	Dominated
		UniversalPCR/TempHousing	95	39,626,000	6,964,000

© 2020 Baggett TP et al. *JAMA Network Open.*

eTable 10 cont. One-Way Sensitivity Analysis on Daily Cost of an ACS

**Effective reproduction number (R_e)**	**Daily cost of an ACS, 2020 USD**	**Strategy**	**Cumulative infections, n**	**Total cost *, 2020 USD**	**Incr. cost per case prevented *, 2020 USD**
0.9	150				
		SxScreen/PCR/ACS	85	164,000	NA
		NoIntervention	175	543,000	Dominated
		SxScreen/PCR/Hospital	82	1,115,000	Dominated
		UniversalPCR/ACS	95	1,166,000	Dominated
		Hybrid/ACS	71	1,168,000	73,000
		UniversalPCR/Hospital	95	1,918,000	Dominated
		Hybrid/Hospital	71	2,018,000	Dominated
		UniversalPCR/TempHousing	71	39,607,000	Dominated
	300				
	base case	*SxScreen/PCR/ACS*	85	262,000	NA
		NoIntervention	175	543,000	Dominated
		SxScreen/PCR/Hospital	82	1,115,000	Dominated
		UniversalPCR/ACS	95	1,240,000	Dominated
		Hybrid/ACS	71	1,254,000	72,000
		UniversalPCR/Hospital	95	1,918,000	Dominated
		Hybrid/Hospital	71	2,018,000	Dominated
		UniversalPCR/TempHousing	71	38,954,000	Dominated
	450				
		SxScreen/PCR/ACS	85	360,000	NA
		NoIntervention	175	543,000	Dominated
		SxScreen/PCR/Hospital	82	1,115,000	Dominated
		UniversalPCR/ACS	95	1,314,000	Dominated
		Hybrid/ACS	71	1,339,000	71,000
		UniversalPCR/Hospital	95	1,918,000	Dominated
		Hybrid/Hospital	71	2,018,000	Dominated
		UniversalPCR/TempHousing	71	39,607,000	Dominated

Abbreviations: ACS, alternate care sites; COVID-19, coronavirus disease 2019; Dominated, less clinically effective and more costly than an alternative strategy, or a combination of two alternative strategies;[14] Incr., incremental; *PCR,* polymerase chain reaction; *SxScreen,* symptom screen; *TempHousing,* temporary housing; *UniversalPCR*, universal polymerase chain reaction test for everyone; USD, United States dollars.

© 2020 Baggett TP et al. *JAMA Network Open.*

Strategies are listed in order of ascending costs, per convention of cost-effectiveness analysis.

* Costs were rounded to the nearest thousands.

© 2020 Baggett TP et al. *JAMA Network Open.*

eTable 11 cont. One-Way Sensitivity Analysis on Daily Cost of Temporary Housing

Effective reproduction number (R_e)	Daily cost of temporary housing, 2020 USD	Strategy	Cumulative infections, n	Total cost *, 2020 USD	Incr. cost per case prevented *, 2020 USD
2.6	0				
		UniversalPCR/TempHousing	379	1,274,000	NA
		SxScreen/PCR/ACS	1,239	3,267,000	Dominated
		Hybrid/ACS	985	3,628,000	Dominated
		UniversalPCR/ACS	1,681	4,143,000	Dominated
		NoIntervention	1,954	6,098,000	Dominated
		Hybrid/Hospital	967	12,202,000	Dominated
		SxScreen/PCR/Hospital	1,133	12,620,000	Dominated
		UniversalPCR/Hospital	1,679	12,914,000	Dominated
	20				
		SxScreen/PCR/ACS	1,239	3,267,000	NA
		Hybrid/ACS	985	3,628,000	1,000
		UniversalPCR/ACS	1,681	4,143,000	Dominated
		NoIntervention	1,954	6,098,000	Dominated
		UniversalPCR/TempHousing	379	6,735,000	5,000
		Hybrid/Hospital	967	12,202,000	Dominated
		SxScreen/PCR/Hospital	1,133	12,620,000	Dominated
		UniversalPCR/Hospital	1,679	12,914,000	Dominated
	40				
		SxScreen/PCR/ACS	1,239	3,267,000	NA
		Hybrid/ACS	985	3,628,000	1,000
		UniversalPCR/ACS	1,681	4,143,000	Dominated
		NoIntervention	1,954	6,098,000	Dominated
		UniversalPCR/TempHousing	379	12,196,000	14,000
		Hybrid/Hospital	967	12,202,000	Dominated
		SxScreen/PCR/Hospital	1,133	12,620,000	Dominated
		UniversalPCR/Hospital	1,679	12,914,000	Dominated

© 2020 Baggett TP et al. *JAMA Network Open.*

eTable 11 cont. One-Way Sensitivity Analysis on Daily Cost of Temporary Housing

Effective reproduction number (R_e)	Daily cost of temporary housing, 2020 USD	Strategy	Cumulative infections, n	Total cost *, 2020 USD	Incr. cost per case prevented *, 2020 USD
2.6	60				
		SxScreen/PCR/ACS	1,239	3,267,000	NA
		Hybrid/ACS	985	3,628,000	1,000
		UniversalPCR/ACS	1,681	4,143,000	Dominated
		NoIntervention	1,954	6,098,000	Dominated
		Hybrid/Hospital	967	12,202,000	Dominated
		SxScreen/PCR/Hospital	1,133	12,620,000	Dominated
		UniversalPCR/Hospital	1,679	12,914,000	Dominated
		UniversalPCR/TempHousing	379	17,657,000	23,000
	80				
		SxScreen/PCR/ACS	1,239	3,267,000	NA
		Hybrid/ACS	985	3,628,000	1,000
		UniversalPCR/ACS	1,681	4,143,000	Dominated
		NoIntervention	1,954	6,098,000	Dominated
		Hybrid/Hospital	967	12,202,000	Dominated
		SxScreen/PCR/Hospital	1,133	12,620,000	Dominated
		UniversalPCR/Hospital	1,679	12,914,000	Dominated
		UniversalPCR/TempHousing	379	23,117,000	32,000
	100				
		SxScreen/PCR/ACS	1,239	3,267,000	NA
		Hybrid/ACS	985	3,628,000	1,000
		UniversalPCR/ACS	1,681	4,143,000	Dominated
		NoIntervention	1,954	6,098,000	Dominated
		Hybrid/Hospital	967	12,202,000	Dominated
		SxScreen/PCR/Hospital	1,133	12,620,000	Dominated
		UniversalPCR/Hospital	1,679	12,914,000	Dominated
		UniversalPCR/TempHousing	379	28,578,000	41,000

© 2020 Baggett TP et al. *JAMA Network Open.*

eTable 11 cont. One-Way Sensitivity Analysis on Daily Cost of Temporary Housing

**Effective reproduction number (R_e)**	**Daily cost of temporary housing, 2020 USD**	**Strategy**	**Cumulative infections, n**	**Total cost *, 2020 USD**	**Incr. cost per case prevented *, 2020 USD**
2.6	120				
		SxScreen/PCR/ACS	1,239	3,267,000	NA
		Hybrid/ACS	985	3,628,000	1,000
		UniversalPCR/ACS	1,681	4,143,000	Dominated
		NoIntervention	1,954	6,098,000	Dominated
		Hybrid/Hospital	967	12,202,000	Dominated
		SxScreen/PCR/Hospital	1,133	12,620,000	Dominated
		UniversalPCR/Hospital	1,679	12,914,000	Dominated
		UniversalPCR/TempHousing	379	34,039,000	50,000
	141				
	base case	*SxScreen/PCR/ACS*	1,239	3,267,000	NA
		Hybrid/ACS	985	3,628,000	1,000
		UniversalPCR/ACS	1,681	4,143,000	Dominated
		NoIntervention	1,954	6,098,000	Dominated
		Hybrid/Hospital	967	12,202,000	Dominated
		SxScreen/PCR/Hospital	1,133	12,620,000	Dominated
		UniversalPCR/Hospital	1,679	12,914,000	Dominated
		UniversalPCR/TempHousing	376	39,119,000	58,000
	160				
		SxScreen/PCR/ACS	1,239	3,267,000	NA
		Hybrid/ACS	985	3,628,000	1,000
		UniversalPCR/ACS	1,681	4,143,000	Dominated
		NoIntervention	1,954	6,098,000	Dominated
		Hybrid/Hospital	967	12,202,000	Dominated
		SxScreen/PCR/Hospital	1,133	12,620,000	Dominated
		UniversalPCR/Hospital	1,679	12,914,000	Dominated
		UniversalPCR/TempHousing	379	44,960,000	68,000

© 2020 Baggett TP et al. *JAMA Network Open.*

eTable 11 cont. One-Way Sensitivity Analysis on Daily Cost of Temporary Housing

Effective reproduction number (R_e)	Daily cost of temporary housing, 2020 USD	Strategy	Cumulative infections, n	Total cost *, 2020 USD	Incr. cost per case prevented *, 2020 USD
1.3	0				
		SxScreen/PCR/ACS	137	409,000	NA
		UniversalPCR/TempHousing	95	1,110,000	17,000
		Hybrid/ACS	103	1,325,000	Dominated
		UniversalPCR/ACS	207	1,426,000	Dominated
		NoIntervention	538	1,461,000	Dominated
		SxScreen/PCR/Hospital	125	1,604,000	Dominated
		Hybrid/Hospital	100	2,368,000	Dominated
		UniversalPCR/Hospital	207	2,631,000	Dominated
	20				
		SxScreen/PCR/ACS	137	409,000	NA
		Hybrid/ACS	103	1,325,000	27,000
		UniversalPCR/ACS	207	1,426,000	Dominated
		NoIntervention	538	1,461,000	Dominated
		SxScreen/PCR/Hospital	125	1,604,000	Dominated
		Hybrid/Hospital	100	2,368,000	348,000
		UniversalPCR/Hospital	207	2,631,000	Dominated
		UniversalPCR/TempHousing	95	6,574,000	896,000
	40				
		SxScreen/PCR/ACS	137	409,000	NA
		Hybrid/ACS	103	1,325,000	27,000
		UniversalPCR/ACS	207	1,426,000	Dominated
		NoIntervention	538	1,461,000	Dominated
		SxScreen/PCR/Hospital	125	1,604,000	Dominated
		Hybrid/Hospital	100	2,368,000	348,000
		UniversalPCR/Hospital	207	2,631,000	Dominated
		UniversalPCR/TempHousing	95	12,037,000	2,060,000

© 2020 Baggett TP et al. *JAMA Network Open.*

eTable 11 cont. One-Way Sensitivity Analysis on Daily Cost of Temporary Housing

Effective reproduction number (R_e)	Daily cost of temporary housing, 2020 USD	Strategy	Cumulative infections, n	Total cost *, 2020 USD	Incr. cost per case prevented *, 2020 USD
1.3	60				
		SxScreen/PCR/ACS	137	409,000	NA
		Hybrid/ACS	103	1,325,000	27,000
		UniversalPCR/ACS	207	1,426,000	Dominated
		NoIntervention	538	1,461,000	Dominated
		SxScreen/PCR/Hospital	125	1,604,000	Dominated
		Hybrid/Hospital	100	2,368,000	348,000
		UniversalPCR/Hospital	207	2,631,000	Dominated
		UniversalPCR/TempHousing	95	17,500,000	3,223,000
	80				
		SxScreen/PCR/ACS	137	409,000	NA
		Hybrid/ACS	103	1,325,000	27,000
		UniversalPCR/ACS	207	1,426,000	Dominated
		NoIntervention	538	1,461,000	Dominated
		SxScreen/PCR/Hospital	125	1,604,000	Dominated
		Hybrid/Hospital	100	2,368,000	348,000
		UniversalPCR/Hospital	207	2,631,000	Dominated
		UniversalPCR/TempHousing	95	22,963,000	4,387,000
	100				
		SxScreen/PCR/ACS	137	409,000	NA
		Hybrid/ACS	103	1,325,000	27,000
		UniversalPCR/ACS	207	1,426,000	Dominated
		NoIntervention	538	1,461,000	Dominated
		SxScreen/PCR/Hospital	125	1,604,000	Dominated
		Hybrid/Hospital	100	2,368,000	348,000
		UniversalPCR/Hospital	207	2,631,000	Dominated
		UniversalPCR/TempHousing	95	28,427,000	5,551,000

© 2020 Baggett TP et al. *JAMA Network Open.*

eTable 11 cont. One-Way Sensitivity Analysis on Daily Cost of Temporary Housing

Effective reproduction number (R_e)	Daily cost of temporary housing, 2020 USD	Strategy	Cumulative infections, n	Total cost *, 2020 USD	Incr. cost per case prevented *, 2020 USD
1.3	120				
		SxScreen/PCR/ACS	137	409,000	NA
		Hybrid/ACS	103	1,325,000	27,000
		UniversalPCR/ACS	207	1,426,000	Dominated
		NoIntervention	538	1,461,000	Dominated
		SxScreen/PCR/Hospital	125	1,604,000	Dominated
		Hybrid/Hospital	100	2,368,000	348,000
		UniversalPCR/Hospital	207	2,631,000	Dominated
		UniversalPCR/TempHousing	95	33,890,000	6,715,000
	141				
	base case	*SxScreen/PCR/ACS*	137	409,000	NA
		Hybrid/ACS	103	1,325,000	27,000
		UniversalPCR/ACS	207	1,426,000	Dominated
		NoIntervention	538	1,461,000	Dominated
		SxScreen/PCR/Hospital	125	1,604,000	Dominated
		Hybrid/Hospital	100	2,368,000	382,000
		UniversalPCR/Hospital	207	2,631,000	Dominated
		UniversalPCR/TempHousing	95	38,974,000	6,854,000
	160				
		SxScreen/PCR/ACS	137	409,000	NA
		Hybrid/ACS	103	1,325,000	27,000
		UniversalPCR/ACS	207	1,426,000	Dominated
		NoIntervention	538	1,461,000	Dominated
		SxScreen/PCR/Hospital	125	1,604,000	Dominated
		Hybrid/Hospital	100	2,368,000	348,000
		UniversalPCR/Hospital	207	2,631,000	Dominated
		UniversalPCR/TempHousing	95	44,816,000	9,042,000

© 2020 Baggett TP et al. *JAMA Network Open.*

eTable 11 cont. One-Way Sensitivity Analysis on Daily Cost of Temporary Housing

Effective reproduction number (R_e)	Daily cost of temporary housing, 2020 USD	Strategy	Cumulative infections, n	Total cost *, 2020 USD	Incr. cost per case prevented *, 2020 USD
0.9	0				
		SxScreen/PCR/ACS	85	264,000	NA
		NoIntervention	174	540,000	Dominated
		UniversalPCR/TempHousing	71	1,089,000	60,000
		SxScreen/PCR/Hospital	82	1,113,000	Dominated
		UniversalPCR/ACS	94	1,226,000	Dominated
		Hybrid/ACS	71	1,240,000	667,000
		UniversalPCR/Hospital	95	1,901,000	Dominated
		Hybrid/Hospital	71	2,004,000	Dominated
	20				
		SxScreen/PCR/ACS	85	264,000	NA
		NoIntervention	174	540,000	Dominated
		SxScreen/PCR/Hospital	82	1,113,000	Dominated
		UniversalPCR/ACS	94	1,226,000	Dominated
		Hybrid/ACS	71	1,240,000	70,000
		UniversalPCR/Hospital	95	1,901,000	Dominated
		Hybrid/Hospital	71	2,004,000	Dominated
		UniversalPCR/TempHousing	71	6,552,000	Dominated
	40				
		SxScreen/PCR/ACS	85	264,000	NA
		NoIntervention	174	540,000	Dominated
		SxScreen/PCR/Hospital	82	1,113,000	Dominated
		UniversalPCR/ACS	94	1,226,000	Dominated
		Hybrid/ACS	71	1,240,000	70,000
		UniversalPCR/Hospital	95	1,901,000	Dominated
		Hybrid/Hospital	71	2,004,000	Dominated
		UniversalPCR/TempHousing	71	12,016,000	Dominated

© 2020 Baggett TP et al. *JAMA Network Open.*

eTable 11 cont. One-Way Sensitivity Analysis on Daily Cost of Temporary Housing

Effective reproduction number (R_e)	Daily cost of temporary housing, 2020 USD	Strategy	Cumulative infections, n	Total cost *, 2020 USD	Incr. cost per case prevented *, 2020 USD
0.9	60				
		SxScreen/PCR/ACS	85	264,000	NA
		NoIntervention	174	540,000	Dominated
		SxScreen/PCR/Hospital	82	1,113,000	Dominated
		UniversalPCR/ACS	94	1,226,000	Dominated
		Hybrid/ACS	71	1,240,000	70,000
		UniversalPCR/Hospital	95	1,901,000	Dominated
		Hybrid/Hospital	71	2,004,000	Dominated
		UniversalPCR/TempHousing	71	17,479,000	Dominated
	80				
		SxScreen/PCR/ACS	85	264,000	NA
		NoIntervention	174	540,000	Dominated
		SxScreen/PCR/Hospital	82	1,113,000	Dominated
		UniversalPCR/ACS	94	1,226,000	Dominated
		Hybrid/ACS	71	1,240,000	70,000
		UniversalPCR/Hospital	95	1,901,000	Dominated
		Hybrid/Hospital	71	2,004,000	Dominated
		UniversalPCR/TempHousing	71	22,943,000	Dominated
	100				
		SxScreen/PCR/ACS	85	264,000	NA
		NoIntervention	174	540,000	Dominated
		SxScreen/PCR/Hospital	82	1,113,000	Dominated
		UniversalPCR/ACS	94	1,226,000	Dominated
		Hybrid/ACS	71	1,240,000	70,000
		UniversalPCR/Hospital	95	1,901,000	Dominated
		Hybrid/Hospital	71	2,004,000	Dominated
		UniversalPCR/TempHousing	71	28,406,000	Dominated

© 2020 Baggett TP et al. *JAMA Network Open.*

eTable 11 cont. One-Way Sensitivity Analysis on Daily Cost of Temporary Housing

Effective reproduction number (R_e)	Daily cost of temporary housing, 2020 USD	Strategy	Cumulative infections, n	Total cost *, 2020 USD	Incr. cost per case prevented *, 2020 USD
0.9	120				
		SxScreen/PCR/ACS	85	264,000	NA
		NoIntervention	174	540,000	Dominated
		SxScreen/PCR/Hospital	82	1,113,000	Dominated
		UniversalPCR/ACS	94	1,226,000	Dominated
		Hybrid/ACS	71	1,240,000	70,000
		UniversalPCR/Hospital	95	1,901,000	Dominated
		Hybrid/Hospital	71	2,004,000	Dominated
		UniversalPCR/TempHousing	71	33,870,000	Dominated
	141				
	base case	*SxScreen/PCR/ACS*	85	264,000	NA
		NoIntervention	174	540,000	Dominated
		SxScreen/PCR/Hospital	82	1,113,000	Dominated
		UniversalPCR/ACS	94	1,226,000	Dominated
		Hybrid/ACS	71	1,240,000	71,000
		UniversalPCR/Hospital	95	1,901,000	Dominated
		Hybrid/Hospital	71	2,004,000	Dominated
		UniversalPCR/TempHousing	71	38,954,000	Dominated
	160				
		SxScreen/PCR/ACS	85	264,000	NA
		NoIntervention	174	540,000	Dominated
		SxScreen/PCR/Hospital	82	1,113,000	Dominated
		UniversalPCR/ACS	94	1,226,000	Dominated
		Hybrid/ACS	71	1,240,000	70,000
		UniversalPCR/Hospital	95	1,901,000	Dominated
		Hybrid/Hospital	71	2,004,000	Dominated
		UniversalPCR/TempHousing	71	44,797,000	Dominated

Abbreviations: ACS, alternate care sites; COVID-19, coronavirus disease 2019; Dominated, less clinically effective and more costly than an alternative strategy, or a combination of two alternative strategies;[14] Incr., incremental; *PCR,* polymerase chain reaction; *SxScreen,* symptom screen; *TempHousing,* temporary housing; *UniversalPCR*, universal polymerase chain reaction test for everyone; USD, United States dollars.

© 2020 Baggett TP et al. *JAMA Network Open.*

Strategies are listed in order of ascending costs, per convention of cost-effectiveness analysis.

* Costs were rounded to the nearest thousands.

© 2020 Baggett TP et al. *JAMA Network Open.*

eTable 12. Two-Way Sensitivity Analysis on PCR Sensitivity and PCR Cost for the Symptom Screening, PCR, and ACS and Hybrid ACS Strategies

PCR sensitivity for people with mild/mod. illness, %	PCR test cost, 2020 USD	Strategy	Cumulative infections, n	Total cost *, 2020 USD	Incr. cost per case prevented *, 2020 USD
**Effective reproduction number (R_e) = 2.6**					
60	5	*Hybrid/ACS*	1,159	3,075,000	NA
		SxScreen/PCR/ACS	1,346	3,406,000	Dominated
	10	*Hybrid/ACS*	1,159	3,180,000	NA
		SxScreen/PCR/ACS	1,346	3,412,000	Dominated
	25	*SxScreen/PCR/ACS*	1,346	3,430,000	NA
		Hybrid/ACS	1,159	3,494,000	300
	51	*SxScreen/PCR/ACS*	1,346	3,462,000	NA
	(base case)	*Hybrid/ACS*	1,159	4,039,000	3,000
	75	*SxScreen/PCR/ACS*	1,346	3,491,000	NA
		Hybrid/ACS	1,159	4,542,000	6,000
70	5	*Hybrid/ACS*	985	2,711,000	NA
		SxScreen/PCR/ACS	1,239	3,246,000	Dominated
	10	*Hybrid/ACS*	985	2,815,000	NA
		SxScreen/PCR/ACS	1,239	3,251,000	Dominated
	25	*Hybrid/ACS*	985	3,127,000	NA
		SxScreen/PCR/ACS	1,239	3,267,000	Dominated
	51	*SxScreen/PCR/ACS*	1,239	3,267,000	NA
	(base case)	*Hybrid/ACS*	985	3,628,000	1,000
	75	*SxScreen/PCR/ACS*	1,239	3,320,000	NA
		Hybrid/ACS	985	4,168,000	3,000

© 2020 Baggett TP et al. *JAMA Network Open.*

eTable 12 cont. Two-Way Sensitivity Analysis on PCR Sensitivity and PCR Cost for the Symptom Screening, PCR, and ACS and Hybrid ACS Strategies

PCR sensitivity for people with mild/mod. illness, %	PCR test cost, 2020 USD	Strategy	Cumulative infections, n	Total cost *, 2020 USD	Incr. cost per case prevented *, 2020 USD
**Effective reproduction number (R_e) = 2.6**					
80	5	*Hybrid/ACS*	825	2,372,000	NA
		SxScreen/PCR/ACS	1,154	3,085,000	Dominated
	10	*Hybrid/ACS*	825	2,475,000	NA
		SxScreen/PCR/ACS	1,154	3,090,000	Dominated
	25	*Hybrid/ACS*	825	2,786,000	NA
		SxScreen/PCR/ACS	1,154	3,104,000	Dominated
	51	*SxScreen/PCR/ACS*	1,154	3,128,000	NA
	(base case)	*Hybrid/ACS*	825	3,324,000	1,000
	75	*SxScreen/PCR/ACS*	1,154	3,150,000	NA
		Hybrid/ACS	825	3,820,000	2,000
90	5	*Hybrid/ACS*	668	2,019,000	NA
		SxScreen/PCR/ACS	1,067	2,903,000	Dominated
	10	*Hybrid/ACS*	668	2,122,000	NA
		SxScreen/PCR/ACS	1,067	2,907,000	Dominated
	25	*Hybrid/ACS*	668	2,431,000	NA
		SxScreen/PCR/ACS	1,067	2,919,000	Dominated
	51	*SxScreen/PCR/ACS*	1,067	2,940,000	NA
	(base case)	*Hybrid/ACS*	668	2,966,000	100
	75	*SxScreen/PCR/ACS*	1,067	2,960,000	NA
		Hybrid/ACS	668	3,459,000	1,000

© 2020 Baggett TP et al. *JAMA Network Open.*

eTable 12 cont. Two-Way Sensitivity Analysis on PCR Sensitivity and PCR Cost for the Symptom Screening, PCR, and ACS and Hybrid ACS Strategies

PCR sensitivity for people with mild/mod. illness, %	PCR test cost, 2020 USD	Strategy	Cumulative infections, n	Total cost *, 2020 USD	Incr. cost per case prevented *, 2020 USD
**Effective reproduction number (R_e) = 1.3**					
60	5	*SxScreen/PCR/ACS*	148	413,000	NA
		Hybrid/ACS	116	433,000	1,000
	10	*SxScreen/PCR/ACS*	148	414,000	NA
		Hybrid/ACS	116	534,000	4,000
	25	*SxScreen/PCR/ACS*	148	417,000	NA
		Hybrid/ACS	116	839,000	13,000
	51	*SxScreen/PCR/ACS*	148	420,000	NA
	(base case)	*Hybrid/ACS*	116	1,366,000	30,000
	75	*SxScreen/PCR/ACS*	148	425,000	NA
		Hybrid/ACS	116	1,852,000	45,000
70	5	*SxScreen/PCR/ACS*	137	402,000	NA
		Hybrid/ACS	103	409,000	200
	10	*SxScreen/PCR/ACS*	137	403,000	NA
		Hybrid/ACS	103	510,000	3,000
	25	*SxScreen/PCR/ACS*	137	405,000	NA
		Hybrid/ACS	103	814,000	12,000
	51	*SxScreen/PCR/ACS*	137	409,000	NA
	(base case)	*Hybrid/ACS*	103	1,341,000	27,000
	75	*SxScreen/PCR/ACS*	137	413,000	NA
		Hybrid/ACS	103	1,827,000	41,000

© 2020 Baggett TP et al. *JAMA Network Open.*

eTable 12 cont. Two-Way Sensitivity Analysis on PCR Sensitivity and PCR Cost for the Symptom Screening, PCR, and ACS and Hybrid ACS Strategies

PCR sensitivity for people with mild/mod. illness, %	PCR test cost, 2020 USD	Strategy	Cumulative infections, n	Total cost *, 2020 USD	Incr. cost per case prevented *, 2020 USD
**Effective reproduction number (R_e) = 1.3**					
80	5	*SxScreen/PCR/ACS*	127	386,000	NA
		Hybrid/ACS	92	400,000	400
	10	*SxScreen/PCR/ACS*	127	386,000	NA
		Hybrid/ACS	92	501,000	3,000
	25	*SxScreen/PCR/ACS*	127	388,000	NA
		Hybrid/ACS	92	805,000	12,000
	51	*SxScreen/PCR/ACS*	127	392,000	NA
	(base case)	*Hybrid/ACS*	92	1,331,000	27,000
	75	*SxScreen/PCR/ACS*	127	395,000	NA
		Hybrid/ACS	92	1,817,000	41,000
90	5	*SxScreen/PCR/ACS*	121	378,000	NA
		Hybrid/ACS	85	388,000	300
	10	*SxScreen/PCR/ACS*	121	378,000	NA
		Hybrid/ACS	85	489,000	3,000
	25	*SxScreen/PCR/ACS*	121	380,000	NA
		Hybrid/ACS	85	793,000	12,000
	51	*SxScreen/PCR/ACS*	121	383,000	NA
	(base case)	*Hybrid/ACS*	85	1,319,000	27,000
	75	*SxScreen/PCR/ACS*	121	386,000	NA
		Hybrid/ACS	85	1,805,000	40,000

© 2020 Baggett TP et al. *JAMA Network Open.*

eTable 12 cont. Two-Way Sensitivity Analysis on PCR Sensitivity and PCR Cost for the Symptom Screening, PCR, and ACS and Hybrid ACS Strategies

PCR sensitivity for people with mild/mod. illness, %	PCR test cost, 2020 USD	Strategy	Cumulative infections, n	Total cost *, 2020 USD	Incr. cost per case prevented *, 2020 USD
**Effective reproduction number (R_e) = 0.9**					
60	5	*SxScreen/PCR/ACS*	87	263,000	NA
		Hybrid/ACS	76	330,000	6,000
	10	*SxScreen/PCR/ACS*	87	264,000	NA
		Hybrid/ACS	76	432,000	15,000
	25	*SxScreen/PCR/ACS*	87	266,000	NA
		Hybrid/ACS	76	735,000	41,000
	51	*SxScreen/PCR/ACS*	87	272,200	NA
	(base case)	*Hybrid/ACS*	76	1,261,000	88,000
	75	*SxScreen/PCR/ACS*	87	271,000	NA
		Hybrid/ACS	76	1,747,000	128,000
70	5	*SxScreen/PCR/ACS*	85	259,000	NA
		Hybrid/ACS	71	325,000	5,000
	10	*SxScreen/PCR/ACS*	85	260,000	NA
		Hybrid/ACS	71	426,000	12,000
	25	*SxScreen/PCR/ACS*	85	262,000	NA
		Hybrid/ACS	71	730,000	34,000
	51	*SxScreen/PCR/ACS*	85	264,000	NA
	(base case)	*Hybrid/ACS*	71	1,256,000	72,000
	75	*SxScreen/PCR/ACS*	85	267,000	NA
		Hybrid/ACS	71	1,742,000	107,000

© 2020 Baggett TP et al. *JAMA Network Open.*

eTable 12 cont. Two-Way Sensitivity Analysis on PCR Sensitivity and PCR Cost for the Symptom Screening, PCR, and ACS and Hybrid ACS Strategies

**PCR sensitivity for people with mild/mod. illness, %**	**PCR test cost, 2020 USD**	**Strategy**	**Cumulative infections, n**	**Total cost *, 2020 USD**	**Incr. cost per case prevented *, 2020 USD**
**Effective reproduction number (R_e) = 0.9**					
80	5	*SxScreen/PCR/ACS*	82	255,000	NA
		Hybrid/ACS	68	326,000	5,000
	10	*SxScreen/PCR/ACS*	82	255,000	NA
		Hybrid/ACS	68	427,000	13,000
	25	*SxScreen/PCR/ACS*	82	257,000	NA
		Hybrid/ACS	68	731,000	35,000
	51	*SxScreen/PCR/ACS*	82	259,000	NA
	(base case)	*Hybrid/ACS*	68	1,257,000	74,000
	75	*SxScreen/PCR/ACS*	82	262,000	NA
		Hybrid/ACS	68	1,742,000	109,000
90	5	*SxScreen/PCR/ACS*	80	259,000	NA
		Hybrid/ACS	65	322,000	4,000
	10	*SxScreen/PCR/ACS*	80	259,000	NA
		Hybrid/ACS	65	423,000	11,000
	25	*SxScreen/PCR/ACS*	80	261,000	NA
		Hybrid/ACS	65	727,000	32,000
	51	*SxScreen/PCR/ACS*	80	263,000	NA
	(base case)	*Hybrid/ACS*	65	1,252,000	67,000
	75	*SxScreen/PCR/ACS*	80	265,000	NA
		Hybrid/ACS	65	1,738,000	100,000

Abbreviations: ACS, alternate care sites; COVID-19, coronavirus disease 2019; Dominated, less clinically effective and more costly than an alternative strategy, or a combination of two alternative strategies;[14] Incr., incremental; mild/mod., mild/moderate; *PCR,* polymerase chain reaction; *TempHousing,* temporary housing; *UniversalPCR*, universal polymerase chain reaction test for everyone; USD, United States dollars; *SxScreen,* symptom screen.

Strategies are listed in order of ascending costs, per convention of cost-effectiveness analysis.

* Costs were rounded to the nearest thousands.

© 2020 Baggett TP et al. *JAMA Network Open.*

eTable 13. Two-Way Sensitivity Analysis on PCR Testing Frequency and PCR Cost for the Symptom Screening, PCR, and ACS and Hybrid ACS Strategies

PCR testing frequency	PCR test cost, 2020 USD	Strategy	Cumulative infections, n	Total cost *, 2020 USD	Incr. cost per case prevented *, 2020 USD
**Effective reproduction number (R_e) = 2.6**					
Every 30 days	5	*Hybrid/ACS*	1,179	3,034,000	NA
		SxScreen/PCR/ACS	1,244	3,246,000	Dominated
	10	*Hybrid/ACS*	1,179	3,095,000	NA
		SxScreen/PCR/ACS	1,244	3,251,000	Dominated
	25	*SxScreen/PCR/ACS*	1,244	3,267,000	NA
		Hybrid/ACS	1,179	3,277,000	200
	51	*SxScreen/PCR/ACS*	1,244	3,295,000	NA
	(base case)	*Hybrid/ACS*	1,179	3,594,000	5,000
	75	*SxScreen/PCR/ACS*	1,244	3,320,000	NA
		Hybrid/ACS	1,179	3,886,000	9,000
Every 14 days	5	*Hybrid/ACS*	989	2,711,000	NA
(base case)		*SxScreen/PCR/ACS*	1,244	3,246,000	Dominated
	10	*Hybrid/ACS*	989	2,815,000	NA
		SxScreen/PCR/ACS	1,244	3,251,000	Dominated
	25	*Hybrid/ACS*	989	3,127,000	NA
		SxScreen/PCR/ACS	1,244	3,267,000	Dominated
	51	*SxScreen/PCR/ACS*	1,244	3,295,000	NA
	(base case)	*Hybrid/ACS*	989	3,669,000	1,000
	75	*SxScreen/PCR/ACS*	1,244	3,320,000	NA
		Hybrid/ACS	989	4,168,000	3,000

© 2020 Baggett TP et al. *JAMA Network Open.*

eTable 13 cont. Two-Way Sensitivity Analysis on PCR Testing Frequency and PCR Cost for the Symptom Screening, PCR, and ACS and Hybrid ACS Strategies

PCR testing frequency	PCR test cost, 2020 USD	Strategy	Cumulative infections, n	Total cost *, 2020 USD	Incr. cost per case prevented *, 2020 USD
**Effective reproduction number (R_e) = 2.6**					
Every 7 days	5	*Hybrid/ACS*	700	2,171,000	NA
		SxScreen/PCR/ACS	1,244	3,246,000	Dominated
	10	*Hybrid/ACS*	700	2,350,000	NA
		SxScreen/PCR/ACS	1,244	3,251,000	Dominated
	25	*Hybrid/ACS*	700	2,887,000	NA
		SxScreen/PCR/ACS	1,244	3,267,000	Dominated
	51	*SxScreen/PCR/ACS*	1,244	3,295,000	NA
	(base case)	*Hybrid/ACS*	700	3,818,000	1,000
	75	*SxScreen/PCR/ACS*	1,244	3,320,000	NA
		Hybrid/ACS	700	4,677,000	2,000
Every 3 days	5	*Hybrid/ACS*	334	1,438,000	NA
		SxScreen/PCR/ACS	1,244	3,246,000	Dominated
	10	*Hybrid/ACS*	334	1,783,000	NA
		SxScreen/PCR/ACS	1,244	3,251,000	Dominated
	25	*Hybrid/ACS*	334	2,817,000	NA
		SxScreen/PCR/ACS	1,244	3,267,000	Dominated
	51	*SxScreen/PCR/ACS*	1,244	3,295,000	NA
	(base case)	*Hybrid/ACS*	334	4,609,000	1,000
	75	*SxScreen/PCR/ACS*	1,244	3,320,000	NA
		Hybrid/ACS	334	6,263,000	3,000

© 2020 Baggett TP et al. *JAMA Network Open.*

eTable 13 cont. Two-Way Sensitivity Analysis on PCR Testing Frequency and PCR Cost for the Symptom Screening, PCR, and ACS and Hybrid ACS Strategies

PCR testing frequency	PCR test cost, 2020 USD	Strategy	Cumulative infections, n	Total cost *, 2020 USD	Incr. cost per case prevented *, 2020 USD
**Effective reproduction number (R_e) = 1.3**					
Every 30 days	5	*Hybrid/ACS*	115	387,000	NA
		SxScreen/PCR/ACS	137	402,000	Dominated
	10	*SxScreen/PCR/ACS*	137	403,000	NA
		Hybrid/ACS	115	442,000	2,000
	25	*SxScreen/PCR/ACS*	137	405,000	NA
		Hybrid/ACS	115	609,000	9,000
	51	*SxScreen/PCR/ACS*	137	409,000	NA
	(base case)	*Hybrid/ACS*	115	896,000	22,000
	75	*SxScreen/PCR/ACS*	137	413,000	NA
		Hybrid/ACS	115	1,162,000	34,000
Every 14 days	5	*SxScreen/PCR/ACS*	137	402,000	NA
(base case)		*Hybrid/ACS*	103	409,000	200
	10	*SxScreen/PCR/ACS*	137	403,000	NA
		Hybrid/ACS	103	510,000	3,000
	25	*SxScreen/PCR/ACS*	137	405,000	NA
		Hybrid/ACS	103	814,000	12,000
	51	*SxScreen/PCR/ACS*	137	409,000	NA
	(base case)	*Hybrid/ACS*	103	1,341,000	27,000
	75	*SxScreen/PCR/ACS*	137	413,000	NA
		Hybrid/ACS	103	1,827,000	41,000

© 2020 Baggett TP et al. *JAMA Network Open.*

eTable 13 cont. Two-Way Sensitivity Analysis on PCR Testing Frequency and PCR Cost for the Symptom Screening, PCR, and ACS and Hybrid ACS Strategies

**PCR testing frequency**	**PCR test cost, 2020 USD**	**Strategy**	**Cumulative infections, n**	**Total cost *, 2020 USD**	**Incr. cost per case prevented *, 2020 USD**
**Effective reproduction number (R_e) = 1.3**					
Every 7 days	5	*SxScreen/PCR/ACS*	137	402,000	NA
		Hybrid/ACS	91	473,000	2,000
	10	*SxScreen/PCR/ACS*	137	403,000	NA
		Hybrid/ACS	91	653,000	5,000
	25	*SxScreen/PCR/ACS*	137	405,000	NA
		Hybrid/ACS	91	1,191,000	17,000
	51	*SxScreen/PCR/ACS*	137	409,000	NA
	(base case)	*Hybrid/ACS*	91	2,123,000	37,000
	75	*SxScreen/PCR/ACS*	137	413,000	NA
		Hybrid/ACS	91	2,984,000	56,000
Every 3 days	5	*SxScreen/PCR/ACS*	137	402,000	NA
		Hybrid/ACS	79	625,000	4,000
	10	*SxScreen/PCR/ACS*	137	403,000	NA
		Hybrid/ACS	79	972,000	10,000
	25	*SxScreen/PCR/ACS*	137	405,000	NA
		Hybrid/ACS	79	2,013,000	28,000
	51	*SxScreen/PCR/ACS*	137	409,000	NA
	(base case)	*Hybrid/ACS*	79	3,817,000	59,000
	75	*SxScreen/PCR/ACS*	137	413,000	NA
		Hybrid/ACS	79	5,483,000	88,000

© 2020 Baggett TP et al. *JAMA Network Open.*

eTable 13 cont. Two-Way Sensitivity Analysis on PCR Testing Frequency and PCR Cost for the Symptom Screening, PCR, and ACS and Hybrid ACS Strategies

**PCR testing frequency**	**PCR test cost, 2020 USD**	**Strategy**	**Cumulative infections, n**	**Total cost *, 2020 USD**	**Incr. cost per case prevented *, 2020 USD**
**Effective reproduction number (R_e) = 0.9**					
Every 30 days	5	*SxScreen/PCR/ACS*	85	259,000	NA
		Hybrid/ACS	74	279,000	2,000
	10	*SxScreen/PCR/ACS*	85	260,000	NA
		Hybrid/ACS	74	334,000	7,000
	25	*SxScreen/PCR/ACS*	85	262,000	NA
		Hybrid/ACS	74	499,000	21,000
	51	*SxScreen/PCR/ACS*	85	264,000	NA
	(base case)	*Hybrid/ACS*	74	785,000	46,000
	75	*SxScreen/PCR/ACS*	85	267,000	NA
		Hybrid/ACS	74	1,050,000	69,000
Every 14 days	5	*SxScreen/PCR/ACS*	85	259,000	NA
(base case)		*Hybrid/ACS*	71	325,000	5,000
	10	*SxScreen/PCR/ACS*	85	260,000	NA
		Hybrid/ACS	71	426,000	12,000
	25	*SxScreen/PCR/ACS*	85	262,000	NA
		Hybrid/ACS	71	730,000	34,000
	51	*SxScreen/PCR/ACS*	85	264,000	NA
	(base case)	*Hybrid/ACS*	71	1,256,000	72,000
	75	*SxScreen/PCR/ACS*	85	267,000	NA
		Hybrid/ACS	71	1,742,000	107,000

© 2020 Baggett TP et al. *JAMA Network Open.*

eTable 13 cont. Two-Way Sensitivity Analysis on PCR Testing Frequency and PCR Cost for the Symptom Screening, PCR, and ACS and Hybrid ACS Strategies

PCR testing frequency	PCR test cost, 2020 USD	Strategy	Cumulative infections, n	Total cost *, 2020 USD	Incr. cost per case prevented *, 2020 USD
**Effective reproduction number (R_e) = 0.9**					
Every 7 days	5	*SxScreen/PCR/ACS*	85	259,000	NA
		Hybrid/ACS	68	406,000	8,000
	10	*SxScreen/PCR/ACS*	85	260,000	NA
		Hybrid/ACS	68	585,000	19,000
	25	*SxScreen/PCR/ACS*	85	262,000	NA
		Hybrid/ACS	68	1,124,000	49,000
	51	*SxScreen/PCR/ACS*	85	264,000	NA
	(base case)	*Hybrid/ACS*	68	2,057,000	103,000
	75	*SxScreen/PCR/ACS*	85	267,000	NA
		Hybrid/ACS	68	2,918,000	152,000
Every 3 days	5	*SxScreen/PCR/ACS*	85	259,000	NA
		Hybrid/ACS	64	569,000	15,000
	10	*SxScreen/PCR/ACS*	85	260,000	NA
		Hybrid/ACS	64	916,000	31,000
	25	*SxScreen/PCR/ACS*	85	262,000	NA
		Hybrid/ACS	64	1,958,000	80,000
	51	*SxScreen/PCR/ACS*	85	264,000	NA
	(base case)	*Hybrid/ACS*	64	3,763,000	164,000
	75	*SxScreen/PCR/ACS*	85	267,000	NA
		Hybrid/ACS	64	5,429,000	242,000

Abbreviations: ACS, alternate care sites; COVID-19, coronavirus disease 2019; Dominated, less clinically effective and more costly than an alternative strategy, or a combination of two alternative strategies;[14] Incr., incremental; *PCR,* polymerase chain reaction; *SxScreen,* symptom screen; *TempHousing,* temporary housing; *UniversalPCR*, universal polymerase chain reaction test for everyone; USD, United States dollars.

Strategies are listed in order of ascending costs, per convention of cost-effectiveness analysis.

* Costs were rounded to the nearest thousands.

© 2020 Baggett TP et al. *JAMA Network Open.*

eTable 14. Results of an Analysis of Management Strategies for People Experiencing Sheltered Homelessness During the COVID-19 Pandemic for a Cohort of 1000 Adults Experiencing Sheltered Homelessness

**Strategy**	**Cumulative infections, n**	**Peak daily hospital bed use, n**	**Hospital costs *, 2020 USD**	**ACS costs *, 2020 USD**	**Total costs *, 2020 USD**	**Costs compared with *NoIntervention* *, 2020 USD**	**Incr. cost per case prevented *, 2020 USD**
**Effective reproduction number (R_e) = 2.6**							
SxScreen/PCR/ACS	549	2	343,000	1,080,000	1,447,000	- 1,254,000	NA
Hybrid/ACS	436	2	266,000	878,000	1,607,000	- 1,094,000	1,000
UniversalPCR/ACS	744	4	499,000	885,000	1,835,000	- 866,000	Dominated
NoIntervention	865	28	2,692,000	NA	2,701,000	NA	Dominated
Hybrid/Hospital	428	35	4,941,000	NA	5,404,000	+ 2,703,000	Dominated
SxScreen/PCR/Hospital	502	41	5,567,000	NA	5,589,000	+ 2,888,000	Dominated
UniversalPCR/Hospital	743	50	5,269,000	NA	5,719,000	+ 3,018,000	Dominated
UniversalPCR/TempHousing	167	1	107,000	NA	17,325,000	+ 14,624,000	58,000
**Effective reproduction number (R_e) = 1.3**							
SxScreen/PCR/ACS	61	0	43,000	135,000	181,000	- 466,000	NA
Hybrid/ACS	46	0	30,000	106,000	587,000	- 60,000	27,000
UniversalPCR/ACS	92	0	61,000	122,000	632,000	- 15,000	Dominated
NoIntervention	238	4	645,000	NA	647,000	NA	Dominated
SxScreen/PCR/Hospital	56	10	707,000	NA	710,000	+ 63,000	Dominated
Hybrid/Hospital	44	10	30,000	NA	1,049,000	+ 402,000	382,000
UniversalPCR/Hospital	92	9	716,000	NA	1,165,000	+ 518,000	Dominated
UniversalPCR/TempHousing	42	4	35,000	NA	17,260,000	+ 16,613,000	6,853,000

© 2020 Baggett TP et al. *JAMA Network Open.*

eTable 14 cont. Results of an Analysis of Management Strategies for People Experiencing Sheltered Homelessness During the COVID-19 Pandemic for a Cohort of 1000 Adults Experiencing Sheltered Homelessness

**Strategy**	**Cumulative infections, n**	**Peak daily hospital bed use, n**	**Hospital costs *, 2020 USD**	**ACS costs *, 2020 USD**	**Total costs *, 2020 USD**	**Costs compared with *NoIntervention* *, 2020 USD**	**Incr. cost per case prevented *, 2020 USD**
**Effective reproduction number (R_e) = 0.9**							
SxScreen/PCR/ACS	38	0	27,000	88,000	117,000	- 426,000	NA
NoIntervention	77	2	238,000	NA	239,000	- 304,000	Dominated
SxScreen/PCR/Hospital	36	9	491,000	NA	493,000	- 50,000	Dominated
UniversalPCR/ACS	42	0	27,000	66,000	543,000	NA	Dominated
Hybrid/ACS	32	0	23,000	76,000	549,000	+ 6,000	71,000
UniversalPCR/Hospital	42	9	393,000	NA	842,000	+ 299,000	Dominated
Hybrid/Hospital	31	10	437,000	NA	888,000	+ 345,000	Dominated
UniversalPCR/TempHousing	31	0	26,000	NA	17,252,000	+ 16,709,000	Dominated

Abbreviations: ACS, alternate care sites; Dominated, less clinically effective and more costly than an alternative strategy, or with a higher incremental cost-effectiveness ratio than another strategy;[14] Incr., incremental; *SxScreen*, symptom screen; *TempHousing*, temporary housing; *UniversalPCR*, polymerase chain reaction test for everyone; USD, United States dollars.

Strategies are listed in order of ascending costs, per convention of cost-effectiveness analysis. City-specific population sizes of sheltered homeless adults are as follows: 2,258 in Boston, 1,343 in Houston, 2,053 in Chicago, 2,770 in Philadelphia, 3,556 in Seattle, 6,945 in Los Angeles, and 31,805 in New York. [15]

* Costs were rounded to the nearest thousands.

© 2020 Baggett TP et al. *JAMA Network Open.*

eTable 15. Total Infections and Component Costs of Different Management Strategies for Adults Experiencing Sheltered Homelessness in Boston During the COVID-19 Pandemic at 4 Months

Strategy	Cumulative infections, n	ICU bed costs, * 2020 USD	Hospital (non-ICU) bed costs, * 2020 USD	Test costs, * 2020 USD	ACS costs, * 2020 USD	Temporary housing costs, * 2020 USD	Total costs, * 2020 USD
**Effective reproduction number (R_e) = 2.6**							
SxScreen/PCR/ACS	1,239	349,000	425,000	54,000	2,439,000	NA	3,267,000
Hybrid/ACS	985	270,000	330,000	1,046,000	1,982,000	NA	3,628,000
UniversalPCR/ACS	1,681	517,000	610,000	1,017,000	1,999,000	NA	4,143,000
NoIntervention	1,954	621,000	5,458,000	19,000	NA	NA	6,098,000
Hybrid/Hospital	967	275,000	10,882,000	1,045,000	NA	NA	12,202,000
SxScreen/PCR/Hospital	1,133	318,000	12,253,000	49,000	NA	NA	12,620,000
UniversalPCR/Hospital	1,679	501,000	11,396,000	1,017,000	NA	NA	12,914,000
UniversalPCR/TempHousing	376	115,000	127,000	1,015,000	NA	37,862,000	39,119,000
**Effective reproduction number (R_e) = 1.3**							
SxScreen/PCR/ACS	137	46,000	51,000	8,000	304,000	NA	409,000
Hybrid/ACS	103	30,000	37,000	1,018,000	240,000	NA	1,325,000
UniversalPCR/ACS	207	63,000	74,000	1,014,000	275,000	NA	1,426,000
NoIntervention	538	145,000	1,311,000	5,000	NA	NA	1,461,000
SxScreen/PCR/Hospital	125	39,000	1,558,000	7,000	NA	NA	1,604,000
Hybrid/Hospital	100	31,000	1,319,000	1,018,000	NA	NA	2,368,000
UniversalPCR/Hospital	207	62,000	1,555,000	1,014,000	NA	NA	2,631,000
UniversalPCR/TempHousing	95	41,000	38,000	1,016,000	NA	37,879,000	38,974,000

© 2020 Baggett TP et al. *JAMA Network Open.*

eTable 15. cont. Total Infections and Component Costs of Different Management Strategies for Adults Experiencing Sheltered Homelessness in Boston During the COVID-19 Pandemic at 4 Months

Strategy	Cumulative infections, n	ICU bed costs, * 2020 USD	Hospital (non-ICU) bed costs, * 2020 USD	Test costs, * 2020 USD	ACS costs, * 2020 USD	Temporary housing costs, * 2020 USD	Total costs, * 2020 USD
**Effective reproduction number (R_e) = 0.9**							
SxScreen/PCR/ACS	85	28,000	33,000	5,000	198,000	NA	264,000
NoIntervention	174	50,000	488,000	2,000	NA	NA	540,000
SxScreen/PCR/Hospital	82	31,000	1,077,000	5,000	NA	NA	1,113,000
UniversalPCR/ACS	94	28,000	34,000	1,014,000	150,000	NA	1,226,000
Hybrid/ACS	71	25,000	26,000	1,017,000	172,000	NA	1,240,000
UniversalPCR/Hospital	95	31,000	856,000	1,014,000	NA	NA	1,901,000
Hybrid/Hospital	71	24,000	963,000	1,017,000	NA	NA	2,004,000
UniversalPCR/TempHousing	71	28,000	30,000	1,015,000	NA	37,881,000	38,954,000

Abbreviations: ACS, alternate care sites; COVID-19, coronavirus disease 2019; ICU, intensive care units; *PCR,* polymerase chain reaction; *UniversalPCR*, universal polymerase chain reaction test for everyone; *SxScreen,* symptom screen; *TempHousing,* temporary housing; USD, United States dollars.

Strategies are listed in order of ascending costs, per convention of cost-effectiveness analysis.

* All costs were rounded to the nearest thousands.

© 2020 Baggett TP et al. *JAMA Network Open.*

eTable 16. One-Way Sensitivity Analysis on PCR Testing Frequency for the Hybrid ACS Strategy

**Strategy**	**Cumulative infections, n**	**Incr. infections prevented, n**	**Total cost *, 2020 USD**	**Cost compared with *NoIntervention* *, 2020 USD**	**Incr. cost per case prevented *, 2020 USD**
**Effective reproduction number (R_e) = 2.6**					
SxScreen/PCR/ACS	1,239	NA	3,267,000	- 2,831,000	NA
Hybrid/ACS - PCR once every 30 days	1,189	50	3,587,000	- 2,511,000	Dominated
Hybrid/ACS - PCR once every 14 days	985	205	3,628,000	- 2,470,000	Dominated
Hybrid/ACS - PCR once every 7 days	696	289	3,849,000	- 2,249,000	1,000
Hybrid/ACS - PCR once every 3 days	340	356	4,633,000	- 1,465,000	2,000
NoIntervention	1,954	- 1,614	6,098,000	NA	Dominated
**Effective reproduction number (R_e) = 1.3**					
SxScreen/PCR/ACS	137	NA	409,000	- 1,052,000	NA
Hybrid/ACS - PCR once every 30 days	114	24	891,000	- 570,000	20,000
Hybrid/ACS - PCR once every 14 days	103	10	1,325,000	- 136,000	41,000
NoIntervention	538	- 435	1,461,000	NA	Dominated
Hybrid/ACS - PCR once every 7 days	92	446	2,123,000	+ 662,000	72,000
Hybrid/ACS - PCR once every 3 days	81	11	3,808,000	+ 2,347,000	153,000
**Effective reproduction number (R_e) = 0.9**					
SxScreen/PCR/ACS	85	NA	264,000	- 276,000	NA
NoIntervention	174	- 89	540,000	NA	Dominated
Hybrid/ACS - PCR once every 30 days	76	98	785,000	+ 245,000	60,000
Hybrid/ACS - PCR once every 14 days	71	5	1,240,000	+ 700,000	88,000
Hybrid/ACS - PCR once every 7 days	70	1	2,061,000	+ 1,521,000	Dominated
Hybrid/ACS - PCR once every 3 days	66	4	3,772,000	+ 3,232,000	484,000

Abbreviations: ACS, alternate care sites; Dominated, less clinically effective and more costly than an alternative strategy, or a combination of two alternative strategies; [14] Incr., incremental; *PCR,* polymerase chain reaction; *SxScreen,* symptom screen; *TempHousing,* temporary housing; USD, United States dollars.

Strategies are listed in order of ascending costs, per convention of cost-effectiveness analysis.

* All costs were rounded to the nearest thousands.

© 2020 Baggett TP et al. *JAMA Network Open.*

LEGENDS TO FIGURES

eFigure1. Illustration of health states and illness paths in the CEACOV model.

CEACOV simulates individuals transitioning between the states of susceptibility to severe acute respiratory syndrome coronavirus 2 (SARS-CoV-2), infection with SARS-CoV-2 and coronavirus illness 2019 (COVID-19), recovery from COVID-19, and death. Susceptible individuals face a daily probability of infection. After acquiring SARS-CoV-2 infection, individuals may progress through the following health states: pre-infectious latency, asymptomatic infection, mild/moderate illness, severe illness, critical illness, recuperation, and recovered.

After being infected with SARS-CoV-2, a susceptible individual first transitions to the pre-infectious latency stage. Then, the individual has an age-dependent probability of progressing along one of four "paths," culminating in either asymptomatic infection, mild/moderate illness, severe illness, or critical illness. Before a reaching a more advanced illness state, individuals must first transition through intermediate states (e.g., those destined for severe illness must first pass through the asymptomatic infection state and the mild/moderate illness state).

Abbreviations: Asympt., asymptomatic; CEACOV, Clinical and Economic Analysis of COVID-19 interventions model; Mild/Mod., mild/moderate.

eFigure2. Flow diagrams of management strategies for people experiencing sheltered homelessness in Boston during the COVID-19 pandemic.

We assess eight strategies for testing and management:
1) ***NoIntervention:*** Only basic infection control practices are implemented in shelters.
2) ***SxScreen/PCR/Hospital:*** CDC-recommended symptom screening daily in shelters.[6] Screen-negative individuals remain in shelters. Screen-positive individuals are sent to the hospital for PCR testing. PCR-positive individuals remain in hospital; PCR-negative individuals return to shelter.
3) ***SxScreen/PCR/ACS:*** CDC-recommended symptom screening daily in shelters. Screen-negative individuals remain in shelters. Screen-positive individuals are sent to an ACS for people under investigation, where they undergo PCR testing and await results. PCR-positive individuals with mild/moderate illness are transferred to ACSs for confirmed COVID-19 cases. PCR-negative individuals return to shelter.
4) ***UniversalPCR/Hospital:*** Universal PCR testing every 2 weeks in shelters. Those with symptoms at the time of testing await results at the hospital; individuals without symptoms await results in shelters. PCR-negative individuals return to or stay in shelters. PCR-positive individuals, regardless of illness severity, remain in or are sent to the hospital.
5) ***UniversalPCR/ACS:*** Universal PCR testing every 2 weeks in shelters. Those with symptoms at the time of testing are sent to an ACS for people under investigation while awaiting results; individuals without symptoms await results in shelters. PCR-negative individuals return to or stay in shelters. PCR-positive individuals with mild/moderate illness are transferred to ACSs for confirmed COVID-19 cases.
6) ***UniversalPCR/TempHousing:*** All shelter residents are pre-emptively moved to temporary housing for the duration of the 4-month period. Universal PCR testing occurs every 2 weeks. PCR-positive individuals with mild/moderate illness remain in temporary housing and are transferred to the hospital if they progress to severe or critical disease.
7) ***Hybrid/Hospital:*** This includes the *SxScreen/PCR/Hospital* strategy and adds shelter-based universal PCR testing every 2 weeks for those without symptoms.
8) ***Hybrid/ACS:*** This includes the *SxScreen/PCR/ACS* strategy and adds shelter-based universal PCR testing every 2 weeks for those without symptoms.

In all 8 strategies, people with severe or critical illness are sent to the hospital. Individuals are eligible for repeat PCR testing after 5 days since their most recent negative test. See eFigure2 for details.

Abbreviations: ACS, alternate care sites; COVID, coronavirus illness 2019; *PCR,* polymerase chain reaction; *Sx Screen,* symptom screen; *TempHousing,* temporary housing; *UniversalPCR*, universal polymerase chain reaction test for everyone.

© 2020 Baggett TP et al. *JAMA Network Open.*

eFigure 1. Illustration of Health States and Illness Paths in the CEACOV Model

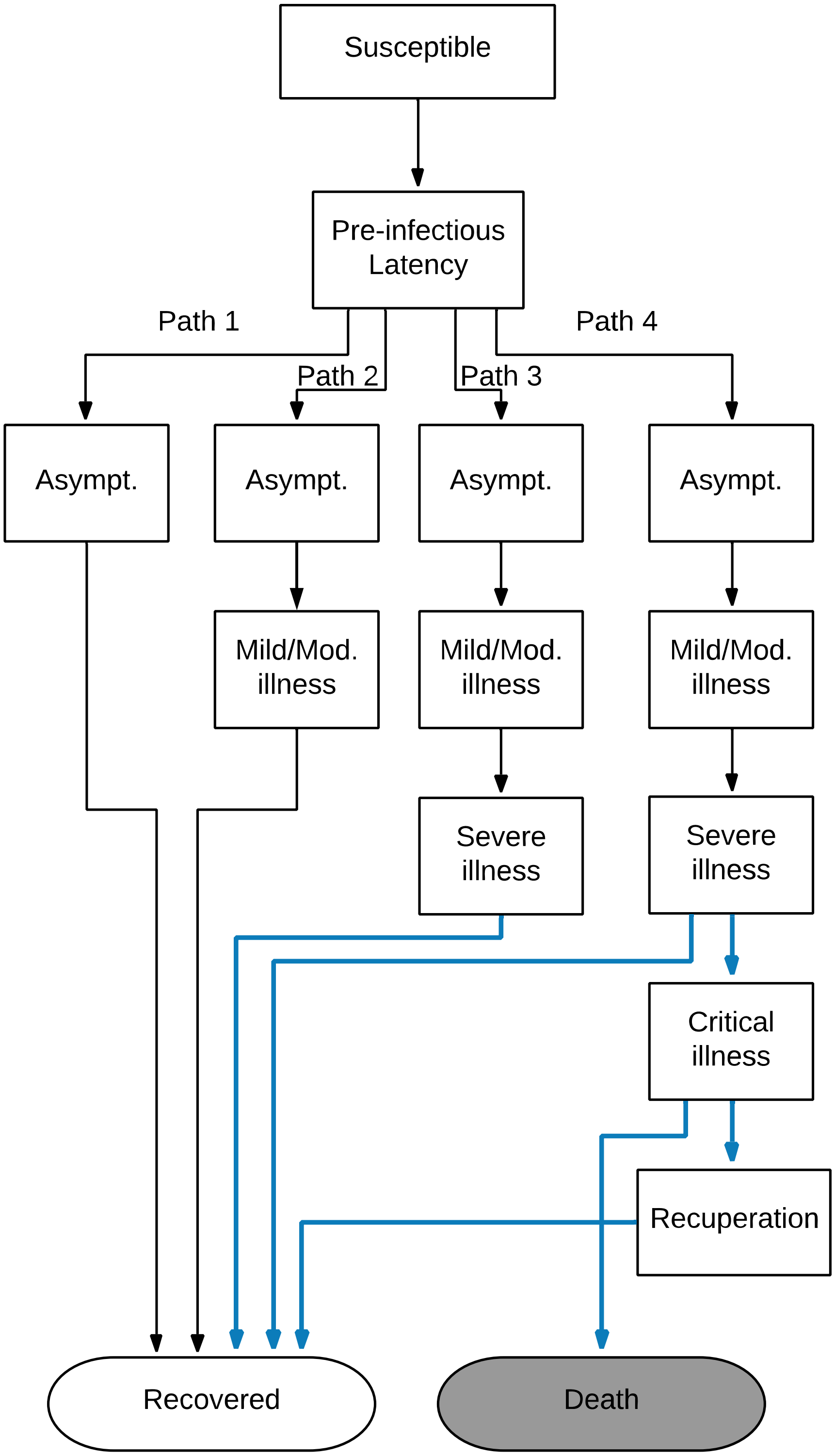

© 2020 Baggett TP et al. *JAMA Network Open.*

eFigure 2. Flow Diagrams of Management Strategies for People Experiencing Sheltered Homelessness in Boston During the COVID-19 Pandemic

1. ***NoIntervention***

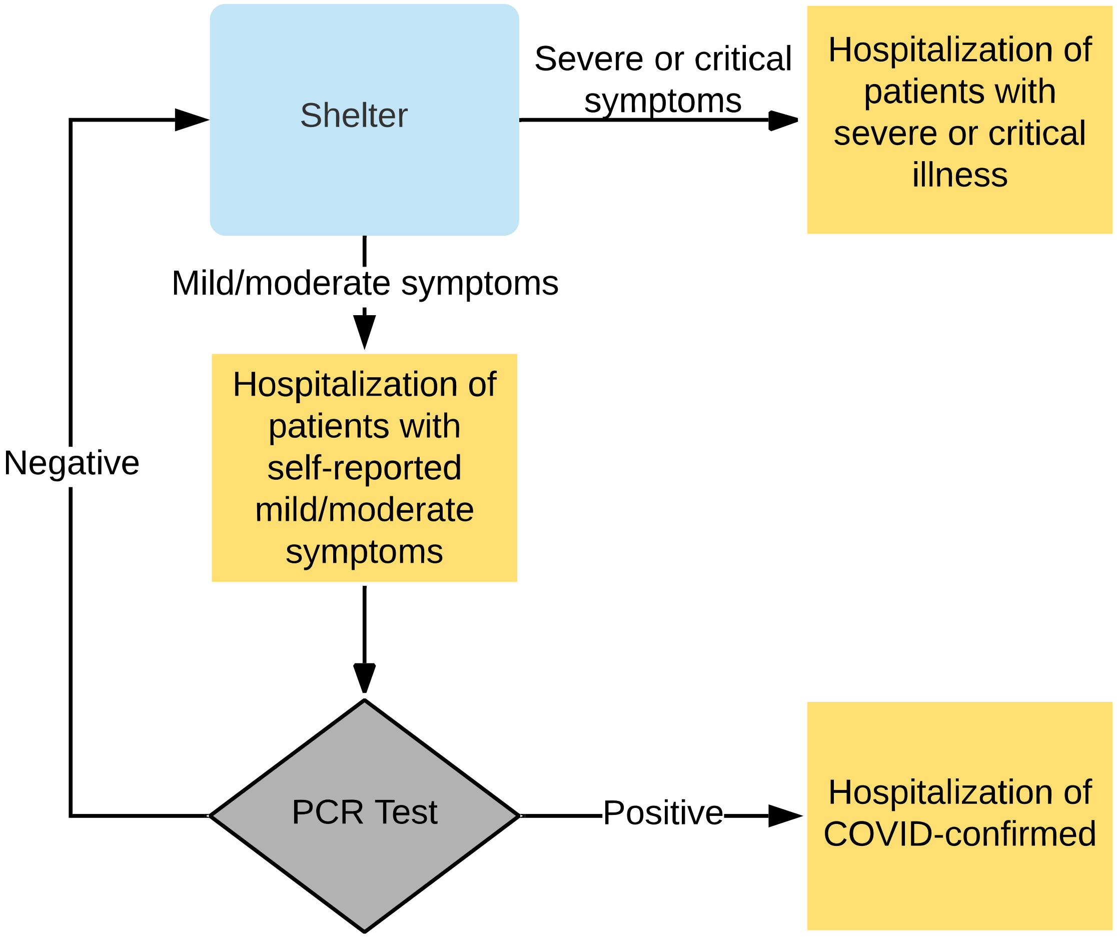

© 2020 Baggett TP et al. *JAMA Network Open.*

2. SxScreen/PCR/Hospital

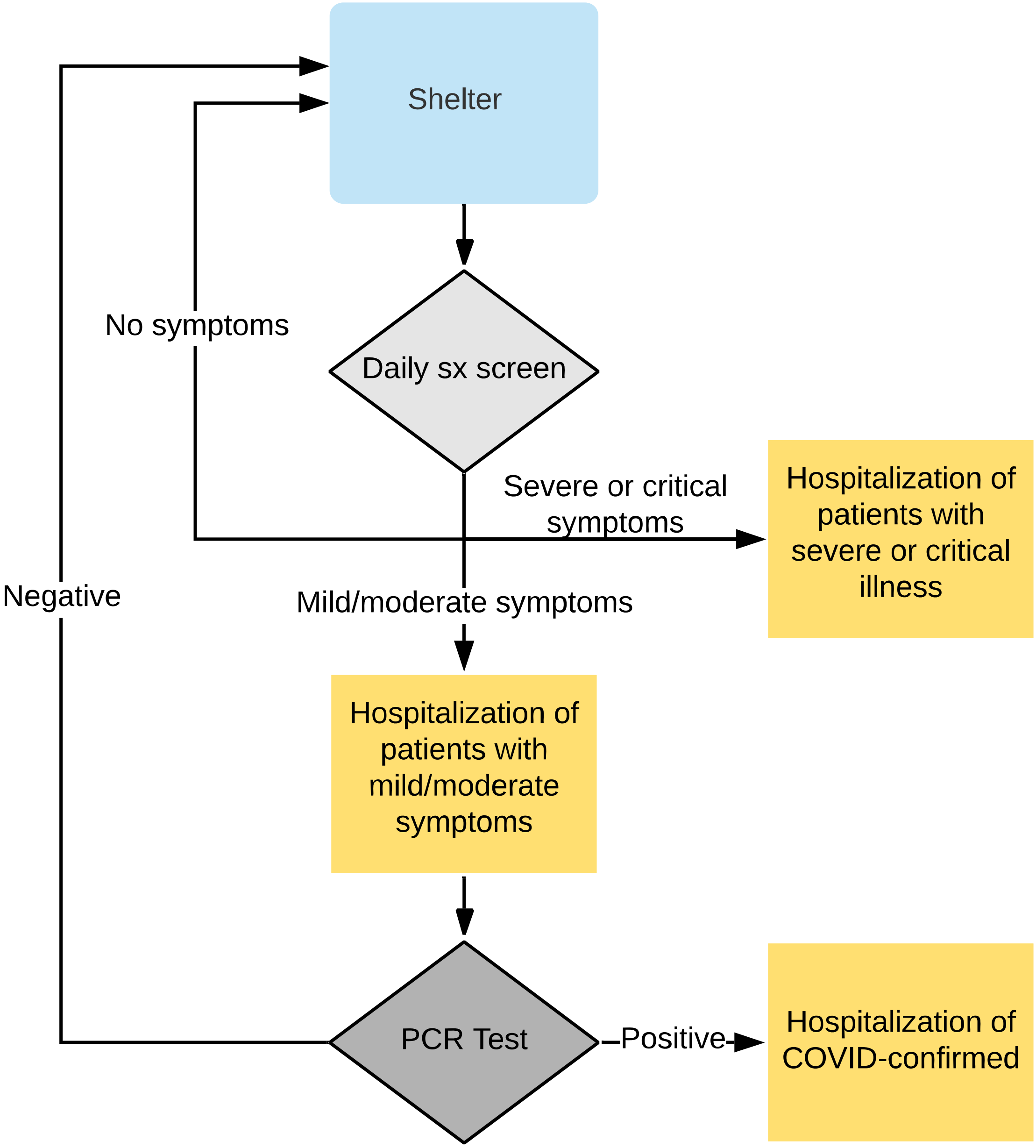

© 2020 Baggett TP et al. *JAMA Network Open.*

3. SxScreen/PCR/ACS

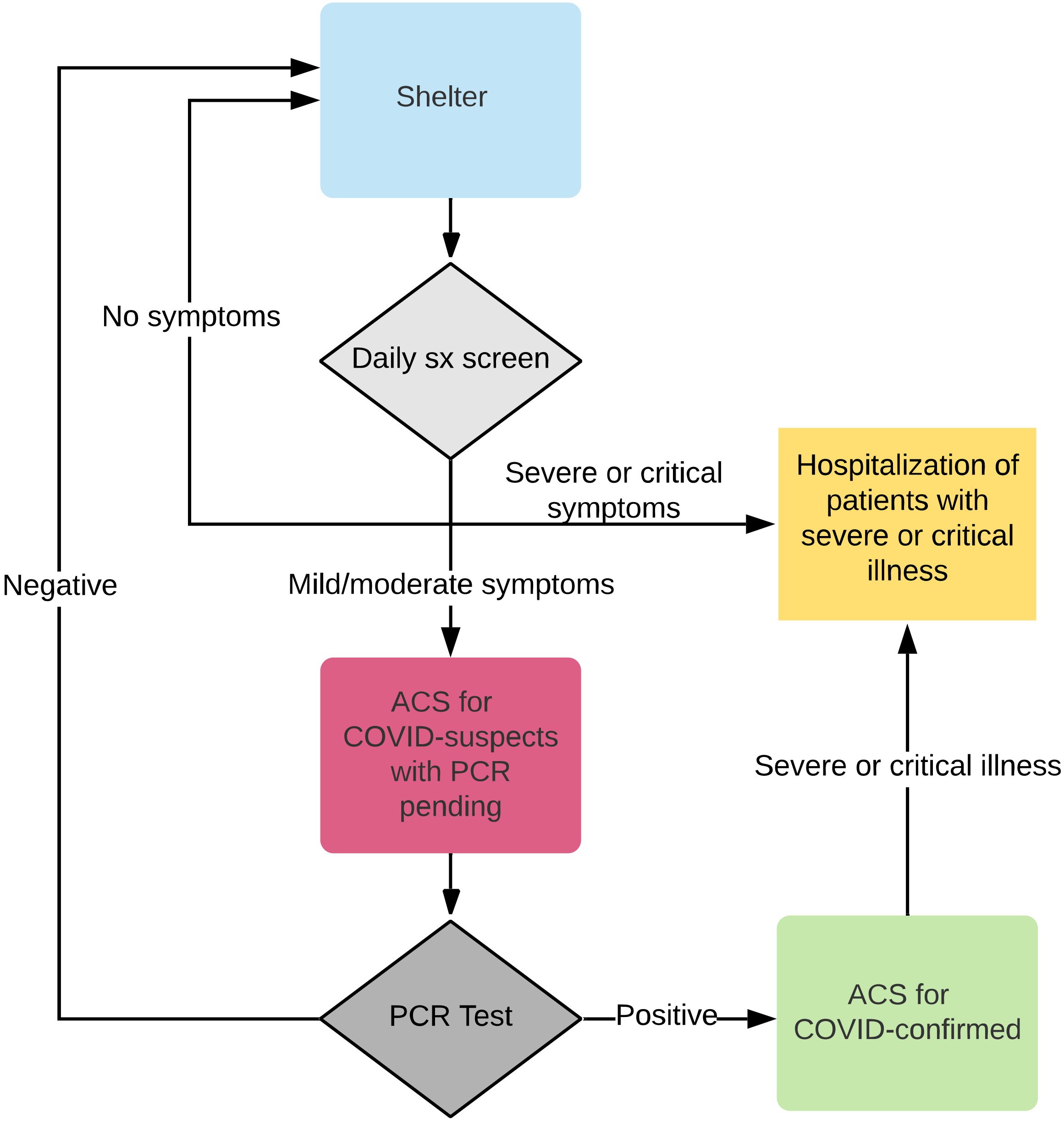

© 2020 Baggett TP et al. *JAMA Network Open.*

4. UniversalPCR/Hospital

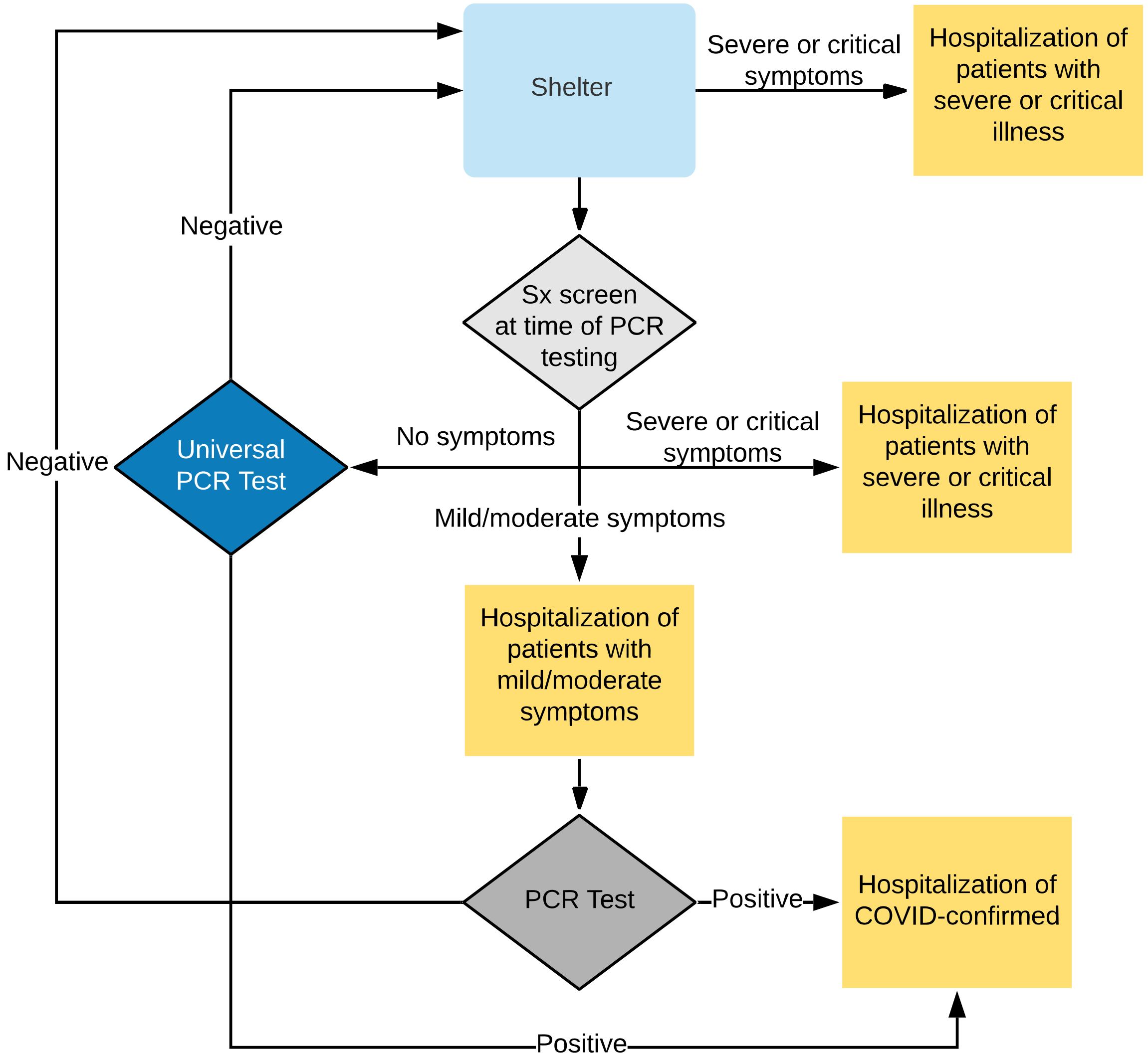

© 2020 Baggett TP et al. *JAMA Network Open.*

5. UniversalPCR/ACS

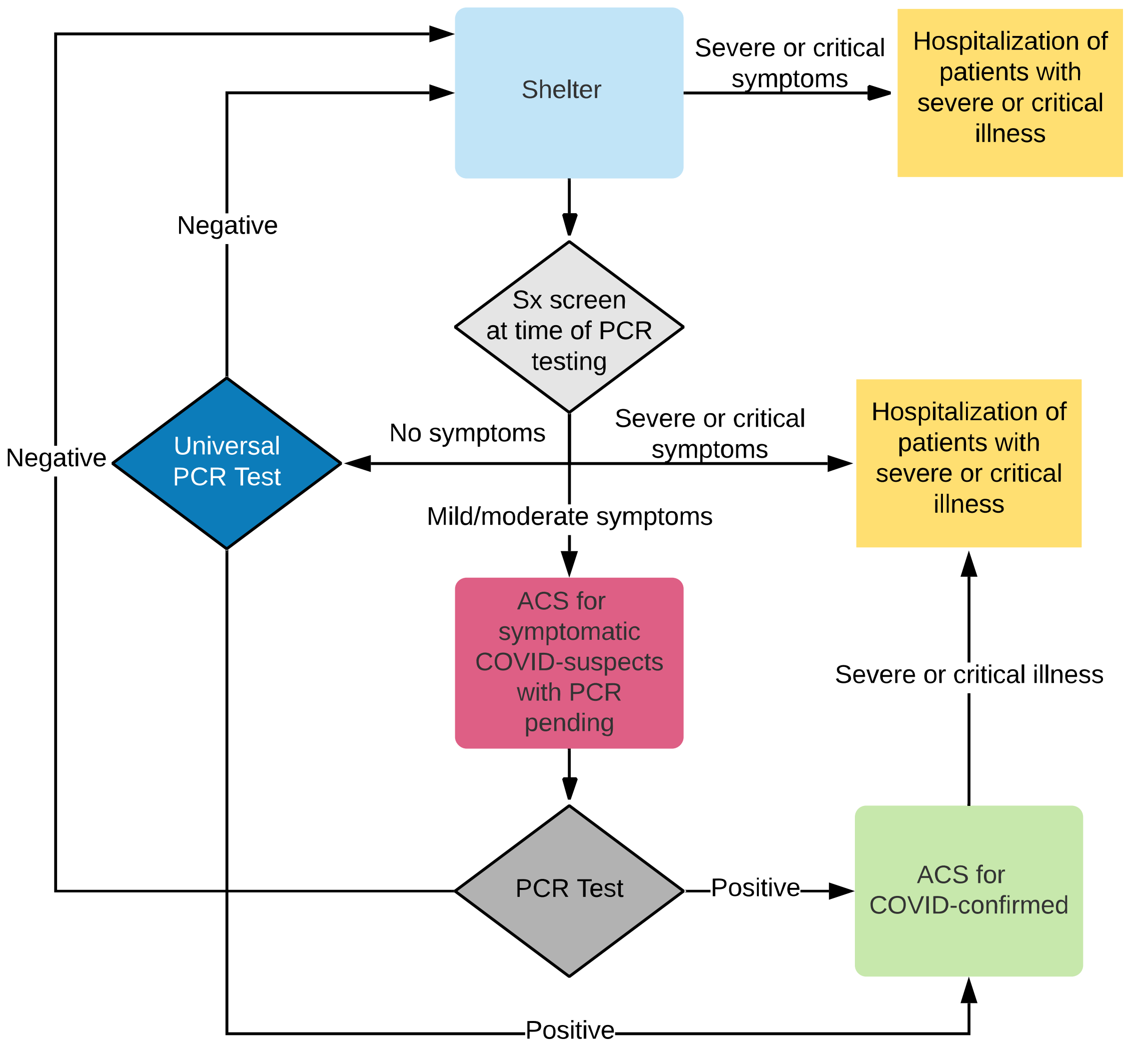

© 2020 Baggett TP et al. *JAMA Network Open.*

6. UniversalPCR/TempHousing

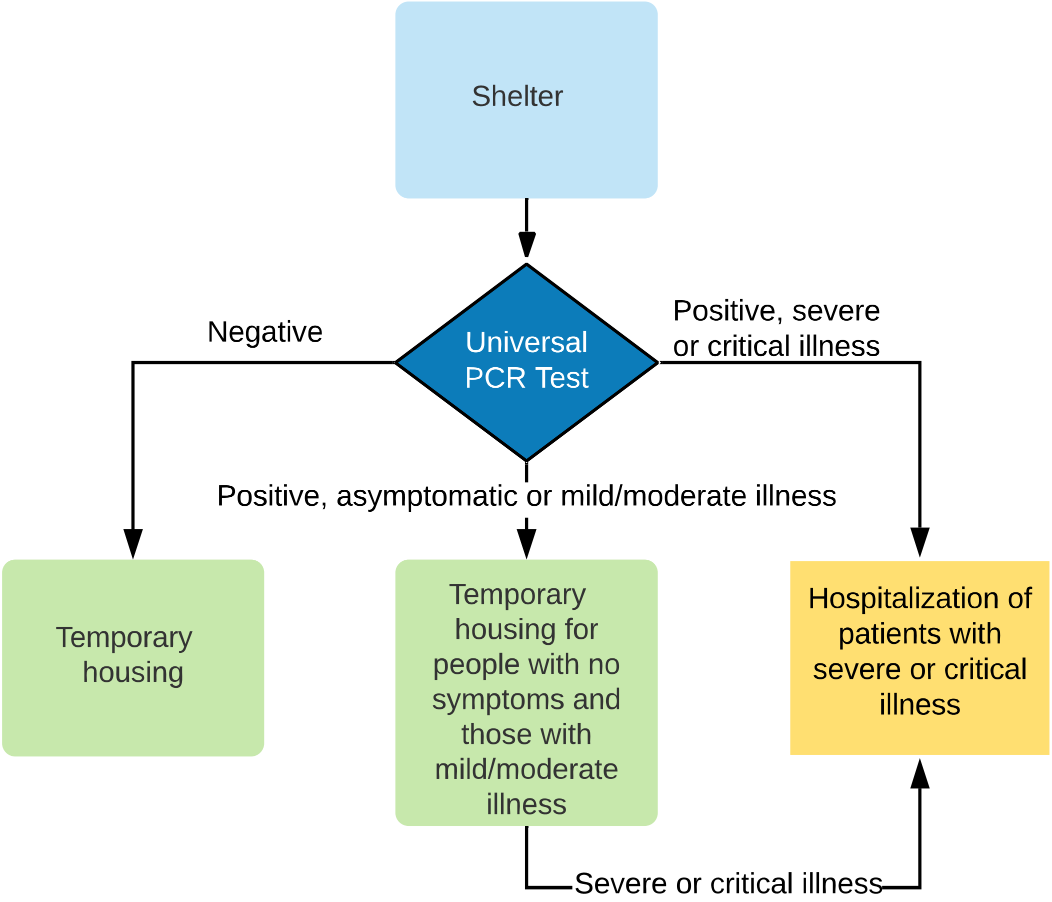

© 2020 Baggett TP et al. *JAMA Network Open.*

7. Hybrid/Hospital

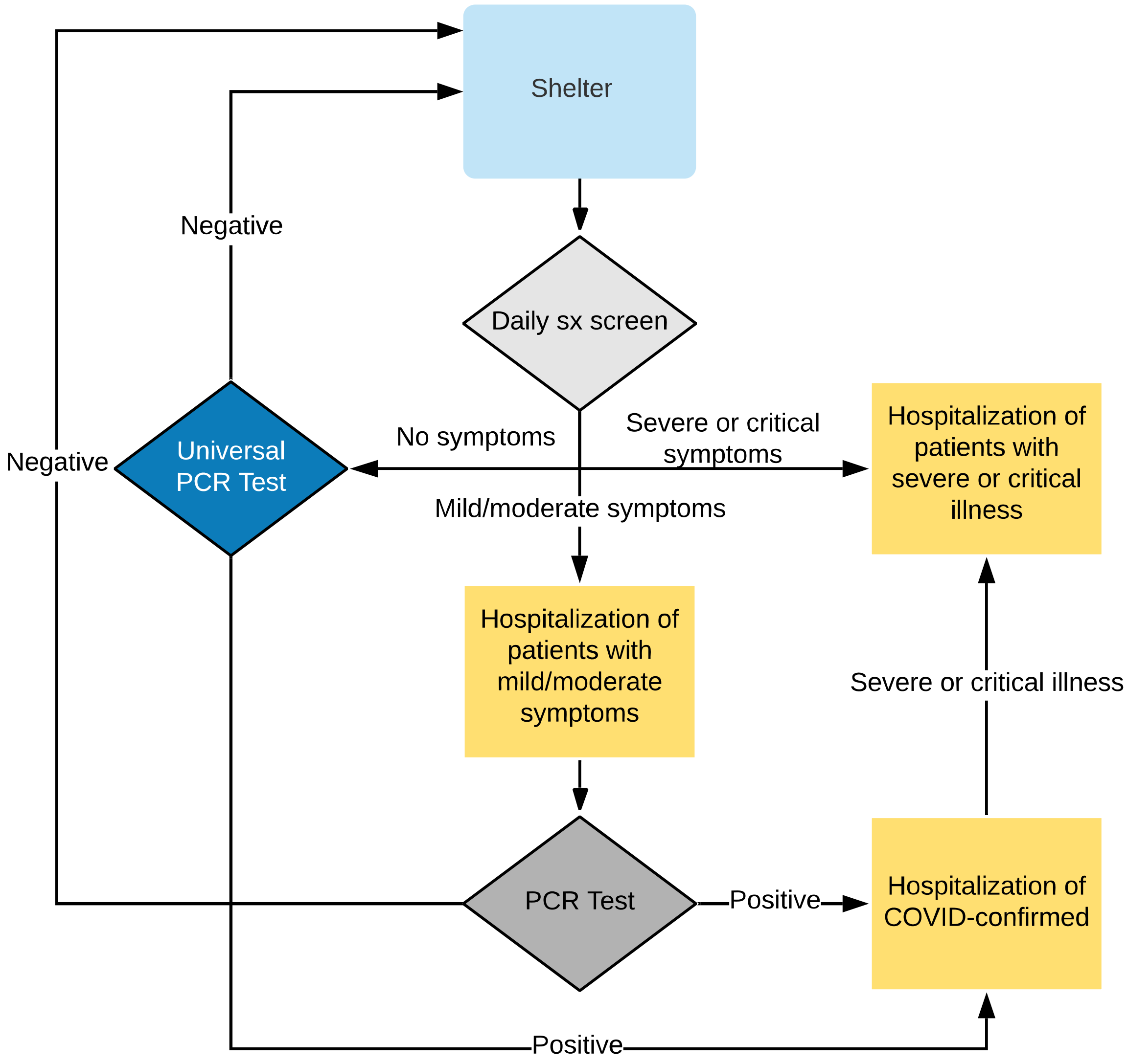

© 2020 Baggett TP et al. *JAMA Network Open.*

8. Hybrid/ACS

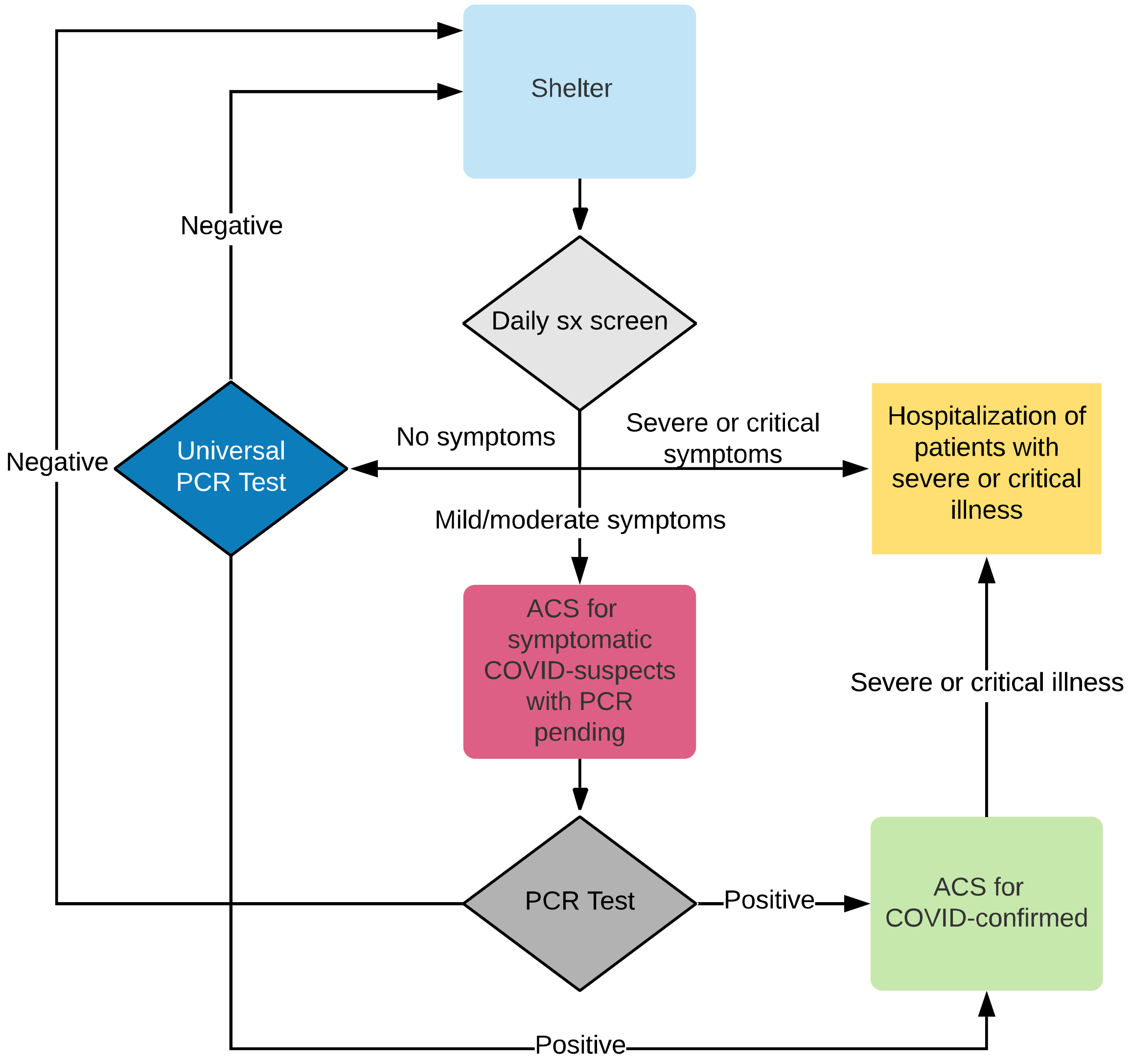

© 2020 Baggett TP et al. *JAMA Network Open.*
